# Trans-ethnic kidney function association study reveals putative causal genes and effects on kidney-specific disease aetiologies

**DOI:** 10.1038/s41467-018-07867-7

**Published:** 2019-01-03

**Authors:** Andrew P. Morris, Thu H. Le, Haojia Wu, Artur Akbarov, Peter J. van der Most, Gibran Hemani, George Davey Smith, Anubha Mahajan, Kyle J. Gaulton, Girish N. Nadkarni, Adan Valladares-Salgado, Niels Wacher-Rodarte, Josyf C. Mychaleckyj, Nicole D. Dueker, Xiuqing Guo, Yang Hai, Jeffrey Haessler, Yoichiro Kamatani, Adrienne M. Stilp, Gu Zhu, James P. Cook, Johan Ärnlöv, Susan H. Blanton, Martin H. de Borst, Erwin P. Bottinger, Thomas A. Buchanan, Sylvia Cechova, Fadi J. Charchar, Pei-Lun Chu, Jeffrey Damman, James Eales, Ali G. Gharavi, Vilmantas Giedraitis, Andrew C. Heath, Eli Ipp, Krzysztof Kiryluk, Holly J. Kramer, Michiaki Kubo, Anders Larsson, Cecilia M. Lindgren, Yingchang Lu, Pamela A. F. Madden, Grant W. Montgomery, George J. Papanicolaou, Leslie J. Raffel, Ralph L. Sacco, Elena Sanchez, Holger Stark, Johan Sundstrom, Kent D. Taylor, Anny H. Xiang, Aleksandra Zivkovic, Lars Lind, Erik Ingelsson, Nicholas G. Martin, John B. Whitfield, Jianwen Cai, Cathy C. Laurie, Yukinori Okada, Koichi Matsuda, Charles Kooperberg, Yii-Der Ida Chen, Tatjana Rundek, Stephen S. Rich, Ruth J. F. Loos, Esteban J. Parra, Miguel Cruz, Jerome I. Rotter, Harold Snieder, Maciej Tomaszewski, Benjamin D. Humphreys, Nora Franceschini

**Affiliations:** 1 Department of Biostatistics, University of Liverpool, Liverpool, L69 3GL UK; 2 Wellcome Centre for Human Genetics, University of Oxford, Oxford, OX3 7BN UK; 3 Department of Medicine, Division of Nephrology, University of Virginia, Charlottesville, VA 22908 USA; 4 Division of Nephrology, Washington University School of Medicine, St Louis, MO 63110 USA; 5 Division of Cardiovascular Sciences, Faculty of Medicine, Biology and Health, University of Manchester, Manchester, M13 9PT UK; 6 Department of Epidemiology, University of Groningen, University Medical Center Groningen, P.O. Box 30.001, 9700 RB Groningen, Netherlands; 7 MRC Integrative Epidemiology Unit, Population Health Sciences, University of Bristol, Bristol, BS8 1TH UK; 8 Department of Pediatrics, University of California, San Diego, San Diego, CA 92161 USA; 9 Charles Bronfman Institute for Personalized Medicine, Icahn School of Medicine at Mount Sinai, New York, NY 10029 USA; 10 Division of Nephrology and Department of Medicine, Icahn School of Medicine at Mount Sinai, New York, NY 10029 USA; 11 Unidad de Investigación Médica en Bioquímica, Hospital de Especialidades, Centro Médico Nacional Siglo XXI, Instituto Mexicano del Seguro Social, Mexico City, 06720 Mexico; 12 Unidad de Investigación Médica en Epidemiologia Clinica, Hospital de Especialidades, Centro Médico Nacional Siglo XXI, Instituto Mexicano del Seguro Social, Mexico City, 06720 Mexico; 13 Center for Public Health Genomics, University of Virginia School of Medicine, Charlottesville, VA 22908 USA; 14 John P Hussman Institute for Human Genomics, University of Miami Miller School of Medicine, Miami, FL 33124 USA; 15 Institute for Translational Genomics and Population Sciences, Departments of Pediatrics and Medicine, Los Angeles Biomedical Research Institute at Harbor-UCLA Medical Center, Torrance, CA 90502 USA; 16 Division of Public Health Sciences, Fred Hutchinson Cancer Research Center, Seattle, WA 98109-1024 USA; 17 Laboratory for Statistical Analysis, RIKEN Center for Integrative Medical Sciences, Yokohama, Kanagawa 230-0045 Japan; 18 Department of Biostatistics, University of Washington, Seattle, WA 98195 USA; 19 Genetic Epidemiology Laboratory, QIMR Berghofer Medical Research Institute, Brisbane, QLD 4006 Australia; 20 Department of Neurobiology, Care Sciences and Society, Division of Family Medicine and Primary Care, Karolinska Institutet, Huddinge, 141 83 Sweden; 21 School of Health and Social Studies, Dalarna University, Falun, 791 88 Sweden; 22 Dr John T Macdonald Department of Human Genetics, University of Miami, Miami, FL 33124 USA; 23 Department of Internal Medicine, Division of Nephrology, University of Groningen, University Medical Center Groningen, P.O. Box 30.001, 9700 RB Groningen, Netherlands; 24 Department of Medicine, Division of Endocrinology and Diabetes, Keck School of Medicine of USC, Los Angeles, CA 90033 USA; 25 School of Health and Life Sciences, Federation University Australia, Ballarat, VIC 3350 Australia; 26 Department of Cardiovascular Sciences, University of Leicester, Leicester, LE1 7RH UK; 27 Department of Physiology, University of Melbourne, Parkville, VIC 3010 Australia; 28 Department of Internal Medicine, Fu Jen Catholic University Hospital, School of Medicine, Fu Jen Catholic University, New Taipei City, 242 Taiwan; 29 Department of Pathology, Erasmus Medical Center Rotterdam, P.O. Box 2040, 3000 CA Rotterdam, Netherlands; 30 Department of Medicine, Division of Nephrology, College of Physicians and Surgeons, Columbia University, New York, NY 10032 USA; 31 Department of Public Health and Caring Sciences, Molecular Geriatrics, Uppsala University, Uppsala, 751 85 Sweden; 32 Department of Psychiatry, Washington University in St Louis, St Louis, MO 63110 USA; 33 David Geffen School of Medicine, University of California Los Angeles, Los Angeles, CA 90024 USA; 34 Los Angeles Biomedical Research Institute at Harbor UCLA Medical Center, Torrance, CA 90502 USA; 35 Department of Medicine and Nephrology, Loyola University Medical Center, Maywood, IL 60153 USA; 36 Laboratory for Genotyping Development, RIKEN Center for Integrative Medical Sciences, Yokohama, Kanagawa 230-0045 Japan; 37 Department of Medical Sciences, Clinical Epidemiology, Uppsala University, Uppsala, 751 85 Sweden; 38 Li Ka Shing Centre for Health Information and Discovery, Big Data Institute, Nuffield Department of Medicine, University of Oxford, Oxford, OX3 7FZ UK; 39 Broad Institute of Harvard and MIT, Boston, MA 02142 USA; 40 Brisbane Institute for Molecular Bioscience, University of Queensland, St. Lucia, QLD 4072 Australia; 41 Epidemiology Branch, Division of Cardiovascular Sciences, National Heart, Lung and Blood Institute, Bethesda, MD 20892 USA; 42 Department of Pediatrics, Division of Genetic and Genomic Medicine, University of California, Irvine Orange, CA 92868 USA; 43 Departments of Neurology and Public Health Sciences, Miller School of Medicine, University of Miami, Miami, FL 33136 USA; 44 Evelyn F McKnight Brain Institute, Miller School of Medicine, University of Miami, Miami, FL 33136 USA; 45 Jackson Memorial Hospital, University of Miami, Miami, FL 33136-1096 USA; 46 Institute of Pharmaceutical and Medicinal Chemistry, Heinrich Heine University Düsseldorf, Düsseldorf, 40225 Germany; 47 Department of Research and Education, Kaiser Permanente Southern California, Pasadena, CA 91101 USA; 48 Department of Medicine, Division of Cardiovascular Medicine, Stanford University School of Medicine, Stanford, CA 94309 USA; 49 Stanford Cardiovascular Institute, Stanford University, Stanford, CA 94309 USA; 50 Stanford Diabetes Research Center, Stanford University, Stanford, CA 94305 USA; 51 Department of Medical Sciences, Molecular Epidemiology and Science for Life Laboratory, Uppsala University, Uppsala, 751 85 Sweden; 52 Collaborative Studies Coordinating Center, Department of Biostatistics, University of North Carolina at Chapel Hill, Chapel Hill, NC 27599-7420 USA; 53 Department of Statistical Genetics, Osaka University Graduate School of Medicine, Osaka, Suita 565-0871 Japan; 54 Laboratory of Molecular Medicine, Human Genome Center, Institute of Medical Science, University of Tokyo, Tokyo, 108-8639 Japan; 55 Mindich Child Health and Development Institute, Icahn School of Medicine at Mount Sinai, New York, NY 10029 USA; 56 Department of Anthropology, University of Toronto at Mississauga, Mississauga, ON L5L 1C6 Canada; 57 Division of Medicine, Manchester University NHS Foundation Trust, Manchester Academic Health Science Centre, Manchester, M13 9WL UK; 58 Department of Epidemiology, University of North Carolina, Chapel Hill, NC 27516-8050 USA

## Abstract

Chronic kidney disease (CKD) affects ~10% of the global population, with considerable ethnic differences in prevalence and aetiology. We assemble genome-wide association studies of estimated glomerular filtration rate (eGFR), a measure of kidney function that defines CKD, in 312,468 individuals of diverse ancestry. We identify 127 distinct association signals with homogeneous effects on eGFR across ancestries and enrichment in genomic annotations including kidney-specific histone modifications. Fine-mapping reveals 40 high-confidence variants driving eGFR associations and highlights putative causal genes with cell-type specific expression in glomerulus, and in proximal and distal nephron. Mendelian randomisation supports causal effects of eGFR on overall and cause-specific CKD, kidney stone formation, diastolic blood pressure and hypertension. These results define novel molecular mechanisms and putative causal genes for eGFR, offering insight into clinical outcomes and routes to CKD treatment development.

## Abstract (web summary)

Estimated glomerular filtration rate (eGFR) is a measure of kidney function used to define chronic kidney disease. Here, Morris et al. perform trans-ethnic genome-wide meta-analyses for eGFR in 312,468 individuals and identify novel loci and downstream putative causal genes.

## Introduction

Chronic kidney disease (CKD) affects ~10% of the global population, with considerable racial/ethnic differences in prevalence and risk factors^1,2^. CKD is associated with premature cardiovascular disease and mortality, and has enormous healthcare costs for treatment, prescriptions and hospitalizations^3–6^. The underlying mechanisms for CKD predisposition and development are unknown, limiting progress in the identification of prognostic biomarkers or the advancement of treatment interventions.

Large-scale genome-wide association studies (GWAS) of estimated glomerular filtration rate (eGFR), a measure of kidney function used to define CKD, have mostly been undertaken in populations of European^7–9^ and East Asian^10^ ancestry. Despite the success of these GWAS in identifying loci contributing to kidney function and risk of CKD, the common single nucleotide variants (SNVs) driving the association signals explain no more than ~4% of the observed-scale heritability of eGFR, and efforts to replicate these findings in other ancestry groups have been limited^11^. Furthermore, efforts to localise the variants driving eGFR association signals at these loci, and the putative causal genes through which their effects are mediated, have been hampered by the extensive linkage disequilibrium (LD) across common variation in European and East Asian ancestry populations.

To enhance understanding of the genetic contribution to kidney function and CKD across diverse populations, and to inform global public health and personalised medicine, we recently established the Continental Origins and Genetic Epidemiology Network Kidney (COGENT-Kidney) Consortium. We undertook trans-ethnic meta-analysis of eGFR GWAS in 71,638 individuals ascertained from populations of African, East Asian, European and Hispanic/Latino ancestry^12^. These investigations provided no evidence of heterogeneity in allelic effects on eGFR association signals between ancestry groups, emphasizing the power of trans-ethnic GWAS meta-analysis for locus discovery that will be relevant to diverse populations.

To further extend characterization of the genetic contribution to eGFR, and determine the molecular mechanisms and putative causal genes through which association signals impact on kidney function, we expand the COGENT-Kidney Consortium in this investigation by assembling GWAS in up to 312,468 individuals of diverse ancestry. With these data, we identify novel loci and distinct associations for kidney function, assess the evidence for heterogeneity in their allelic effects on eGFR, and determine genomic annotations in which these signals are enriched. We identify high-confidence variants driving eGFR association signals through annotation-informed trans-ethnic fine-mapping, and highlight putative causal genes through which their effects are mediated via integration with expression in kidney tissue. Finally, we evaluate the causal effects of eGFR on clinically-relevant renal and cardiovascular outcomes through Mendelian randomisation (MR) with our expanded catalogue of kidney function loci.

## Results

### Study overview

We assembled GWAS in up to 312,468 individuals from three sources (Methods): (i) 19 studies of diverse ancestry from the COGENT-Kidney Consortium, expanding the previously published trans-ethnic meta-analysis^12^ to include additional individuals of Hispanic/Latino descent; (ii) a published meta-analysis of 33 studies of European ancestry from the CKDGen Consortium^9^; and (iii) a published study of East Asian ancestry from the Biobank Japan Project^10^. Each GWAS was imputed up to the Phase 1 integrated 1000 Genomes Project reference panel^13^, and SNVs passing quality control were tested for association with eGFR, calculated from serum creatinine, accounting for age, sex and ethnicity, as appropriate (Methods).

The current study represented a 2.2-fold increase in sample size over the largest published GWAS of kidney function^10^. Assuming homogeneous allelic effects on eGFR across populations, we had more than 80% power to detect an association (*p* < 5 × 10^−8^) with SNVs explaining at least 0.0127% of the trait variance under an additive genetic model. This corresponded to common/low-frequency SNVs with minor allele frequency (MAF) ≥5%/≥0.5% that decrease eGFR by ≥0.0366/≥0.113 standard deviations.

### Trans-ethnic meta-analysis

To discover novel loci contributing to kidney function in diverse populations, we first aggregated eGFR association summary statistics across studies through trans-ethnic meta-analysis (Methods). We employed Stouffer’s method, implemented in METAL^14^, because allelic effect sizes were reported on different scales in each of the three sources contributing to the meta-analysis. We identified 93 loci attaining genome-wide significant evidence of association with eGFR (*p* < 5 × 10^−8^), including 20 mapping outside regions previously implicated in kidney function (Supplementary Figure 1, Supplementary Table 1). The strongest novel associations (Table 1) mapped to/near *MYPN* (rs7475348, *p* = 8.6 × 10^−19^), *SHH* (rs6971211, *p* = 6.5 × 10^−13^), *XYLB* (rs36070911, *p* = 2.3 × 10^−11^) and *ORC4* (rs13026220, *p* = 3.1 × 10^−11^).

**Table 1:** Novel loci attaining genome-wide significant evidence (*p* < 5 × 10^−8^) of association with eGFR in trans-ethnic meta-analysis of up to 312,468 individuals of diverse ancestry

Locus	Lead SNV	Chr	Position (bp, b37)	Alleles		EAF	Fixed-effects meta-analysis			
				Effect^a^	Other		*p*-value	*N*	Beta^b^	SE^b^
*PMF1-BGLAP*	rs2842870	1	156,200,671	T	C	0.632	1.2 × 10^−8^	312,468	−0.361	0.094
*NT5C1B-RDH14*	rs13417750	2	18,681,365	A	G	0.189	1.0 × 10^−8^	312,468	−0.439	0.108
*C2orf73*	rs1527649	2	54,581,356	C	T	0.234	1.5 × 10^−9^	311,225	−0.413	0.107
*ORC4*	rs13026220	2	148,586,459	G	A	0.366	3.1 × 10^−11^	312,468	−0.265	0.095
*NFE2L2*	rs35955110	2	178,143,371	C	T	0.435	3.9 × 10^−9^	312,468	−0.353	0.099
*XYLB*	rs36070911	3	38,498,439	G	A	0.528	2.3 × 10^−11^	312,468	−0.296	0.091
*AK125311*	rs856563	7	46,723,510	C	T	0.750	5.1 × 10^−10^	309,287	−0.455	0.094
*SHH*	rs6971211	7	155,664,686	T	C	0.417	6.5 × 10^−13^	309,287	−0.350	0.090
*NRG1*	rs4489283	8	32,399,662	T	C	0.296	1.5 × 10^−8^	311,632	−0.325	0.094
*TRIB1*	rs2001945	8	126,477,978	C	G	0.546	1.6 × 10^−9^	312,468	−0.264	0.091
*DCAF12*	rs61237993	9	34,130,435	G	A	0.666	4.0 × 10^−8^	312,465	−0.345	0.122
*MYPN*	rs7475348	10	69,965,177	C	T	0.607	8.6 × 10^−19^	312,468	−0.366	0.095
*CYP26A1*	rs4418728	10	94,839,724	T	G	0.539	1.4 × 10^−8^	312,468	−0.345	0.092
*FAM53B*	rs4962691	10	126,424,137	T	C	0.571	5.0 × 10^−10^	312,468	−0.291	0.093
*RASGRP1*	rs9920185	15	39,273,575	C	A	0.649	1.0 × 10^−8^	312,468	−0.332	0.094
*NFAT5*	rs11641050	16	69,622,104	C	T	0.697	2.6 × 10^−8^	312,468	−0.283	0.099
*JUND-LSM4*	rs8108623	19	18,408,519	A	C	0.695	4.4 × 10^−8^	309,634	−0.390	0.108
*ARFRP1*	rs1758206	20	62,336,334	T	C	0.082	2.4 × 10^−8^	163,534	−0.546	0.193
*NRIP1*	rs2823139	21	16,576,783	A	G	0.293	3.7 × 10^−9^	311,637	−0.197	0.093
*ATP50*	rs2834317	21	35,356,706	A	G	0.108	9.5 × 10^−10^	312,468	−0.475	0.126

Chr: chromosome, EAF: effect allele frequency, SE: standard error

^a^Effect allele is aligned to be eGFR decreasing allele

^b^Beta/SE are obtained from fixed-effects meta-analysis, with inverse variance weighting of allelic effect sizes, of up to 81,829 individuals of diverse ancestry from the COGENT-Kidney Consortium, and represent absolute decrease in eGFR (ml/min per 1.73m^2^) per effect allele

Across the 93 loci, we then delineated 127 distinct association signals (at locus-wide significance, *p* < 10^−5^) through approximate conditional analyses implemented in GCTA^15^ (Methods), each arising from different underlying causal variants and/or haplotype effects (Supplementary Tables 1 and 2). The most complex genetic architecture was observed at *SLC22A2* and *UMOD-PDILT*, where the eGFR association was delineated to four distinct signals at each locus (Supplementary Figure 2). Genome-wide, application of LD Score regression^16^ to a meta-analysis of only European ancestry studies revealed the observed scale heritability of eGFR to be 7.6%, of which 44.7%/5.4% was attributable to variation in the known/novel loci reported here (Methods).

### Trans-ethnic heterogeneity in eGFR association signals

To assess the evidence for a genetic contribution to ethnic differences in CKD prevalence, we investigated differences in eGFR associations across the diverse populations contributing to our meta-analysis. We performed trans-ethnic meta-regression of allelic effect sizes obtained from GWAS contributing to the COGENT-Kidney Consortium, implemented in MR-MEGA^17^, including two axes of genetic variation that separate population groups as covariates to account for heterogeneity that is correlated with ancestry (Methods, Supplementary Figure 3). Despite substantial differences in allele frequencies at index SNVs for the distinct associations across ethnicities, we observed no significant evidence (*p* < 0.00039, Bonferroni correction for 127 signals) of heterogeneity in allelic effects on eGFR that was correlated with ancestry (Supplementary Tables 2 and 3). Furthermore, all index SNVs had minor allele frequencies >1% in multiple ethnic groups, indicating that the distinct eGFR association signals were not ancestry-specific. These observations are consistent with a model in which causal variants for eGFR as a measure of kidney function are shared across global populations and arose prior to human population migration out of Africa.

### Enrichment of eGFR associations for genomic annotations

To gain insight into the molecular mechanisms that underlie the genetic contribution to kidney function, we investigated genomic signatures of functional and regulatory annotation that were enriched for eGFR associations across the 127 distinct signals. Specifically, we compared the odds of eGFR association for SNVs mapping to each annotation with those that did not map to the annotation (Methods). We began by considering genic regions, as defined by the GENCODE Project^18^, and observed significant enrichment (*p* < 0.05) of eGFR associations in protein-coding exons (*p* = 0.0049), but not in 3’ or 5’ UTRs. We then interrogated chromatin immuno-precipitation sequence (ChIP-seq) binding sites for 161 transcription factors from the ENCODE Project^19^, which revealed significant joint enrichment of eGFR associations for HDAC2 (*p* = 0.0088) and EZH2 (*p* = 0.030). Class I histone deacetylases (including HDAC2) are required for embryonic kidney gene expression, growth and differentiation^20^, whilst EZH2 participates in histone methylation and transcriptional repression^21^. Finally, we considered ten groups of cell-type-specific regulatory annotations for histone modifications (H3K4me1, H3K4me3, H3K9ac and H3K27ac)^22,23^. Significant enrichment of eGFR associations was observed only for kidney-specific annotations (*p* = 7.4 × 10^−14^). In a joint model of these four enriched annotations, the odds of eGFR association for SNVs mapping to protein-coding exons, binding sites for HDAC2 and EZH2, and kidney-specific histone modifications were increased by 3.06-, 2.13-, 1.76- and 4.29-fold, respectively (Supplementary Figure 4).

### Annotation-informed trans-ethnic fine-mapping

We performed trans-ethnic fine-mapping to localise putative causal variants for distinct eGFR association signals that were shared across global populations by taking advantage of differences in the structure of LD between ancestry groups^24^. To further enhance fine-mapping resolution, we incorporated an annotation-informed prior model for causality, upweighting SNVs mapping to the globally enriched genomic signatures of eGFR associations (Methods). Under this prior, we derived credible sets of variants for each distinct signal, which together account for 99% of the posterior probability (π) of driving the eGFR association (Supplementary Table 4). For 40 signals, a single SNV accounted for more than 50% of the posterior probability of driving the eGFR association, which we defined as high-confidence for causality (Supplementary Table 5). We assessed the evidence of association of these high-confidence SNVs with other measures of kidney function and damage in published GWAS^9,10,25^ (Supplementary Table 6). Several SNVs demonstrated nominal associations (*p* < 0.05) with eGFR calculated from cystatin C, blood urea nitrogen and urine albumin creatinine ratio, with the expected direction of effect of the eGFR decreasing allele.

### Putative causal genes at eGFR association signals

We sought to identify the most likely target gene(s) through which the effects of each of the 40 high-confidence SNVs on eGFR were mediated via functional annotation and colocalisation with expression quantitative trait loci (eQTLs) in kidney tissue.

Only four of the SNVs were missense variants (Table 2), encoding *CACNA1S* p.Arg1539Cys (rs3850625, *p* = 2.5 × 10^−9^, π = 99.0%), *CPS1* p.Thr1406Asn (rs1047891, *p* = 1.5 × 10^−29^, π = 98.1%), *GCKR* p.Leu446Pro (rs1260326, *p* = 2.0 × 10^−35^, π = 86.1%) and *CERS2* p.Glu115Ala (rs267738, *p* = 1.7 × 10^−10^, π = 55.3%). Functional annotation of these high-confidence missense variants highlighted predicted deleterious impact of *CPS1* p.Thr1406Asn and *CERS2* p.Glu115Ala (Methods). *CACNA1S* (Calcium Voltage-Gated Channel Subunit Alpha 1s) encodes a subunit of L-type calcium channel located within the glomerular afferent arteriole, is the target of anti-hypertensive dihydropyridine calcium channel blockers (such as amlodipine and nifedipine), and regulates arteriolar tone and intra-glomerular pressure^26^. *CACNA1S* missense mutations cause hypokalemic periodic paralysis^27,28^, malignant hyperthermia^29^ and congenital myopathy^30^. *CACNA1S* is highly expressed in skeletal muscle tissue, raising the possibility that the high-confidence missense variant may influence eGFR through creatinine production. *CPS1* (Carbamoyl-Phosphate Synthase 1) is involved in the urea cycle, where the enzyme plays an important role in removing excess ammonia from cells^31^. *GCKR* (Glucokinase Regulator) produces a regulatory protein that inhibits glucokinase, and the p.Leu446Pro substitution is a highly pleiotropic variant with reported effects on a wide range of phenotypes, including metabolic traits and type 2 diabetes^32^.

**Table 2:** High confidence SNVs driving eGFR associations and putative causal genes through which their effects on kidney function are mediated

Locus	SNV	*p*-value^a^	π	Gene	Supporting evidence
*ANXA9*	rs267738	1.7 × 10^−10^	55.3%	*CERS2*	Encodes p.Gku115Ala (possibly damaging, deleterious)^b^.
*CACNA1S*	rs3850625	2.5 × 10^−9^	99.0%	*CACNA1S*	Encodes p.Arg1539Cys (possibly damaging, deleterious)^b^.
*GCKR*	rs1260326	2.0 × 10^−35^	86.1%	*GCKR*	Encodes p.Leu446Pro (possibly damaging, tolerated)^b^.
*C2orf73*	rs10181201	7.4 × 10^−8^	60.9%	*SPTBN1*	Intronic; differential expression across kidney cell types.
*LRP2*	rs35472707	1.1 × 10^−6^	64.3%	*LRP2*	Intronic; differential expression across kidney cell types.
	rs60641214	5.6 × 10^−8^	64.9%	*LRP2*	Intronic; differential expression across kidney cell types.
*CPS1*	rs1047891	1.5 × 10^−29^	98.1%	*CPS1*	Encodes p.Thr1406Asn (benign, tolerated)^b^.
*PRDM8-FGF5*	rs12509595	4.7 × 10^−16^	57.1%	*FGF5*	Colocalises with lead eQTL SNV.
*RGS14-SLC34A1*	rs3812036	1.0 × 10^−32^	65.0%	*SLC34A1*	Intronic; differential expression across kidney cell types.
*PIP5K1B*	rs2039424	1.3 × 10^−26^	50.7%	*PIP5K1B*	Intronic; differential expression across kidney cell types.
*WDR37*	rs80282103	2.0 × 10^−18^	100.0%	*LARP4B*	Intronic; differential expression across kidney cell types.
*MPPED2*	rs7930738	4.7 × 10^−7^	51.5%	*MPPED2*	Intronic; differential expression across kidney cell types.
*UMOD-PDILT*	rs77924615	1.5 × 10^−54^	100.0%	*UMOD*	Lead eQTL SNV; differential expression across kidney cell types.
				*GP2*	Lead eQTL SNV; differential expression across kidney cell types.
*DPEP1*	rs2460449	4.2 × 10^−9^	97.8%	*DPEP1*	Intronic; differential expression across kidney cell types.
*BCAS3*	rs9895611	8.9 × 10^−28^	100.0%	*BCAS3*	Intronic; differential expression across kidney cell types.
	rs887258	2.7 × 0^−13^	62.2%	*TBX2*	Colocalises with lead eQTL SNV.

*π* posterior probability of association

^a^*p*-values obtained from fixed-effects meta-analysis

^b^PolyPhen2/SIFT predictions

*CERS2* (Ceramide Synthase 2) variants have previously been associated with albuminuria in individuals with diabetes^33^, and interrogation of the Human Protein Atlas^34^ revealed that the CERS2 protein is abundantly expressed in the glomerulus and tubules of the kidney. *Cers2*-deficient mice exhibit changes in the structure of the kidney^35^. We verified that *Cers2* mRNA is expressed in primary podocytes isolated from the mouse using a previously published method^36^ (Methods, Supplementary Figure 5). To gain insight into the potential role of CERS2 in podocyte motility and function, we isolated and grew primary murine podocytes in culture, and exposed them to the CERS2 inhibitor, ST-1074^37,38^ (Methods). We compared the podocyte migration rate among treated and untreated cells using the scratch wound-healing assay (Supplementary Figure 6). Primary podocytes treated with 3 µM concentration of the CESRS2 inhibitor had a lower migration rate than untreated cells, with significantly higher percentages of uncovered areas remaining at 18 h after wound-scratch. Podocytes treated with ST-1074 appeared much more elongated at 18 h. Although we cannot rule out off-target effects of the inhibitor, these preliminary results suggest that *CERS2* may have a functional impact on podocyte biology. However, further studies are needed to determine the specific role of the gene in the kidney, in vivo, in health and disease states.

The remaining 36 high-confidence SNVs mapped to non-coding regions, which we assessed for colocalisation with eQTL from two resources: (i) non-cancer affected healthy kidney tissue obtained from 260 individuals from the TRANScriptome of renaL humAn TissuE (TRANSLATE) Study^39,40^ and The Cancer Genome Atlas (TCGA)^41^; and (ii) kidney biopsies obtained from 134 healthy donors from the TransplantLines Study^42^ (Methods). We observed that high-confidence eGFR SNVs colocalised with lead renal eQTL variants in the TRANSLATE Study and TGCA (Table 2, Supplementary Table 7) for *FGF5* (rs12509595, *p* = 4.7 × 10^−16^, π = 57.1%), *TBX2* (rs887258, *p* = 2.7 × 10^−13^, π = 62.2%), and both *UMOD* and *GP2* for the same signal at the *UMOD-PDILT* locus (rs77924615, *p* = 1.5 × 10^−54^, π = 100.0%). Of these three high-confidence SNVs, rs8872528 was a significant eQTL (defined by 5% false discovery rate) for *TBX2* across multiple tissues in the GTEx Project^43^, whilst the associations of rs12509595 and rs77924615 with an expression of *FGF5* and *UMOD/GP2*, respectively, were specific to kidney. *FGF5* (Fibroblast Growth Factor 5) is expressed during kidney development, but knockout models have not shown a kidney phenotype^44^. *FGF5* has been implicated in GWAS of blood pressure and hypertension^45^, and other fibroblast growth factors are increasingly recognised as contributors to blood pressure regulation through renal mechanisms^40^. *TBX2* (T-Box 2) plays a role in defining the pronephric nephron in experimental models^46^. *UMOD* encodes uromodulin (Tamm-Horsfall protein), the most abundant urinary protein. The eGFR lowering allele at the high-confidence SNV is associated with increased *UMOD* expression (Supplementary Figure 7), which is consistent with previous investigations that demonstrated uromodulin overexpression in transgenic mice leads to salt-sensitive hypertension and the presence of age-dependent renal lesions^47^.

### Mapping genes to kidney cells

**Fig. 1:**
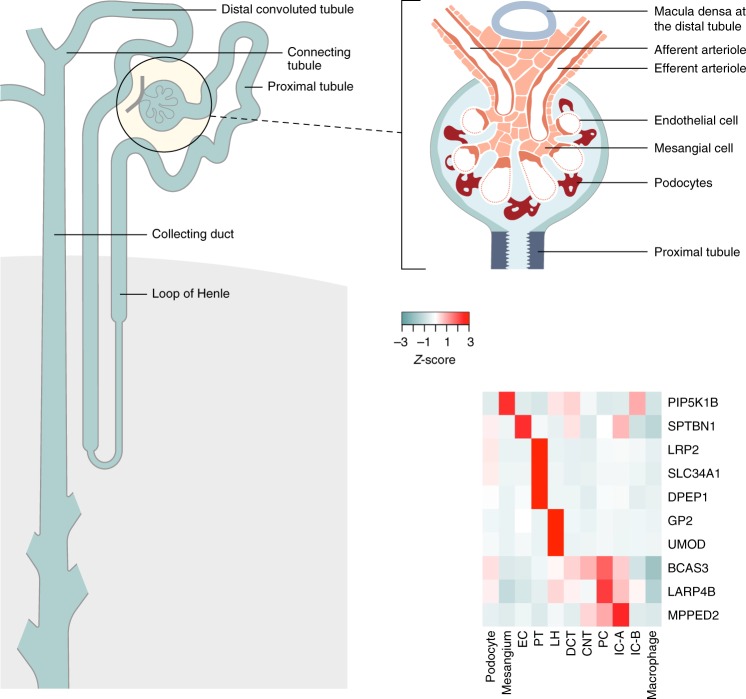
Differential kidney single-cell gene expression in nephron segments. The left and top right panels highlight nephron segments and glomerulus cells, respectively. The heatmap in the bottom right panel presents *Z*-score normalized average gene expression for each specific kidney cell cluster in human adult kidney cells: EC, endothelial cells; PT, proximal tubular cells; LH, loop of Henle cells; DCT, distal convoluted cells; CNT, connecting tubular cells; PC, principal cells; IC-A, intercalate cells type A (located in the collection duct at the distal nephron); IC-B, intercalate cells type B (located in the collection duct at the distal nephron). Source data are provided as a Source Data file

Kidney cells are highly specialised in function based on their location in nephron segments. Previous investigations in mouse and human have revealed that genes at kidney trait-related loci are expressed in a cell-specific manner^48,49^. To provide insight into cellular specificity of the signals at the *UMOD-PDILT*, *FGF5* and *TBX2* loci, we mapped the four genes identified through eQTL analyses to cell types from single nucleus RNA-sequencing (snRNA-seq) data obtained from a healthy human kidney donor (4254 cells, with an average of 1803 detected genes per cell)^49^. *UMOD* and *GP2* demonstrated expression specific to epithelial cells of the ascending loop of Henle (Fig. 1). Uromodulin is involved in protection against urinary tract infections^50^, and the global distribution of *UMOD* regulatory variants in humans correlates with pathogen diversity and prevalence in urine^51^. Glycoprotein 2 is a protein involved in innate immunity. These findings suggest a role for these two proteins in kidney physiology and potential host defence immunity to uropathogens at the *UMOD-PDILT* locus.

By localising high-confidence SNVs to introns and UTRs (Methods), we identified eight additional genes with differential expression across nephron single cell-types (Fig. 1, Table 2): *LRP2*, *SLC34A1* and *DPEP1* (specific to proximal tubule); *SPTBN1* (specific to glomeruli endothelial cells); *PIP5K1B* (specific to glomeruli mesangial cells); and *LARP4B*, *BCAS3*, and *MPPED2* (multiple cell types in the distal nephron). Of these, *DPEP1*, which encodes the protein dipeptidase 1, is implicated in the renal metabolism of glutathione and its conjugates, and regulates leukotriene activity. This localisation fits with the previously suggested connection between glutathione metabolism and defence against chemical injury in proximal tubule cells^52^. Taken together, these findings suggest a potential role of these genes in influencing kidney structure and function through regulation of: (i) glomerular capillary pressure, determining intra-glomerular pressure and glomerular filtration; (ii) proximal tubular reabsorption, affecting tubuloglomerular feedback; or (iii) distal nephron handling of sodium or acid load, influencing kidney disease progression. Additional laboratory-based functional studies will be required to delineate the mechanistic pathways that determine kidney function in healthy and disease states, and potential routes to therapeutic targets for pharmacologic development.

### Causal effects of eGFR on clinically-relevant outcomes

We sought to evaluate the causal effect of eGFR on clinically-relevant kidney and cardiovascular outcomes via two-sample MR^53^ (Methods, Supplementary Tables 8, 9 and 10). Analyses were performed separately in each of the three components of the trans-ethnic meta-analysis because allelic effect sizes were measured on different scales in each. For each trait, we accounted for heterogeneity in causal effects of eGFR via modified *Q*-statistics^54^, excluding outlying genetic instruments that may reflect pleiotropic SNVs and violate the assumptions of MR (Methods, Supplementary Tables 9 and 10).

**Fig. 2:**
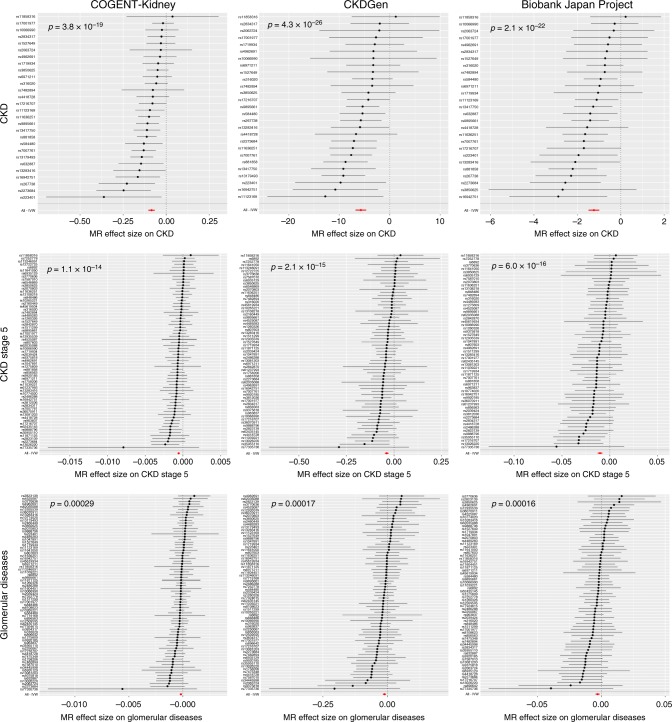
Two-sample MR of eGFR on CKD and cause-specific kidney disease. Results are presented separately for each component of the trans-ethnic meta-analysis for chronic kidney disease (top), chronic kidney disease stage 5 (middle) and glomerular diseases (bottom). Each point corresponds to a lead SNV (instrumental variable) across 94 kidney function loci, plotted according to the MR effect size of eGFR on the outcome (Wald ratio). Bars correspond to the standard errors of the effect sizes. The red point and bar in each plot represents the MR effect size of eGFR on outcome across all SNVs under inverse variance weighted regression. The *p*-values are obtained under inverse variance weighted regression. Results for other methods are presented in Supplementary Table 8

**Fig. 3:**
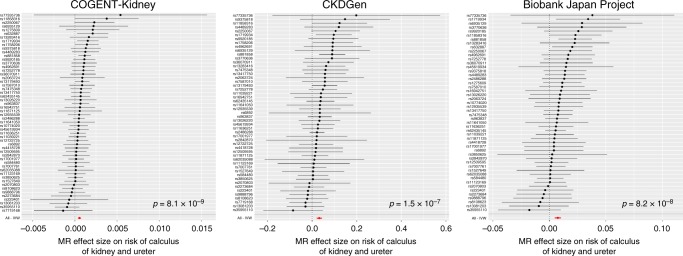
Two-sample MR of eGFR on calculus of kidney and ureter. Results are presented separately for each component of the trans-ethnic meta-analysis. Each point corresponds to a lead SNV (instrumental variable) across 94 kidney function loci, plotted according to the MR effect size of eGFR on calculus of kidney and ureter (Wald ratio). Bars correspond to the standard errors of the effect sizes. The red point and bar in each plot represents the MR effect size of eGFR on calculus of kidney and ureter across all SNVs under inverse variance weighted regression. The *p*-values are obtained under inverse variance weighted regression. Results for other methods are presented in Supplementary Table 8

In each component, we detected a significant (*p* < 0.0042, Bonferroni correction for 12 traits) causal effect of lower eGFR on higher risk of all-cause CKD, glomerular diseases and CKD stage 5, based on reported association summary statistics from the CKDGen Consortium^8^ and the UK Biobank (Fig. 2, Supplementary Table 8). We also detected a significant causal effect of lower eGFR on lower risk of calculus of the kidney and ureter, in each component, based on reported association summary statistics from the UK Biobank (Fig. 3, Supplementary Table 8). The lead eGFR SNV at the *UMOD-PDILT* locus (rs77924615) has been previously associated with kidney stone formation^55^ and is consistent with the role of uromodulin in the inhibition of urine calcium crystallisation^56^. However, this SNV was excluded from the MR analysis due to heterogeneity in effect size and was therefore not driving the causal eGFR association with risk of calculus of the kidney and ureter (Supplementary Table 9).

**Fig. 4:**
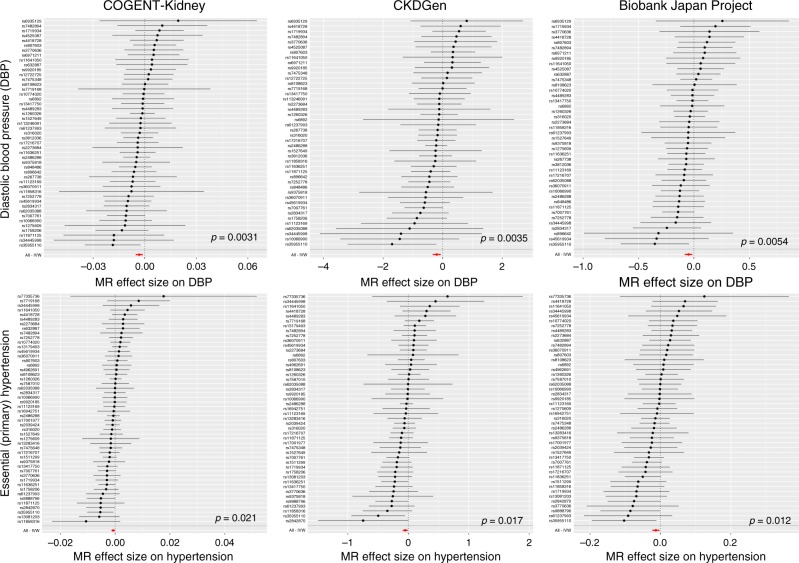
Two-sample MR of eGFR on diastolic blood pressure and hypertension. Results are presented separately for each component of the trans-ethnic meta-analysis for diastolic blood pressure (top) and essential (primary) hypertension (bottom). Each point corresponds to a lead SNV (instrumental variable) across 94 kidney function loci, plotted according to the MR effect size of eGFR on outcome (Wald ratio). Bars correspond to the standard errors of the effect sizes. The red point and bar in each plot represents the MR effect size of eGFR on outcome across all SNVs under inverse variance weighted regression. The *p*-values are obtained under inverse variance weighted regression. Results for other methods are presented in Supplementary Table 8

We also detected a novel causal effect of lower eGFR (at nominal significance, *p* < 0.05, in each component of the trans-ethnic meta-analysis) on higher diastolic blood pressure (DBP) and higher risk of essential (primary) hypertension, but not on systolic blood pressure, based on reported association summary statistics from automated readings and ICD10 codes from primary care data available in the UK Biobank (Fig. 4, Supplementary Table 8). These results are consistent with a role for reduced functional nephron mass on increased peripheral arterial resistance^57^ and confirm previous findings from observational studies^58^. Although the causal association with DBP could not be replicated using published meta-analysis association summary statistics from the International Consortium for Blood Pressure (ICBP)^59^ (Supplementary Table 11), we note that their blood pressure measures were corrected for body-mass index (in addition to age and sex), and there was significant evidence of heterogeneity in effects of eGFR on outcome across SNVs, indicating potential pleiotropy due to collider bias, and consequently invalidating MR estimates. Despite the large sample sizes available for MR analyses from the CardiogramplusC4D Consortium^60^ and MEGASTROKE Consortium^61^, there was no significant evidence of a causal association of eGFR on cardiovascular disease outcomes: coronary heart disease, myocardial infarction or ischemic stroke (Supplementary Table 8).

## Discussion

We identified 20 novel loci for eGFR through trans-ethnic meta-analysis, and dissected 127 distinct association signals that together explain an additional 5.3% of the genome-wide observed scale heritability. The effects of index SNVs for these distinct eGFR association signals were homogeneous across major ancestry groups, which is consistent with a model in which the underlying causal variants are shared across diverse populations, and therefore amenable to trans-ethnic fine-mapping. The localisation of causal variants at eGFR association signals was further enhanced through integration with enriched signatures of genomic annotation that included kidney-specific histone modifications.

We localised high-confidence causal variants driving 40 distinct eGFR association signals, the majority of which have not been previously reported. Through a variety of approaches, including colocalisation with eQTLs in human kidney, and identification of differential expression between human kidney cell types through snRNA-seq, these high-confidence variants implicated several putative causal genes that account for eGFR variation at kidney function loci. Therefore, our strategy of utilising multiple kidney tissue-specific resources to uncover likely causal variants and the genes through which their effects are mediated, followed by mapping of these genes to specific cells in the nephron, provides important biological insight and potential targets for drug development. Knowledge of the specificity of gene expression in nephron segments should also inform future experiments to elucidate the function of some of these genes and potentially define causal molecular mechanisms underlying CKD.

MR analyses of lead SNVs at kidney function loci highlighted previously unreported causal effects of lower eGFR on higher risk of primary glomerular diseases, lower risk of kidney stone formation, and higher DBP and risk of hypertension. The causal relationships of eGFR to these outcomes have been demonstrated to be consistent across ancestries, which is essential for the development of potential interventions that would be relevant to diverse global populations. Our MR analyses also identified lead eGFR SNVs with heterogeneous causal effects on these outcomes, indicating potential pleiotropy. However, further work will be required to determine the specific pathways through which these pleiotropic SNVs act, including non-eGFR determinants of serum creatinine-based eGFR estimating equations.

In conclusion, we have undertaken the most comprehensive trans-ethnic GWAS of eGFR, which has significantly enhanced knowledge of the genetic contribution to kidney function. Our investigation emphasizes the importance of genetic studies of eGFR in diverse populations and their integration with cell-type specific kidney expression data for maximising gains in discovery and fine-mapping of kidney function loci. Taken together, these strategies offer the most promising route to treatment development for a disease with major public health impact across the globe.

## Methods

### Ethics statement

All human research was approved by the relevant institutional review boards and conducted according to the Declaration of Helsinki. All participants provided written informed consent. All mice were maintained on a 12-h light–dark cycle with free access to standard chow and water in the animal facility of the University of Virginia (UVA). Experiments were carried out in accordance with local and NIH guidelines, and the animal protocol was approved by the UVA Institutional Animal Care and Use Committee.

### COGENT-Kidney Consortium: study-level analyses

Study sample characteristics for GWAS from the COGENT-Kidney Consortium, which incorporates 81,829 individuals of diverse ancestry (32.4% Hispanic/Latino, 28.8% European, 28.8% East Asian and 10.0% African American) are presented in Supplementary Table 12. These GWAS included those reported previously^12^ but were expanded with the addition of further studies of Hispanic/Latino ancestry to increase the diversity of represented population groups. Samples were assayed with a range of GWAS genotyping products, and quality control was undertaken within each study (Supplementary Table 13). Samples were excluded because of low genome-wide call rate, extreme heterozygosity, sex discordance, cryptic relatedness, and outlying ethnicity. SNVs were excluded because of low call rate across samples and extreme deviation from Hardy–Weinberg equilibrium. Non-autosomal SNVs were excluded from imputation and association analysis. Within each study, the GWAS genotype scaffold was pre-phased^62,63^ and imputed up to the Phase 1 integrated (version 3) multi-ethnic reference panel from the 1000 Genomes Project^13^ using IMPUTEv2^63,64^ or minimac^63,65^ (Supplementary Table 13). Imputed variants were retained for downstream association analyses if they attained IMPUTEv2 info≥0.4 or minimac *r*^2^ ≥ 0.3.

Within each study, eGFR was calculated from serum creatinine (mg/dL), accounting for age, sex and ethnicity, using the four-variable MDRD equation^66–68^. We tested the association of eGFR with each SNV in a linear regression framework, under an additive dosage model, and with adjustment for study-specific covariates to account for confounding due to population structure (Supplementary Table 13). For each SNV, the association *Z*-score was derived from the allelic effect estimate and corresponding standard error. *Z*-scores and standard errors were then corrected for residual population structure via genomic control^69^ where necessary (Supplementary Table 13).

### CKDGen Consortium: meta-analysis

Full details of the CKDGen Consortium meta-analysis, which incorporated GWAS in 110,517 individuals of European ancestry, have been previously published^9^. Briefly, individuals were assayed with a range of GWAS genotyping products. After quality control, GWAS scaffolds were pre-phased^62,63^ and imputed^63–65^ up to the Phase 1 integrated (version 1 or version 3) multi-ethnic or European-specific reference panels from the 1000 Genomes Project^13^. Imputed variants were retained for downstream association analyses if they attained IMPUTEv2 info≥0.4 or MaCH/minimac *r*^2^≥0.4. Within each study, eGFR was calculated from serum creatinine (mg/dL), accounting for age and sex, using the four-variable Modification of Diet in Renal Disease (MDRD) equation^66–68^. Residuals obtained after regressing ln(eGFR) on age and sex, and study-specific covariates to account for population structure where appropriate, were tested for association with each SNV in a linear regression framework, under an additive dosage model. Association summary statistics within each GWAS were corrected for residual population structure via genomic control^69^ where necessary and were subsequently aggregated across studies, under a fixed-effects model, with inverse-variance weighting of allelic effect sizes, as implemented in METAL^14^.

From the available meta-analysis summary statistics for each SNV (downloaded from http://ckdgen.imbi.uni-freiburg.de/), we derived the association *Z*-score from the ratio of the allelic effect estimate and corresponding standard error. No further correction for population structure was required by genomic control^69^: *λ*_GC_ = 0.977.

### Biobank Japan Project: study-level analysis

Full details of the Biobank Japan Project GWAS, which incorporated 143,658 individuals of East Asian ancestry, have been previously published^10^. Briefly, individuals were assayed with the Illumina HumanOmniExpressExome BeadChip or a combination of the Illumina HumanOmniExpress BeadChip and the Illumina HumanExome BeadChip. After quality control, the GWAS scaffold was pre-phased with MaCH^70^ and imputed up to the Phase 1 integrated (version 3) East Asian-specific reference panel from the 1000 Genomes Project^13^ with minimac^63,65^. Imputed variants were retained for downstream association analyses if they attained minimac *r*^2^ ≥ 0.7. For each individual, eGFR was derived from serum creatinine (mg/dL) using the Japanese coefficient-modified CKD Epidemiology Collaboration (CKD-EPI) equation^71–73^, and adjusted for age, sex, ten principal components of genetic ancestry, and affection status for 47 diseases. The resulting residuals were inverse-rank normalised and tested for association with each SNV in a linear regression framework, under an additive dosage model.

From the available GWAS summary statistics for each SNV (downloaded from http://jenger.riken.jp/en/result), we derived the association *Z*-score from the ratio of the allelic effect estimate and corresponding standard error, and subsequently corrected for residual population structure by genomic control^69^: *λ*_GC_ = 1.252.

### Trans-ethnic meta-analysis

We aggregated eGFR association summary statistics across the three components: COGENT-Kidney Consortium GWAS, the Biobank Japan Project GWAS and the CKDGen Consortium meta-analysis. We performed fixed-effects meta-analysis, with sample size weighting of *Z*-scores (Stouffer’s method), as implemented in METAL^14^, because allelic effect estimates were on different scales in the contributing components. The COGENT-Kidney Consortium included a GWAS of a subset of 23,536 individuals from those contributing to the Biobank Japan Project, which was therefore excluded from the trans-ethnic meta-analysis. Consequently, a combined sample size of 312,468 individuals contributed to the trans-ethnic meta-analysis. SNVs reported in at least 50% of the combined sample size were retained for downstream interrogation. Meta-analysis association summary statistics were corrected for residual population structure via genomic control^69^: *λ*_GC_ = 1.113.

### Locus definition

We first selected lead SNVs attaining genome-wide significant evidence of association (*p* < 5 × 10^−8^) with eGFR in the trans-ethnic meta-analysis that were separated by at least 500kb. Loci were defined by the flanking genomic interval mapping 500kb up- and down-stream of lead SNVs. Where loci overlapped, they were combined as a single locus, and the lead SNV with minimal *p*-value from the meta-analysis was retained.

### Dissection of association signals

To dissect distinct eGFR association signals at loci attaining genome-wide significance in the trans-ethnic meta-analysis, we used an iterative approximate conditional approach, implemented in GCTA^15^. Each COGENT-Kidney Consortium GWAS was first assigned to an ethnic group (Supplementary Table 12) represented in the 1000 Genomes Project reference panel (Phase 3, October 2014 release)^74^. The Biobank Japan Project was assigned to the East Asian ethnic group, and the CKDGen Consortium meta-analysis was assigned to the European ethnic group. Haplotypes in the 1000 Genome Project panel that were specific to the assigned ethnic group were then used as a reference for LD between SNVs across loci for the GWAS in the approximate conditional analysis.

For each locus, we first applied GCTA to the study-level association summary statistics and matched LD reference for each GWAS (or the CKDGen Consortium meta-analysis). We adjusted for the conditional set of variants, which in the first iteration included only the lead SNV at the locus, and aggregated *Z*-scores across studies with sample size weighting (Stouffer’s method) under a fixed-effects model, as implemented in METAL^14^. The conditional meta-analysis summary statistics were corrected for residual population structure using the same genomic control adjustment^69^ as in the unconditional analysis (*λ*_GC_ = 1.113). We defined locus-wide significance by *p* < 10^−5^, which is a Bonferroni correction for the approximate number of (independent) SNVs at each locus. If no SNVs attained locus-wide significant evidence of residual association with eGFR, the iterative approximate conditional analysis for the locus was stopped. Otherwise, the SNV with the strongest residual association signal was added to the conditional set. This iterative process continued, at each stage adding the SNV with the strongest residual association from the meta-analysis to the conditional set, until no remaining SNVs attained locus-wide significance. Note, that at each iteration, studies with missing association summary statistics for any SNV in the conditional set were excluded from the meta-analysis.

For each locus including more than one SNV in the conditional set, we then dissected each distinct association signal. We again applied GCTA to the study-level association summary statistics and matched LD reference for each GWAS (or the CKDGen Consortium meta-analysis), but this time by removing each SNV, in turn, from the conditional set of variants, and adjusting for the remainder. The conditional meta-analysis summary statistics were corrected for residual population structure using the same genomic control adjustment^69^ as in the unconditional analysis (*λ*_GC_ = 1.113). The SNV with the strongest residual association was defined as the index for the signal.

### Estimation of observed scale heritability

We used LD Score regression^16^ to assess the contribution of variation to the observed scale heritability of eGFR. LD Score regression accounts for LD between SNVs on the basis of European ancestry individuals from the 1000 Genomes Project^74^. We therefore performed fixed-effects meta-analysis, with sample size weighting of *Z*-scores (Stouffer’s method), as implemented in METAL^14^, across European ancestry studies from the COGENT-Kidney Consortium and CKDGen Consortium (134,070 individuals), and used these association summary statistics in LD Score regression. We first calculated the contribution of genome-wide variation to the observed scale heritability of eGFR. We then partitioned the genome into previously reported and novel loci attaining genome-wide significance in the trans-ethnic meta-analysis (Supplementary Table 1) and calculated the observed scale heritability of eGFR attributable to each.

### Estimation of allelic effect sizes at index SNVs

Allelic effect estimates were obtained from a meta-analysis of GWAS from the COGENT-Kidney Consortium, including 81,829 individuals of diverse ancestry (Supplementary Table 12), because the other components applied different transformations to eGFR prior to association analysis. The meta-analysis was performed under a fixed-effects model with inverse-variance weighting of effect sizes, implemented in METAL^14^. For loci with multiple signals of association, the allelic effect of an index SNV for each GWAS, prior to meta-analysis, was estimated by application of GCTA^15^ to the study-level association summary statistics and ancestry-matched LD reference, and adjusting for the other index SNVs at the locus. The same approach was used to obtain ethnic-specific allelic effect size estimates by implementing fixed-effects meta-analysis of GWAS within each ancestry group.

### Assessment of heterogeneity in allelic effect sizes

We considered GWAS from the COGENT-Kidney Consortium, including 81,829 individuals of diverse ancestry (Supplementary Table 12), because the other components applied different transformations to eGFR prior to association analysis. We constructed a distance matrix of mean effect allele frequency differences between each pair of GWAS across a subset of SNVs reported in all studies. We implemented multi-dimensional scaling of the distance matrix to obtain two principal components that define axes of genetic variation to separate GWAS from the four major ancestry groups represented in the trans-ethnic meta-analysis. For each SNV, allelic effects on eGFR across GWAS were modelled in a linear regression framework, incorporating the two axes of genetic variation as covariates, and weighted by the inverse of the variance of the effect estimates, implemented in MR-MEGA^17^. Within this modelling framework, heterogeneity in allelic effects on eGFR between GWAS is partitioned into two components. The first component is correlated with ancestry and is accounted for in the meta-regression by the axes of genetic variation, whilst the second is the residual, which is not due to population genetic differences between GWAS.

### Enrichment of eGFR associations in genomic annotations

Within each locus, for each distinct signal, we first approximated the Bayes’ factor^75^ in favour of eGFR association of each SNV on the basis of summary statistics from the trans-ethnic meta-analysis. Specifically, the Bayes’ factor for the *j*th SNV at the *i*th distinct association signal is approximated by

1 $$\Lambda _{ij} = {\mathrm{exp}}\left[ {\frac{{Z_{ij}^2 - {\mathrm{ln}}K}}{2}} \right]$$

where $Z_{ij}^{}$ is the *Z*-score from the trans-ethnic meta-analysis across *K* contributing GWAS. The log-odds of association of the SNV is then given by

2 $${\mathrm{ln}}\left[ {\frac{{\Lambda _{ij}}}{{T_i - \Lambda _{ij}}}} \right]$$

where $T_i = \mathop {\sum }\limits_j \Lambda _{ij}$ is the total Bayes’ factor for the *i*th signal across all SNVs at the locus.

We modelled the log-odds of association of each SNV, for each distinct signal, in a logistic regression framework, as a function of binary variables indicating an overlap with a given genomic annotation. Specifically, for the *j*th SNV at the *i*th distinct association signal,

3 $${\mathrm{ln}}\left[ {\frac{{\Lambda _{ij}}}{{T_i - \Lambda _{ij}}}} \right] = \alpha _i + \beta _kz_{ijk}$$

where $z_{ijk}$ = 1 indicates that the SNV maps to the *k*th annotation, and $z_{ijk}$ = 0 otherwise. In this expression, *α*_*i*_ is a constant for the *i*th distinct association signal, and *β*_*k*_ is the log-fold enrichment in the odds to the association for the *k*th annotation.

We considered three categories of functional and regulatory annotations. First, we considered genic regions, as defined by the GENCODE Project^18^, including protein-coding exons, and 3’ and 5’ UTRs as different annotations. Second, we considered the chromatin immuno-precipitation sequence (ChIP-seq) binding sites for 161 transcription factors from the ENCODE Project^19^. Third, we considered ten groups of cell-type-specific regulatory annotations for histone modifications (H3K4me1, H3K4me3, H3K9ac, and H3K27ac) obtained from a variety of resources^22,23^, which were previously derived for partitioning heritability by annotation by LD Score regression^76^.

Within each category, we first used forward selection to identify annotations that were jointly enriched at nominal significance (*p* < 0.05). We then included all selected annotations across categories in a final model to obtain joint estimates of the fold-enrichment in eGFR association signals for each.

### Trans-ethnic fine-mapping

Within each locus, for each distinct signal, we calculated the posterior probability of driving the eGFR association for each SNV under an annotation-informed prior model, derived from the globally enriched annotations, and the Bayes’ factor approximated from the trans-ethnic meta-analysis. Specifically, for the *j*th SNV at the *i*th distinct association signal, the posterior probability $\pi _{ij} \propto \gamma _{ij}\Lambda _{ij}$. In this expression, the relative annotation informed prior is given by

4 $$\gamma _{ij} = {\mathrm{exp}}\left[ {\mathop {\sum }\limits_k \hat \beta _kz_{ijk}} \right]$$

where the summation is over the selected enriched annotations, and $\hat \beta _k$ is the estimated log-fold enrichment of the *k*th annotation from the final joint model.

We derived a 99% credible set^77^ for the *i*th distinct association signal by: (i) ranking all SNVs according to their posterior probability $\pi _{ij}$; and (ii) including ranked SNVs until their cumulative posterior probability of driving the association attains or exceeds 0.99. Index SNVs accounting for more than 50% posterior probability of driving the eGFR association at a given signal were defined as high-confidence.

### SNV associations with measures of kidney function and damage

We evaluated the evidence for association of high-confidence variants with measures of kidney function and damage from published GWAS: (i) eGFR calculated from cystatin C, obtained from up to 24,061 individuals of European ancestry from the CKDGen Consortium^9^; (ii) blood urea nitrogen, obtained from up to 139,818 individuals of East Asian ancestry from the Biobank Japan Project^10^; and (iii) urine albumin to creatinine ratio, obtained from up to 46,061 non-diabetic individuals of European ancestry from the CKDGen Consortium^25^. Effects on these traits were aligned to the eGFR decreasing allele.

### Functional annotation of high-confidence missense variants

We assessed the predicted functional impact of high-confidence missense variants across a range of databases including FATHMM (functional analyses through hidden Markov models)^78^ and metaSVM (scores for non-synonymous variants based on SVM model)^79^.

### Primary podocyte cell culture and scratch assay

The protocol for isolation and culture of primary podocytes from 129S6 mice has been previously published^36^. Briefly, under general anaesthesia with isoflurane, mice were perfused through the heart with 6 × 10^8^ magnetic beads/ml (Dynabeads, Invitrogen) diluted in 10 ml of phosphate-buffered saline (PBS). The kidneys were extracted, decapsulated and cut into small pieces, then digested in collagenase A (1mg/ml) at 37 ^o^C for 25 min The digest was pressed with a syringe pestle through a 100 uM cell strainer, collected, and washed with 5 mL of PBS, repeated once, and then passed through a 40 uM cell strainer, and washed again with 5 mL of PBS. The isolated glomeruli were then washed off the cell strainer with pre-warmed cell culture media (RPMI 1640 with L-glutamine supplemented with 10% of fetal bovine serum, 100 units/ml of Pen/Strep and 1% of L-glutamine), then further isolated by magnetic particle concentrator (Invitrogen) and washed with a pre-warmed culture media 2–3 times. Glomeruli were then re-suspended in culture media and placed in the 6-well plate coated with rat tail type I collagen and incubated at 37 ^o^C. Light microscopy was used to confirm the characteristic migration of podocytes from glomeruli after 3–5 days.

Approximately after 7–9 days, the primary podocytes reached a confluent monolayer, and a rectangular wound was created by scratching the monolayer from the top to the bottom of the well using the base of a sterile 200 µl pipette tip. Images of the scratched area were immediately taken after wound creation and after an additional 18 h of incubation, using EVOS XL Core Cell Imaging System (×10 magnification). We utilised ST-1074, a potent inhibitor that can be used in lower concentrations to avoid cell toxicity, for which the clinical potential of the class of compounds influencing CerS subtypes has been previously published^37,38^. The inhibitor was added to the well containing 1 mL of culture media, at a concentration of 1 mM in DMSO, immediately after wound creation at a volume of 3 µL (3 µM). For the control condition, 3 µL of DMSO alone was added. At least 3 independent experiments were performed with 3–5 wells per condition. The images were analysed using ImageJ software (1.48v, NIH, USA), by measuring pixels of the outlined scratched area immediately after wound creation and after 18 h. The results are expressed as % of area that was not covered by migrating podocytes after 18 h of incubation time, compared to the scratched area created immediately after wound creation.

We note that in vitro (HCT116), ST-1074 significantly inhibits both CerS2 and CerS4 (screening conditions 10 µM). ST-1074 disclosed an IC50 value for C18:0 of 21.4 µM (CerS4 inhibition), and an IC50 value for C24:1 of 28.7 µM (CerS2 inhibition), which do not show significant difference. In HeLa cells, ST-1074 inhibited at 5 µM CerS2 and CerS4 significantly (C24:1 ~40%, C24:0 ~50%). However, in the Human Protein Atlas^34^, *CERS4* is only expressed in the tubules and not in podocytes. Although ST-1074 is a CerS2 inhibitor, off-target effects may also influence podocyte function.

### Kidney tissue eQTLs: TRANSLATE Study and TGCA

We performed eQTL analysis using data from the TRANSLATE Study^39,40^ and TGCA^41^. In brief, as a source of kidney tissue, both studies used apparently normal samples from European ancestry individuals undergoing nephrectomy due to kidney cancer (the specimens were collected from the cancer-unaffected pole of the organ). The data from both studies were processed in the same manner using procedures described below.

Gene expression was quantified in transcripts per million (TPM) using Kallisto^80^. The quality control included: removing outlier samples^81,82^, checking consistency between declared and biological sex (using XIST and Y-chromosome genes); removing genes on non-autosomal chromosomes; and removing genes with either interquartile range of zero or those not meeting the minimum expression criterion (TPM > 0.1 and read counts ≥6 in at least 30% of samples within each study/sequencing batch). Before *cis*-eQTL analysis, the log_2_-transformed TPM data were normalised using robust quantile normalisation in the R package aroma and then standardised using rank-based inverse normal transformation in GenABEL. To account for technical variation, we used probabilistic estimation of expression residuals (PEER)^83^: 30 latent factors for the TRANSLATE Study and 15 for TCGA as recommended for different sample sizes in the GTEx Project^84,85^.

Kidney DNA samples from individuals from the TRANSLATE Study were genotyped using the Infinium HumanCoreExome-24 BeadChip array, and genotype calls were made using Genome Studio. Individuals from TCGA were genotyped using the Affymetrix Genome-Wide Human SNP Array 6.0, and genotype calls were made using the Birdseed algorithm. Quality control removed variants that: had low genotyping rate (<95%); mapped to Y chromosome/mitochondrial DNA or had an ambiguous chromosomal location; violated Hardy–Weinberg equilibrium (HWE, *p* < 0.001); or had MAF <5%. Quality control also removed individuals with: genotyping call-rate <95%; heterozygosity above/below 3 standard deviations from the mean; cryptic relatedness to other individuals; non-European genetic ancestry; and discordant sex information (inconsistency between declared and genotyped sex). For both studies, the resulting scaffold was imputed up to the Phase 3 multi-ethnic reference panel from the 1000 Genomes Project^74^ using the Michigan Imputation Server^86^. After imputation, we retained only SNVs, removing those with low imputation coefficient (*R*^2^ < 0.4), MAF <5%, or violating HWE (*p* < 10^−6^).

A total of 260 individuals (160 from the TRANSLATE Study and 100 from TCGA) were included in the analysis, involving 15,711 genes and 5,498,156 SNVs common to both studies. Normalised gene expression was modelled as a function of alternate allele dosage via linear regression, including sex, three axes of genetic variation (to account for population structure) and PEER latent factors as additional covariates. The regression coefficients of the alternate allele from the two studies were then combined in a fixed-effects meta-analysis under an inverse-variance weighting scheme. For each gene, only those SNVs in *cis* (within 1 Mb of the transcription start/stop sites) were included in the analysis. A total of 2000 permutations were used to derive the empirical distribution of the smallest *p*-value for each gene, which then was used to adjust the observed smallest *p*-value for the gene. The correction for testing multiple genes was based on false discovery rate (FDR) applied to permutation-adjusted *p*-values (via Storey’s method as implemented in the R package *q*-value) with a cut-off of 5%. Furthermore, the thresholds for nominal *p*-values were derived using a global permutation-adjusted *p*-value closest to FDR of 5% and the empirical distributions determined using permutations.

We identified high-confidence SNVs from the trans-ethnic fine-mapping that were colocalised with lead eQTL variants (i.e. the same SNV or in strong LD, *r*^2^>0.8) at a 5% FDR, and reported the corresponding eGene.

### Kidney tissue eQTLs: TransplantLines Study

We performed eQTL analysis using data from the TransplantLines Study^42^. The study includes kidneys from donors, donated after brain death or cardiac death. Samples were genotyped on the Illumina CytoSNP 12 v2 array and imputed up to the Phase 1 integrated (version 3) multi-ethnic reference panel from the 1000 Genomes Project^13^ using IMPUTEv2^63,64^. Expression and genotype data were available for 236 kidney biopsies obtained from 134 donors, and analyses have been described previously^59^. Briefly, residuals of gene expression for each probe were obtained after adjusting for the first 50 expression principal components to filter out environmental variation^87^. A linear mixed model was used to test the association of residual expression of each probe with the allele dosage of each SNV mapping within 1Mb of the transcription start/stop sites using the R package lme3. Sex, age, donor type, time of biopsy and three axes of genetic variation (to account for population structure) were included in the model as fixed effects. Random effects were then included for donor to account for multiple samples obtained from the same individual.

We identified high-confidence SNVs from the trans-ethnic fine-mapping that were colocalised with lead eQTL variants (i.e. the same SNV or in strong LD, *r*^2^>0.8) at a 5% FDR, and reported the corresponding eGene.

### Expression of GWAS genes across kidney cell-types

We identified genes for which high-confidence SNVs mapped to introns and untranslated regions. We mapped the genes to cell-types from snRNA-seq data generated by 10x Chromium from a healthy human kidney (62-year old white male, no history of CKD and serum creatinine of 1.03 mg/dl)^49^. The kidney was dissected from the cortex and was bulk homogenized using a dounce homogenizer. The dataset included 4524 cells: 7.8% glomerular cells (including podocytes, endothelial cells and mesangial cells); 86.7% tubular cells (including proximal tubule, loop of Henle, distal convoluted tubule, connecting tubule, proximal tubule and intercalate cells); the remaining 5.5% cells are mostly macrophages and novel cells that do not map to known cell types. An average of 1803 genes were detected per cell. We generated a differential expression gene (DEG) list by performing Wilcoxon rank sum tests on each cell-type from the single nucleus dataset. A gene was defined as mapping to a specific kidney cell type if the expression fulfils all the following criteria: (i) present in the DEG list; (ii) expressed in >25% of the total cells in the specified cell-type; and (iii) log-fold change in expression was >0.25 in the specified cell-type when compared to all other cell-types^49^. Gene expression values for each cell were *Z*-score normalised. A new gene expression matrix with mean *Z*-scores for each gene was obtained by averaging the *Z*-scores from all individual cells in the same cluster. The *Z*-score normalized gene expression were presented as a heatmap using the heatmap.2 function in the R package gplots.

### Two-sample MR analyses

We performed a lookup of association summary statistics for lead SNVs at each of the eGFR loci across a range of clinically-relevant kidney and cardiovascular outcomes from public and proprietary data resources. These included: CKD (12,385 cases and 104,780 controls, published data from the CKDGen Consortium^8^); IgA nephropathy (3211 cases and 8735 controls, unpublished data); glomerular diseases (ICD10 N00-N08, 2,289 cases and 449,975 controls, extracted UK Biobank using GeneATLAS); CKD stage 5 (ICD10 N18, 4905 cases and 447,359 controls, extracted from UK Biobank using GeneATLAS); hypertensive renal disease (ICD10 I12, 1663 cases and 450,601 controls, extracted from UK Biobank using GeneATLAS); calculus of kidney and ureter (ICD10 N20, 5216 cases and 447,048 controls, extracted from UK Biobank using GeneATLAS); DBP (317,756 individuals, automated reading, extracted from UK Biobank using MR-BASE^88^); systolic blood pressure (317,654 individuals, automated reading, extracted from UK Biobank using MR-BASE^88^); essential (primary) hypertension (ICD10 I10, 84,640 cases and 367,624 controls, extracted from UK Biobank using GeneATLAS); coronary heart disease (60,801 cases and 123,504 controls, published data from the CardiogramplusC4D Consortium^60^); myocardial infarction (43,676 cases and 128,199 controls, published data from the CardiogramplusC4D Consortium^60^); and ischemic stroke (10,307 cases and 19,326 controls, published data from the MEGASTROKE Consortium^61^).

We performed two-sample MR for each outcome using eGFR as the exposure and the extracted non-palindromic lead SNVs as instrumental variables. The lead SNVs were not in LD with each other, so that their effects on exposure and outcomes were uncorrelated. Analyses were performed separately in each of the three components of the trans-ethnic meta-analysis because allelic effect sizes were measured on different scales in each: COGENT-Kidney Consortium (58,293 individuals after excluding those from the Biobank Japan Project); CDKGen Consortium (110,517 individuals); and Biobank Japan Project (143,658 individuals). For each trait, we first accounted for heterogeneity in causal effects of eGFR via modified *Q*-statistics^54^, implemented in the R package RadialMR, which identified outlying genetic instruments that may reflect pleiotropic SNVs. For each trait, our primary MR analyses were then performed after excluding outlying SNVs in any component of the trans-ethnic meta-analysis using inverse variance weighted regression^89^, implemented in the R package TwoSampleMR^88^. We also assessed the evidence for causal association between exposure and outcome using two additional approaches that are less sensitive to heterogeneity (although less powerful) and implemented in the R package TwoSampleMR^88^: weighted median regression^90^ and MR-EGGER regression^91^.

We performed an additional lookup of association summary statistics for non-outlying lead SNVs at each of the eGFR loci for DBP (150,134 individuals, published data from ICBP^51^). We assessed the evidence for a causal association of eGFR on DBP in each component of the trans-ethnic meta-analysis using inverse variance weighted regression^89^, weighted median regression^90^ and MR-EGGER regression^91^, as implemented in the R package TwoSampleMR^88^.

## Supplementary information

Supplementary Information

Source Data

## Data Availability

Association summary statistics will be made available from: (i) the COGENT-Kidney Consortium component of the trans-ethnic meta-analysis; and (ii) the trans-ethnic meta-analysis across the COGENT-Kidney Consortium, CKDGen Consortium and Biobank Japan Project. Fine-mapping data for each distinct eGFR signal will be made available, including the posterior probability of driving the association for each SNV. These data will be made available via: (i) the University of Liverpool Statistical Genetics and Pharmacogenomics Research Group website (https://www.liverpool.ac.uk/translational-medicine/research/statistical-genetics/data-resources); and (ii) the dbGaP CHARGE Summary Results site^92^ with accession number phs000930. The source data underlying Fig. 1 and Supplementary Figure 7 are provided as a Source Data file.
