# Supplementary material for: Trans-ethnic kidney function association study reveals putative causal genes and effects on kidney-specific disease aetiologies
Source: Nat Commun. 2019 Jan 3;10:29. doi: 10.1038/s41467-018-07867-7 (PMC6318312; doi:10.1038/s41467-018-07867-7)
Supplement: Supplementary file 1 — Supplementary Information [file 41467_2018_7867_MOESM1_ESM.pdf]

Trans-ethnic genome-wide association study of kidney function provides novel insight into effector genes and causal effects on kidney-specific disease aetiologies

Andrew P Morris, Thu H Le, Haojia Wu, Artur Akbarov, Peter J van der Most, Gibran Hemani, George Davey Smith, Anubha Mahajan, Kyle J Gaulton, Girish N Nadkarni, Adan Valladares-Salgado, Niels Wacher-Rodarte, Josyf C Mychaleckyj, Nicole D Dueker, Xiuqing Guo, Yang Hai, Jeffrey Haessler, Yoichiro Kamatani, Adrienne M Stilp, Gu Zhu, James P Cook, Johan Arnlov, Susan H Blanton, Martin H de Borst, Erwin P Bottinger, Thomas A Buchanan, Sylvia Cechova, Fadi J Charchar, Pei-Lun Chu, Jeffrey Damman, James Eales, Ali G Gharavi, Vilmantas Giedraitis, Andrew C Heath, Eli Ipp, Krzysztof Kiryluk, Holly J Kramer, Michiaki Kubo, Anders Larsson, Cecilia M Lindgren, Yingchang Lu, Pamela AF Madden, Grant W Montgomery, George J Papanicolaou, Leslie J Raffel, Ralph L Sacco, Elena Sanchez, Holger Stark, Johan Sundstrom, Kent D Taylor, Anny H Xiang, Aleksandra Zivkovic, Lars Lind, Erik Ingelsson, Nicholas G Martin, John B Whitfield, Jianwen Cai, Cathy C Laurie, Yukinori Okada, Koichi Matsuda, Charles Kooperberg, Yii-Der Ida Chen, Tatjana Rundek, Stephen S Rich, Ruth JF Loos, Esteban J Parra, Miguel Cruz, Jerome I Rotter, Harold Snieder, Maciej Tomaszewski, Benjamin D Humphreys, and Nora Franceschini

SUPPLEMENTARY INFORMATION

Supplementary Figures 1
Supplementary Tables 8
Supplementary Note 38

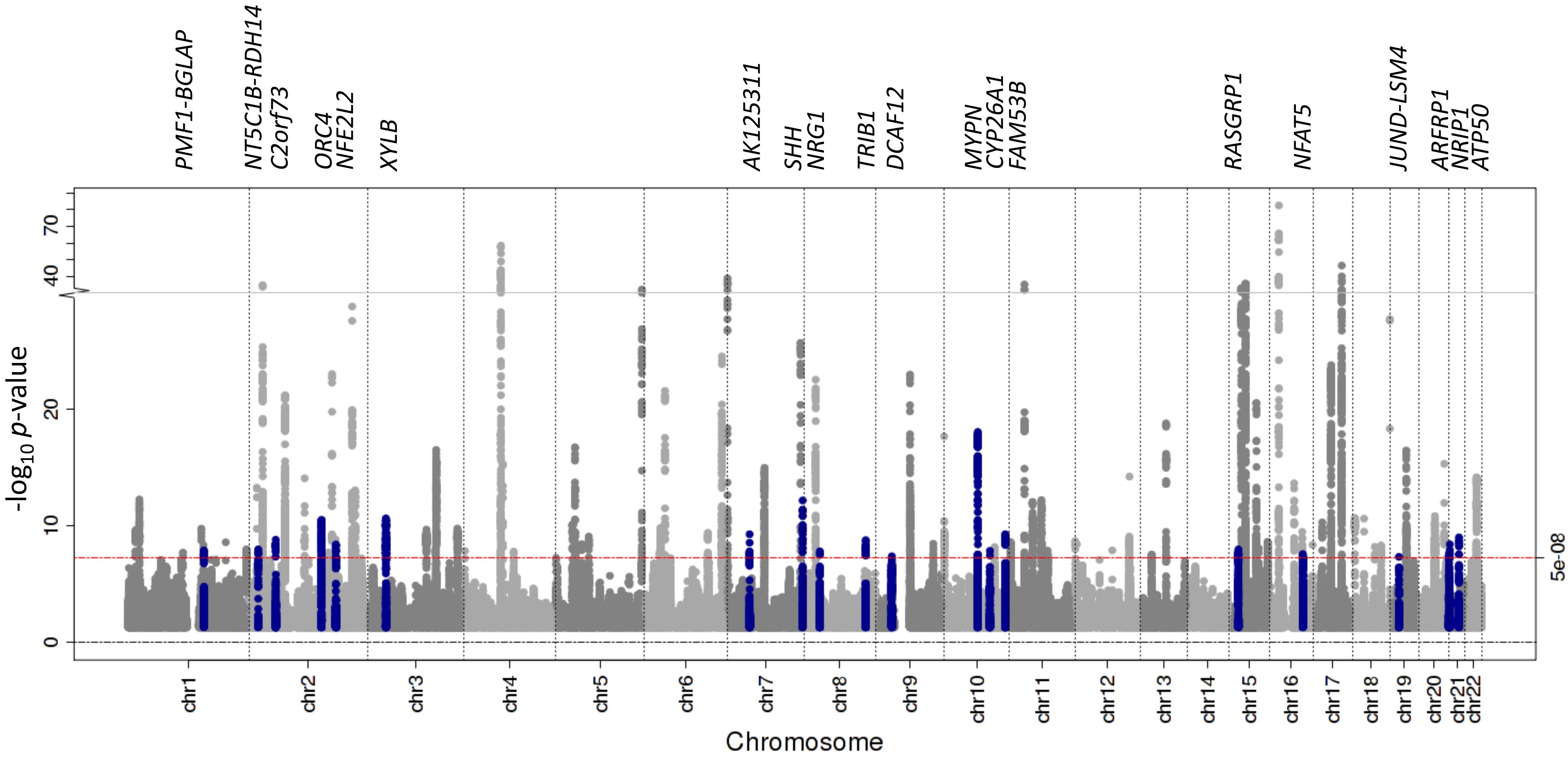

Supplementary Figure 1. Manhattan plot of genome-wide eGFR association. Association summary statistics are based on trans-ethnic meta-analysis of 312,468 individuals. Each point represents a SNV passing quality control in the meta-analysis, plotted with their *p*-value (on a $-\log_{10}$ scale) as a function of genomic position (NCBI build 37). Association signals attaining genome-wide significance ($p<5\times10^{-8}$, horizontal red line) and mapping outside of previously established eGFR loci are highlighted in blue, with locus name presented above the Manhattan plot.

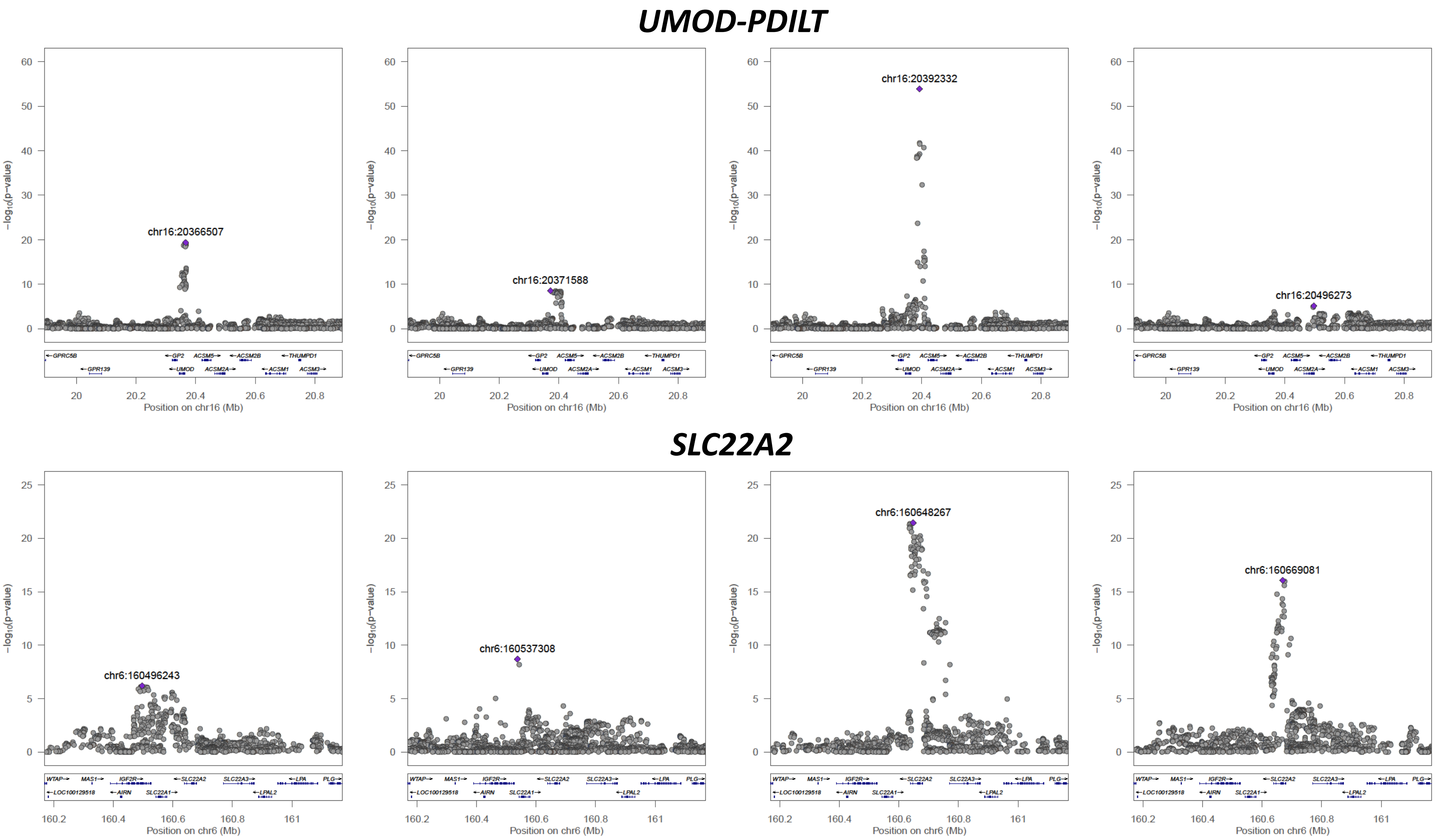

Supplementary Figure 2. Signal plots for distinct eGFR association signals at *UMOD-PDILT* (top) and *SLC22A2* (bottom). Association summary statistics are based on trans-ethnic meta-analysis of 312,468 individuals: results are presented from approximate conditioning after adjusting for all other index SNVs at the locus. Each point represents a SNV passing quality control in the meta-analysis, plotted with their conditional *p*-value (on a $-\log_{10}$ scale) as a function of genomic position (NCBI build 37). In each plot, the index variant is represented by the purple symbol. Gene annotations are taken from the University of California Santa Cruz genome browser.

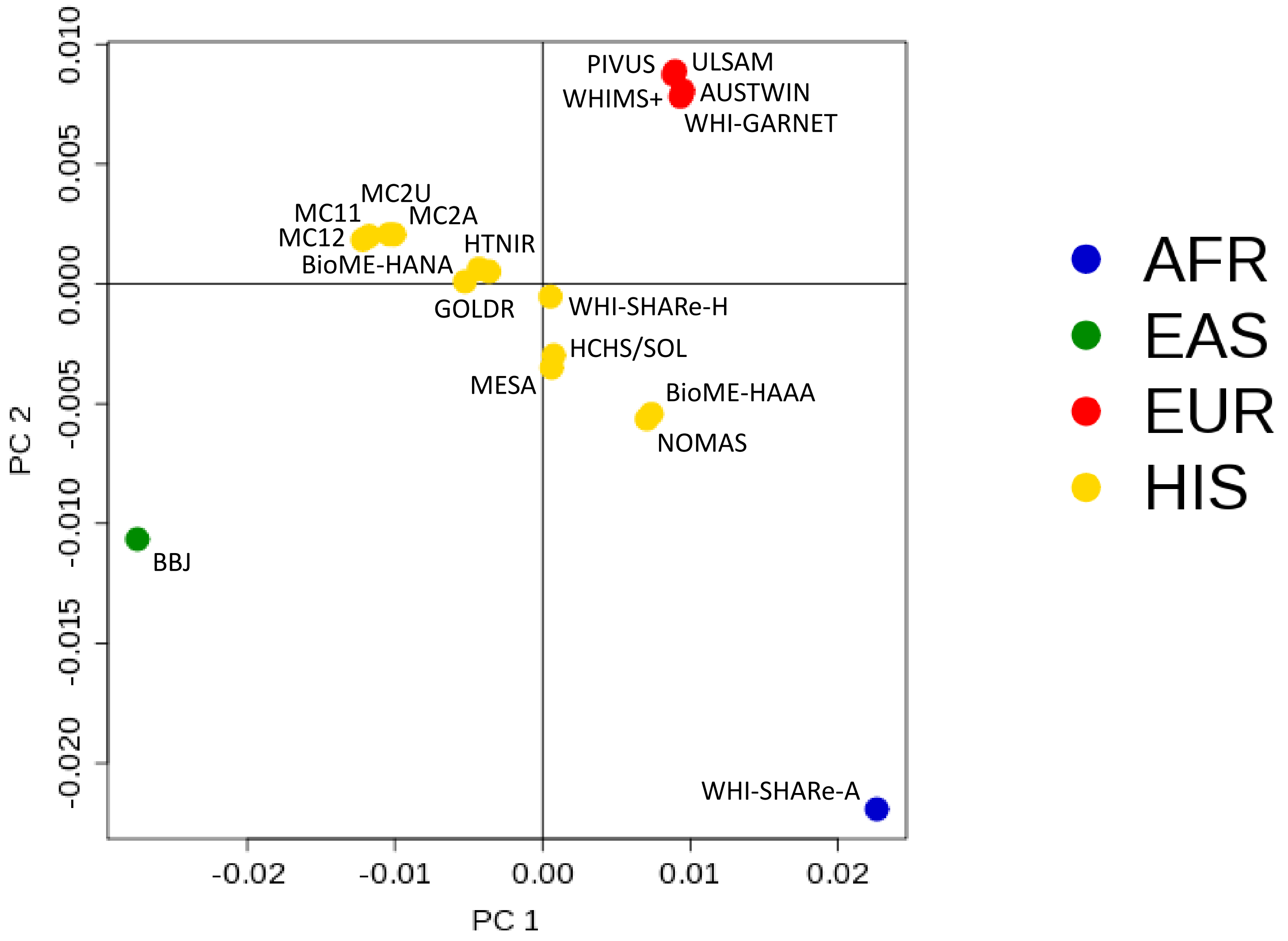

Supplementary Figure 3. Axes of genetic variation separating GWAS of eGFR from diverse populations in the COGENT-Kidney Consortium. The first two axes of genetic variation (PC1 and PC2) from multi-dimensional scaling of the Euclidean distance matrix of mean pairwise allele frequency differences between GWAS are sufficient to separate four ancestry groups: African (AFR), Hispanic/Latino (HIS), East Asian (EAS) and European (EUR). The greatest genetic diversity is observed in the Hispanic/Latino ethnic group: the axes of genetic variation represent a cline from Native American ancestry (low PC1 and high PC2) to African ancestry (high PC1 and low PC2).

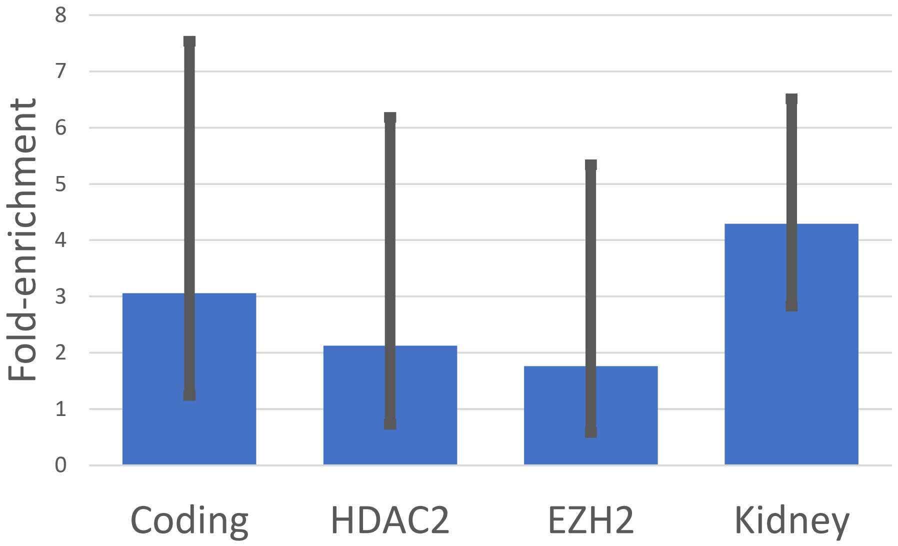

Supplementary Figure 4. Genomic annotations enriched for eGFR association signals. Fold-enrichment (from a joint model) in the odds of eGFR association for each annotation are represented by the blue bars, with corresponding 95% confidence intervals represented by the black lines. Coding: coding exons. HDAC2: binding sites for HDAC2. EZH2: binding sites for EZH2. Kidney: kidney-specific histone modifications.

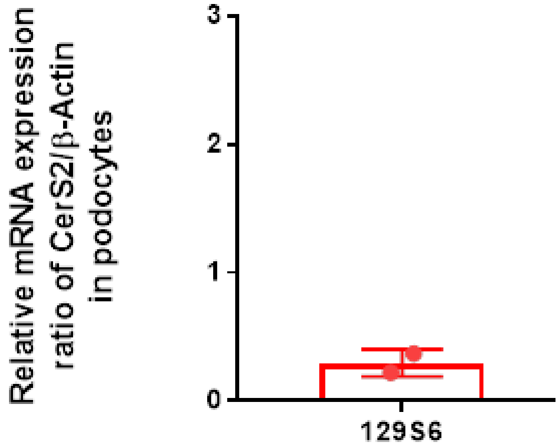

Supplementary Figure 5. Relative mRNA expression of Cers2 to β-actin in podocytes. Primary podocytes were isolated from 129S6 mice and confirmed by mRNA expression of synaptopodin as previously published[36]. Red bar represents mean relative expression, with error bars corresponding to standard deviation.

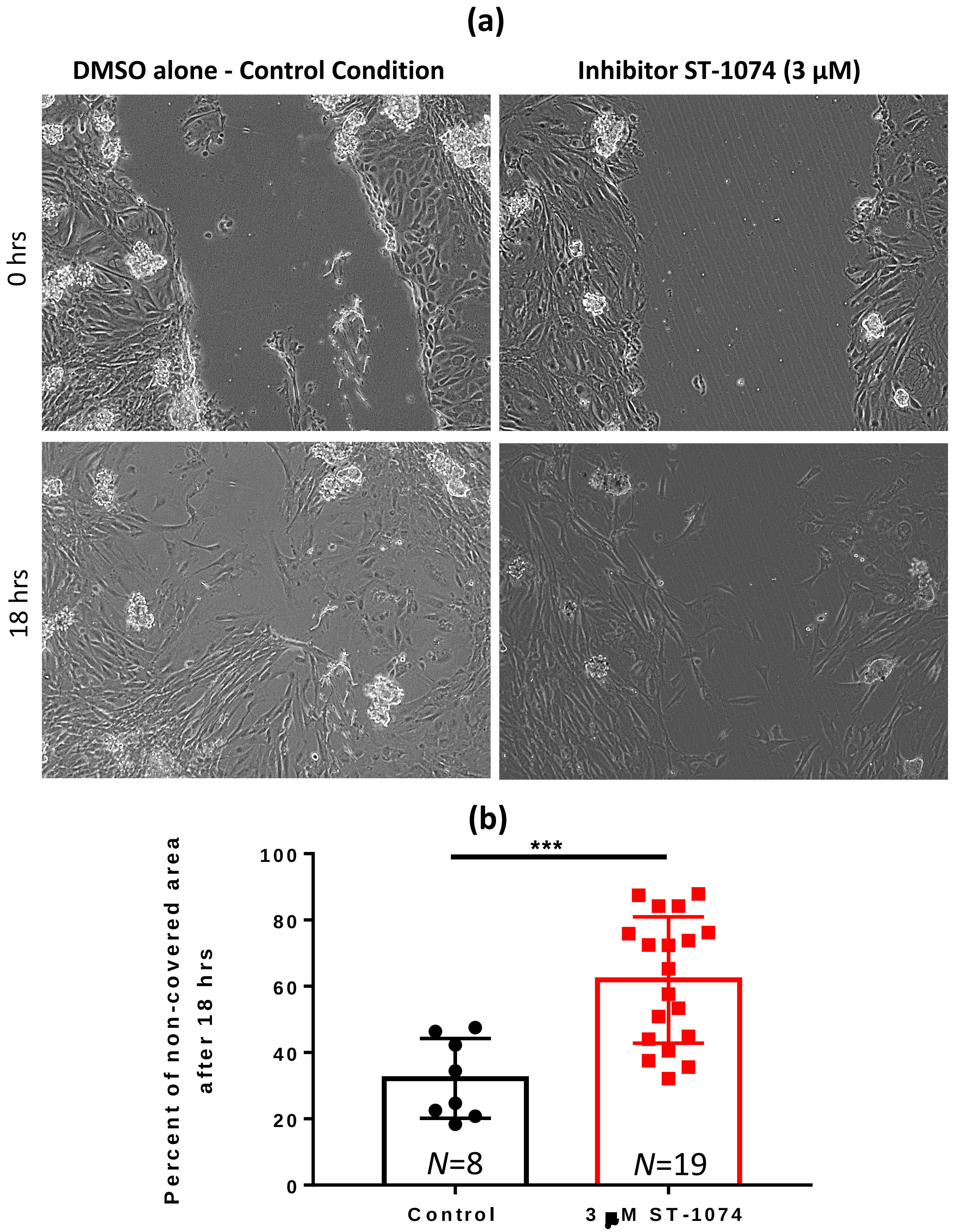

Supplementary Figure 6. Effect of ST-1074 inhibitor on migration and morphology of primary murine podocytes in scratch wound-healing assay in culture. (a) Representative images of outlined scratch area of wound created by base of pipette tip in confluent layer or primary podocytes in culture at time 0 hrs and podocyte migration into the scratched area at time 18 hrs, for control condition (DMSO alone), and for condition treated with compound ST-1074. Pictures were taken by EVOSTMXL Core Cell Imaging System at 10X magnification. (b) Plotted data showing % non-covered area after 18 hrs for each individual scratched well. Black and red bars correspond to mean percentages, and error bars correspond to standard deviation. ***unpaired t-test, two-sided p<0.001.

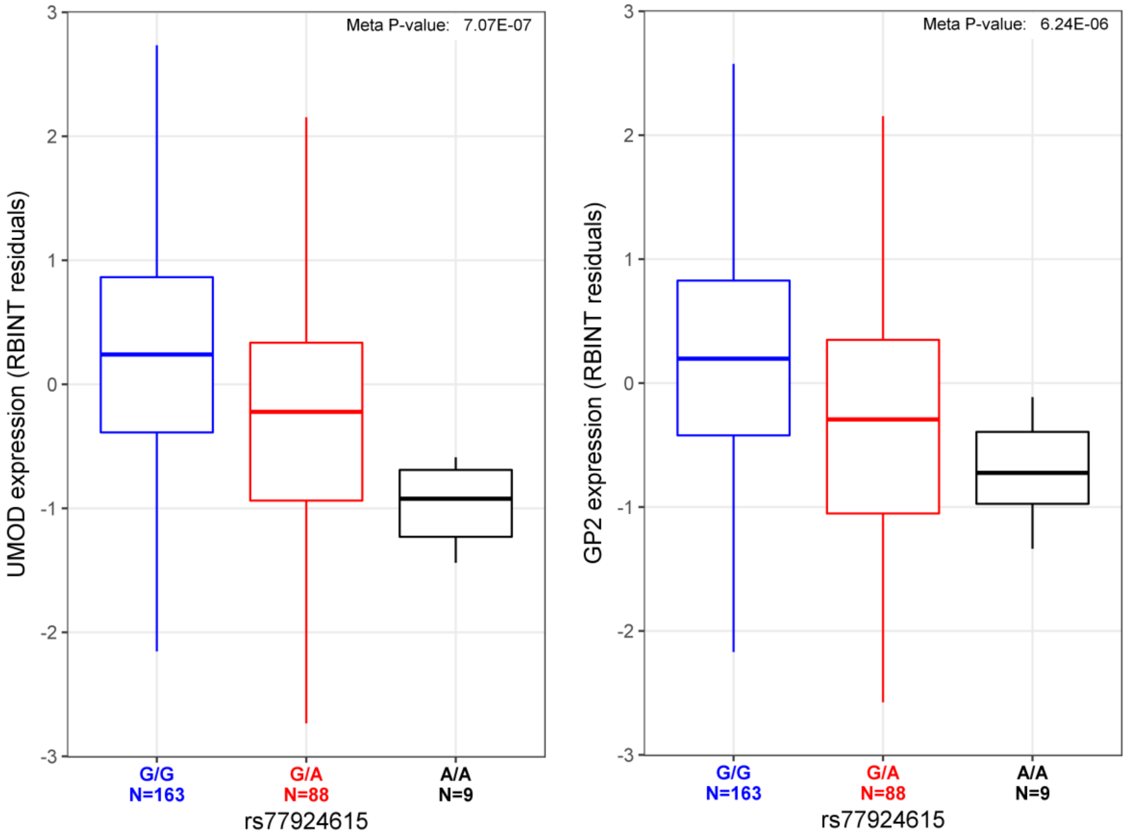

Supplementary Figure 7. Association of index SNV rs77924615 for distinct eGFR signal at the *UMOD-PDILT* locus with expression of *UMOD* and *GP2* in the TRANSLATE Study. The boxplots present median, inter-quartile range, and range of normalised expression for each SNV genotype for the two genes. The eGFR-lowering allele is G. The p-values are obtained from fixed-effects meta-analysis. Source data are provided as a Source Data file.

Supplementary Table 1. Loci attaining genome-wide significant evidence ($p<5 \times 10^{-8}$) of association with eGFR in trans-ethnic meta-analysis of up to 312,468 individuals of diverse ancestry.

Locus	Chr	Interval (bp, b37)	Lead SNV	Position (bp, b37)	*p*-value	Sample size	Component-specific *p*-value			Distinct signals[a]
							COGENT-Kidney	CKDGen	BBJ	
CASP9	1	15,411,947-16,411,947	rs45619934	15,911,947	2.3×10^{-10}	312,468	0.00038	1.7×10^{-8}	0.0078	3
ZNF436	1	23,192,229-24,192,229	rs4525087	23,692,229	5.3×10^{-13}	312,468	0.015	6.3×10^{-5}	7.0×10^{-10}	1
RHOC	1	112,758,681-113,758,681	rs12722725	113,258,681	2.0×10^{-8}	168,224	0.0044	1.5×10^{-7}	N/A	1
ANXA9	1	150,440,625-151,440,625	rs267738	150,940,625	1.7×10^{-10}	309,287	0.092	3.4×10^{-14}	0.024	1
GBAP1	1	154,657,715-155,657,715	rs2070803	155,157,715	4.3×10^{-9}	312,468	0.00049	0.016	1.5×10^{-6}	1
PMF1-BGLAP	1	155,700,671-156,700,671	rs2842870	156,200,671	1.2×10^{-8}	312,468	0.00010	0.046	3.8×10^{-6}	1
CACNA1S	1	200,516,296-201,516,296	rs3850625	201,016,296	2.5×10^{-9}	309,286	2.7×10^{-7}	3.8×10^{-8}	0.23	1
SDCCAG8	1	242,988,186-243,988,186	rs10158537	243,488,186	9.8×10^{-9}	312,468	0.00066	0.0020	5.3×10^{-5}	1
DDX1	2	15,292,518-16,292,518	rs807603	15,792,518	5.2×10^{-14}	312,468	3.2×10^{-5}	1.1×10^{-9}	0.00020	1
NT5C1B-RDH14	2	18,181,365-19,181,365	rs13417750	18,681,365	1.0×10^{-8}	312,468	0.00049	9.6×10^{-7}	0.017	1
GCKR	2	27,230,940-28,230,940	rs1260326	27,730,940	2.0×10^{-35}	309,282	7.1×10^{-15}	6.2×10^{-15}	5.1×10^{-14}	1
C2orf73	2	54,081,356-55,081,356	rs1527649	54,581,356	1.5×10^{-9}	311,225	1.7×10^{-5}	0.0012	0.00014	3
NAT8	2	73,395,765-74,395,765	rs6546869	73,895,765	6.1×10^{-22}	168,810	2.4×10^{-8}	1.9×10^{-17}	N/A	1
PSD4-PAX8	2	113,467,075-114,467,075	rs11123169	113,967,075	8.1×10^{-15}	312,468	2.4×10^{-5}	0.0084	1.4×10^{-12}	1
ORC4	2	148,086,459-149,086,459	rs13026220	148,586,459	3.1×10^{-11}	312,468	0.00093	3.5×10^{-6}	3.2×10^{-5}	1
FAP	2	162,589,866-163,589,866	rs77335736	163,089,866	1.9×10^{-8}	307,068	2.5×10^{-5}	0.18	1.3×10^{-6}	1
LRP2	2	169,702,833-170,702,833	rs3770636	170,202,833	9.1×10^{-24}	280,869	1.4×10^{-8}	0.00037	7.3×10^{-18}	3
HOXD8	2	176,493,583-177,493,583	rs187355703	176,993,583	1.4×10^{-9}	160,758	0.014	1.3×10^{-9}	N/A	1
NFE2L2	2	177,643,371-178,643,371	rs35955110	178,143,371	3.9×10^{-9}	312,468	8.6×10^{-5}	0.0015	0.00011	1
CPS1	2	211,040,507-212,040,507	rs1047891	211,540,507	1.5×10^{-29}	312,468	1.5×10^{-8}	5.9×10^{-16}	7.0×10^{-12}	1
IGFBP5	2	217,174,989-218,174,989	rs7587010	217,674,989	8.6×10^{-14}	312,468	6.7×10^{-5}	1.7×10^{-5}	1.2×10^{-7}	1
XYLB	3	37,998,439-38,998,439	rs36070911	38,498,439	2.3×10^{-11}	312,468	0.00011	9.6×10^{-5}	6.3×10^{-6}	1
SLC15A2	3	121,144,030-122,144,030	rs2250067	121,644,030	2.0×10^{-10}	312,468	0.0093	6.3×10^{-5}	2.3×10^{-6}	1
TFDP2	3	140,640,366-142,216,072	rs1511299	141,716,072	3.0×10^{-17}	311,747	1.5×10^{-7}	6.3×10^{-10}	1.2×10^{-5}	3
ETV5-KNG1	3	184,822,643-186,932,858	rs13081203	185,322,643	1.6×10^{-10}	312,468	0.0032	9.2×10^{-7}	0.00017	3
RGS12	4	2,943,931-3,943,931	rs13108218	3,443,931	1.4×10^{-8}	301,895	0.00062	0.037	2.0×10^{-6}	1
SHROOM3	4	76,901,452-77,901,452	rs142647267	77,401,452	3.9×10^{-59}	312,468	1.3×10^{-13}	3.1×10^{-34}	1.0×10^{-22}	2
PRDM8-FGF5	4	80,682,554-81,682,554	rs12509595	81,182,554	4.7×10^{-16}	312,468	3.3×10^{-5}	0.00039	6.2×10^{-12}	1
NFKB1	4	103,238,972-104,238,972	rs223401	103,738,972	1.6×10^{-8}	310,864	0.013	0.00077	2.0×10^{-5}	1
C1QTNF3-AMACR-RAI14	5	34,009,825-35,009,825	rs10066990	34,509,825	7.4×10^{-11}	305,171	0.0012	0.012	4.7×10^{-9}	1
DAB2	5	38,926,307-39,926,307	rs13179493	39,426,307	1.8×10^{-17}	168,810	6.7×10^{-8}	7.7×10^{-13}	N/A	2
ARL15	5	52,792,390-53,792,390	rs7719168	53,292,390	3.6×10^{-9}	168,810	0.050	3.7×10^{-10}	N/A	2

PIK3R1	5	67,239,274-68,239,274	rs113246091	67,739,274	8.0x10^{-10}	168,398	0.0043	2.9x10^{-9}	N/A	2
RGS14-SLC34A1	5	176,313,404-177,313,404	rs3812036	176,813,404	1.0x10^{-32}	310,355	2.3x10^{-9}	1.9x10^{-17}	3.4x10^{-13}	1
HLA-DRB1-HLA-DQA1	6	32,080,502-33,080,502	rs117463603	32,580,502	1.9x10^{-8}	164,113	0.013	N/A	6.9x10^{-8}	1
C6orf1-RPS10-NUDT3	6	33,871,707-34,871,707	rs6935129	34,371,707	1.5x10^{-10}	311,207	6.5x10^{-5}	0.0046	8.0x10^{-7}	1
VEGFA	6	43,306,609-44,306,609	rs881858	43,806,609	2.6x10^{-22}	312,467	2.3x10^{-9}	3.5x10^{-13}	8.1x10^{-7}	2
AKAP7-ARG1	6	131,382,078-132,382,078	rs9375818	131,882,078	3.8x10^{-10}	312,467	0.0096	0.030	6.0x10^{-10}	1
SLC22A2	6	160,169,081-161,169,081	rs316020	160,669,081	2.8x10^{-25}	307,765	1.2x10^{-7}	2.4x10^{-14}	9.4x10^{-10}	4
UNCX	7	786,567-1,786,567	rs62435145	1,286,567	2.0x10^{-39}	285,548	8.6x10^{-10}	3.8x10^{-8}	6.1x10^{-31}	1
AK125311	7	46,223,510-47,223,510	rs856563	46,723,510	5.1x10^{-10}	309,287	1.5x10^{-7}	6.8x10^{-6}	0.017	1
TMEM60	7	77,052,127-78,052,127	rs848486	77,552,127	1.0x10^{-15}	312,468	5.7x10^{-8}	3.8x10^{-8}	2.6x10^{-5}	1
PRKAG2	7	150,914,329-151,914,329	rs10265221	151,414,329	2.0x10^{-26}	168,810	3.4x10^{-13}	9.2x10^{-18}	N/A	1
SHH	7	155,164,686-156,164,686	rs6971211	155,664,686	6.5x10^{-13}	309,287	0.00014	5.7x10^{-7}	1.2x10^{-5}	2
STC1	8	23,219,571-24,219,571	rs7007761	23,719,571	2.8x10^{-23}	312,468	6.4x10^{-5}	3.0x10^{-14}	4.1x10-10	1
NRG1	8	31,899,662-32,899,662	rs4489283	32,399,662	1.5x10^{-8}	311,632	0.0075	0.0037	5.1x10^{-6}	1
TRIB1	8	125,977,978-126,977,978	rs2001945	126,477,978	1.6x10^{-9}	312,468	0.037	1.6x10^{-6}	0.00012	1
DCAF12	9	33,630,435-34,630,435	rs61237993	34,130,435	4.0x10^{-8}	312,465	0.024	0.00014	0.00016	1
PIP5K1B	9	70,932,174-71,932,174	rs2039424	71,432,174	1.0x10^{-23}	312,465	2.0x10^{-9}	1.9x10^{-17}	1.4x10^{-5}	2
ASTN2	9	118,801,607-119,801,607	rs13283416	119,301,607	3.3x10^{-9}	312,465	0.035	3.4x10^{-7}	0.00070	1
WDR37	10	399,071-1,399,071	rs80282103	899,071	2.0x10^{-18}	312,468	1.2x10^{-5}	8.3x10^{-12}	1.3x10^{-6}	1
MYPN	10	69,465,177-70,465,177	rs7475348	69,965,177	8.6x10^{-19}	312,468	1.3x10^{-5}	2.7x10^{-5}	2.7x10^{-13}	1
CYP26A1	10	94,339,724-95,339,724	rs4418728	94,839,724	1.4x10^{-8}	312,468	0.0010	0.00032	0.00033	1
SUFU	10	104,075,870-105,075,870	rs6892	104,575,870	6.4x10^{-9}	312,468	2.3x10^{-5}	0.054	3.3x10^{-6}	2
FAM53B	10	125,924,137-126,924,137	rs4962691	126,424,137	5.0x10^{-10}	312,468	0.00030	0.00067	1.1x10^{-5}	1
H19-IGF2-KCNQ1	11	1,621,264-3,294,851	rs7482894	2,121,264	2.4x10^{-9}	310,354	0.074	9.3x10^{-5}	2.3x10^{-6}	2
MPPED2	11	30,249,090-31,249,090	rs963837	30,749,090	8.9x10^{-36}	311,223	4.3x10^{-9}	5.5x10^{-16}	9.5x10-18	2
CELF1-PTPMT1	11	46,927,739-48,429,846	rs11039221	47,427,739	8.6x10^{-13}	312,467	1.2x10^{-5}	0.0014	3.2x10^{-8}	1
RNASEH2C	11	65,051,710-66,051,710	rs11604451	65,551,710	5.9x10^{-13}	312,467	0.049	2.0x10^{-9}	2.8x10^{-6}	1
GAB2	11	77,633,077-78,633,077	rs2063724	78,133,077	1.3x10^{-8}	312,467	0.0097	0.011	6.6x10^{-7}	1
SLC6A13	12	0-848,876	rs10774020	348,876	2.0x10^{-9}	201,951	2.5x10^{-5}	N/A	1.4x10^{-6}	1
TSPAN9	12	2,892,351-3,892,351	rs632887	3,392,351	3.7x10^{-9}	201,951	0.0024	N/A	5.4x10^{-8}	1
KRR1-PHLDA1	12	75,771,183-76,771,183	rs1275609	76,271,183	1.2x10^{-8}	201,004	0.095	N/A	1.5x10^{-9}	1
CUX2	12	111,218,231-112,218,231	rs79105258	111,718,231	5.7x10^{-15}	156,434	0.87	N/A	5.3x10^{-18}	1
DGKH	13	42,257,213-43,257,213	rs34445998	42,757,213	2.8x10^{-8}	312,467	0.10	0.053	3.8x10^{-9}	1
DACH1	13	71,845,505-72,845,505	rs584480	72,345,505	1.6x10^{-19}	312,467	0.0029	8.0x10^{-11}	1.0x10^{-10}	1
RASGRP1	15	38,773,575-39,773,575	rs9920185	39,273,575	1.0x10^{-8}	312,468	0.00019	9.6x10^{-5}	0.0019	1
GATM	15	45,212,339-46,212,339	rs2486288	45,712,339	1.4x10^{-33}	312,468	5.4x10^{-12}	1.6x10^{-41}	0.011	1
WDR72	15	53,415,766-54,415,766	rs62005941	53,915,766	2.9x10^{-36}	312,468	7.8x10^{-7}	5.4x10^{-9}	1.3x10^{-29}	2

UBE2Q2	15	75,739,020-76,739,020	rs11636251	76,239,020	$2.9x10^{-21}$	312,468	$1.6x10^{-7}$	$3.0x10^{-10}$	$4.6x10^{-9}$	2
CEMIP	15	80,665,842-81,665,842	rs62035088	81,165,842	$8.2x10^{-9}$	307,059	0.00032	0.063	$5.5x10^{-7}$	1
IGF1R	15	98,749,029-99,749,029	rs11858316	99,249,029	$2.3x10^{-9}$	312,468	0.016	0.00032	$4.1x10^{-6}$	1
UMOD-PDILT	16	19,892,332-20,892,332	rs77924615	20,392,332	$2.9x10^{-83}$	312,468	$5.9x10^{-13}$	$3.0x10^{-36}$	$2.2x10^{-47}$	4
SALL1	16	51,254,991-52,254,991	rs12935539	51,754,991	$2.2x10^{-14}$	312,468	$7.1x10^{-5}$	$3.1x10^{-6}$	$1.4x10^{-7}$	2
SLC7A6	16	67,797,589-68,797,589	rs9888796	68,297,589	$3.2x10^{-10}$	312,468	0.00024	$4.9x10^{-8}$	0.00078	1
NFAT5	16	69,122,104-70,122,104	rs11641050	69,622,104	$2.6x10^{-8}$	312,468	0.029	0.0020	$4.9x10^{-6}$	2
DPEP1	16	89,200,747-90,200,747	rs2460449	89,700,747	$4.2x10^{-9}$	166,697	$5.9x10^{-6}$	$1.3x10^{-5}$	N/A	1
SLC47A1	17	18,932,393-19,932,393	rs11871125	19,432,393	$4.8x10^{-11}$	304,944	0.11	$9.2x10^{-12}$	0.00089	1
CDK12-MED1	17	37,216,771-38,216,771	rs4795384	37,716,771	$1.6x10^{-24}$	312,466	$2.9x10^{-5}$	$5.4x10^{-15}$	$1.8x10^{-10}$	1
BCAS3	17	58,446,691-59,956,589	rs9895661	59,456,589	$3.4x10^{-47}$	312,466	$1.4x10^{-10}$	$6.9x10^{-22}$	$3.2x10^{-23}$	3
EPB41L3	18	5,085,158-6,085,158	rs1719934	5,585,158	$2.0x10^{-11}$	312,467	$2.8x10^{-5}$	$6.7x10^{-8}$	0.0025	1
PCAT18-AQP4	18	23,893,213-24,893,213	rs16942751	24,393,213	$2.3x10^{-11}$	312,467	0.010	$4.9x10^{-5}$	$2.0x10^{-7}$	2
SMAD7	18	45,960,903-46,960,903	rs2337106	46,460,903	$6.0x10^{-9}$	307,058	$7.2x10^{-6}$	0.24	$3.4x10^{-7}$	1
RNF152	18	58,879,074-59,879,074	rs896642	59,379,074	$4.6x10^{-9}$	312,467	$3.4x10^{-5}$	$2.4x10^{-7}$	0.052	1
NFATC1	18	76,656,537-77,656,537	rs8096658	77,156,537	$1.7x10^{-28}$	265,020	$7.2x10^{-6}$	$1.3x10^{-9}$	$4.6x10^{-19}$	1
JUND-LSM4	19	17,908,519-18,908,519	rs8108623	18,408,519	$4.4x10^{-8}$	309,634	0.00064	$3.1x10^{-5}$	0.0071	1
SLC7A9	19	32,860,369-33,860,369	rs7252778	33,360,369	$3.4x10^{-17}$	312,468	0.00041	$2.6x10^{-12}$	$2.3x10^{-6}$	1
TP53INP2	20	33,029,766-34,029,766	rs2273684	33,529,766	$1.4x10^{-11}$	309,282	0.043	$8.0x10^{-11}$	0.00038	1
BCAS1	20	52,232,362-53,232,362	rs17216707	52,732,362	$4.6x10^{-16}$	312,466	$1.9x10^{-7}$	$8.6x10^{-14}$	0.0056	1
ARFRP1	20	61,836,334-62,836,334	rs1758206	62,336,334	$2.4x10^{-8}$	163,534	0.00079	$1.3x10^{-6}$	N/A	1
NRIP1	21	16,076,783-17,076,783	rs2823139	16,576,783	$3.7x10^{-9}$	311,637	0.0011	$1.1x10^{-5}$	0.0012	1
ATP50	21	34,856,706-35,856,706	rs2834317	35,356,706	$9.5x10^{-10}$	312,468	$2.6x10^{-5}$	$3.9x10^{-7}$	0.017	1
MKL1	22	40,380,213-41,380,213	rs17001977	40,880,213	$6.7x10^{-15}$	311,747	$1.2x10^{-5}$	0.00059	$2.7x10^{-10}$	2

Chr: chromosome.

[a]Number of distinct association signals attaining locus-wide significance ($p<10^{-5}$) identified through approximate conditional analyses.

Supplementary Table 2. Association summary statistics for index SNVs for distinct eGFR signals attaining locus-wide significance ($p<10^{-5}$) in trans-ethnic meta-analysis of up to 312,468 individuals of diverse ancestry.

Locus	Index SNV	Chr	Position (bp, b37)	Alleles[a]		EAF	Fixed-effects meta-analysis[b]				Heterogeneity *p*-value[c,d]	
				Effect	Other		*p*-value	*N*	Beta	SE	Ancestral	Residual
CASP9	rs10927723	1	15,538,534	G	C	0.341	$3.3x10^{-6}$	312,468	-0.152	0.102	0.079	0.81
	rs6664388	1	15,908,242	T	C	0.407	$2.7x10^{-10}$	312,468	-0.408	0.102	0.53	0.63
	rs1763632	1	16,312,138	T	C	0.203	$2.4x10^{-7}$	302,697	-0.157	0.120	0.67	0.94
ZNF436	rs4525087	1	23,692,229	C	A	0.650	$5.3x10^{-13}$	312,468	-0.283	0.094	0.43	0.22
RHOC	rs12722725	1	113,258,681	C	T	0.108	$2.0x10^{-8}$	168,224	-0.500	0.189	0.94	0.52
ANXA9	rs267738	1	150,940,625	T	G	0.886	$1.7x10^{-10}$	309,287	-0.226	0.136	0.83	0.14
GBAP1	rs2070803	1	155,157,715	A	G	0.386	$4.3x10^{-9}$	312,468	-0.292	0.095	0.034	0.0011
PMF1-BGLAP	rs2842870	1	156,200,671	T	C	0.632	$1.2x10^{-8}$	312,468	-0.361	0.094	0.15	0.036
CACNA1S	rs3850625	1	201,016,296	G	A	0.926	$2.5x10^{-9}$	309,286	-0.665	0.166	0.028	0.53
SDCCAG8	rs10158537	1	243,488,186	G	C	0.243	$9.8x10^{-9}$	312,468	-0.361	0.105	0.79	0.29
DDX1	rs807603	2	15,792,518	T	C	0.438	$5.2x10^{-14}$	312,468	-0.383	0.096	0.083	0.00059
NT5C1B-RDH14	rs13417750	2	18,681,365	A	G	0.189	$1.0x10^{-8}$	312,468	-0.439	0.108	0.71	0.0074
GCKR	rs1260326	2	27,730,940	C	T	0.534	$2.0x10^{-35}$	309,282	-0.675	0.096	0.0012	0.38
C2orf73	rs10181201	2	54,799,174	G	A	0.078	$7.4x10^{-8}$	310,791	-0.129	0.132	0.031	0.38
	rs7586066	2	54,810,492	A	G	0.848	$9.7x10^{-13}$	312,468	-0.102	0.067	0.73	0.97
	rs17046036	2	54,855,454	A	G	0.122	$1.9x10^{-15}$	312,468	-0.160	0.071	0.40	0.83
NAT8	rs6546869	2	73,895,765	G	A	0.768	$6.1x10^{-22}$	168,810	-0.566	0.113	0.29	0.37
PSD4-PAX8	rs11123169	2	113,967,075	C	T	0.302	$8.1x10^{-15}$	312,468	-0.438	0.096	0.95	0.33
ORC4	rs13026220	2	148,586,459	G	A	0.366	$3.1x10^{-11}$	312,468	-0.265	0.095	0.24	0.018
FAP	rs77335736	2	163,089,866	C	T	0.913	$1.9x10^{-8}$	307,068	-0.252	0.141	0.81	0.13
LRP2	rs35472707	2	169,995,581	T	C	0.048	$1.1x10^{-6}$	163,854	-0.496	0.268	0.38	0.30
	rs4140872	2	170,026,596	T	C	0.621	0.00078	311,227	-0.250	0.116	0.25	0.44
	rs60641214	2	170,199,292	A	T	0.758	$5.6x10^{-8}$	312,468	-0.368	0.126	0.81	0.87
HOXD8	rs187355703	2	176,993,583	G	C	0.016	$1.4x10^{-9}$	160,758	-1.098	0.403	0.075	0.012
NFE2L2	rs35955110	2	178,143,371	C	T	0.435	$3.9x10^{-9}$	312,468	-0.353	0.099	0.16	0.32
CPS1	rs1047891	2	211,540,507	A	C	0.237	$1.5x10^{-29}$	312,468	-0.610	0.105	0.99	0.23
IGFBP5	rs7587010	2	217,674,989	T	G	0.545	$8.6x10^{-14}$	312,468	-0.337	0.089	0.53	0.0012
XYLB	rs36070911	3	38,498,439	G	A	0.528	$2.3x10^{-11}$	312,468	-0.296	0.091	0.15	0.057
SLC15A2	rs2250067	3	121,644,030	T	C	0.539	$2.0x10^{-10}$	312,468	-0.205	0.100	0.099	0.039
TFDP2	rs6440003	3	141,094,209	A	G	0.408	$3.8x10^{-10}$	312,468	-0.406	0.096	0.14	0.55
	rs1511299	3	141,716,072	T	C	0.739	$8.8x10^{-15}$	311,747	-0.560	0.116	0.51	0.32
	rs147877018	3	141,813,349	A	G	0.057	$1.2x10^{-8}$	308,051	-0.816	0.214	0.65	0.49

ETV5-KNG1	rs13081203	3	185,322,643	A	G	0.343	8.8x10^{-11}	312,468	-0.241	0.100	0.94	0.44
	rs6809651	3	185,814,642	A	G	0.079	2.6x10^{-8}	310,505	-0.299	0.155	0.12	0.44
	rs11927941	3	186,433,046	G	A	0.312	6.2x10^{-10}	312,468	-0.206	0.101	0.0064	0.34
RGS12	rs13108218	4	3,443,931	G	A	0.526	1.4x10^{-8}	301,895	-0.389	0.096	0.31	0.31
SHROOM3	rs111449836	4	77,401,452	T	C	0.320	4.6x10^{-30}	312,468	-0.501	0.083	0.48	0.086
	rs13106227	4	77,418,681	A	G	0.572	1.6x10^{-6}	309,694	-0.146	0.080	0.87	0.59
PRDM8-FGF5	rs12509595	4	81,182,554	T	C	0.721	4.7x10^{-16}	312,468	-0.398	0.105	0.15	**0.00023**
NFKB1	rs223401	4	103,738,972	T	C	0.642	1.6x10^{-8}	310,864	-0.100	0.098	0.43	0.80
C1QTNF3-AMACR-RAI14	rs10066990	5	34,509,825	A	G	0.300	7.4x10^{-11}	305,171	-0.406	0.101	0.057	0.37
DAB2	rs12153248	5	39,400,305	T	C	0.313	9.0x10^{-10}	312,468	-0.266	0.083	0.055	0.55
	rs4016485	5	39,871,447	G	A	0.853	2.9x10^{-6}	312,468	-0.330	0.139	0.79	0.98
ARL15	rs114118094	5	53,285,762	T	A	0.064	3.8x10^{-6}	164,702	-0.510	0.246	0.69	0.81
	rs7719168	5	53,292,390	A	C	0.897	1.3x10^{-8}	168,810	-0.168	0.179	0.55	0.016
PIK3R1	rs113246091	5	67,739,274	A	G	0.077	6.6x10^{-10}	168,398	-0.498	0.190	0.051	0.10
	rs78660602	5	68,043,894	A	G	0.918	1.1x10^{-7}	309,719	-0.631	0.175	0.90	0.72
RGS14-SLC34A1	rs3812036	5	176,813,404	T	C	0.248	1.0x10^{-32}	310,355	-0.801	0.112	0.045	0.29
HLA-DRB1-HLA-DQA1	rs9368730	6	32,580,502	G	A	0.876	1.9x10^{-8}	164,113	-1.403	0.338	0.23	0.0030
C6orf1-RPS10-NUDT3	rs6935129	6	34,371,707	A	G	0.303	1.5x10^{-10}	311,207	-0.233	0.128	0.027	**1.0x10^{-7}**
VEGFA	rs1214761	6	43,354,431	G	A	0.630	6.3x10^{-7}	312,467	-0.099	0.093	0.53	0.33
	rs881858	6	43,806,609	A	G	0.775	4.5x10^{-21}	312,467	-0.617	0.109	0.84	**0.00012**
AKAP7-ARG1	rs9375818	6	131,882,078	A	G	0.284	3.8x10^{-10}	312,467	-0.365	0.101	0.47	0.48
SLC22A2	rs920811	6	160,496,243	G	T	0.526	6.3x10^{-7}	312,467	-0.357	0.102	0.75	0.15
	rs73025532	6	160,537,308	T	C	0.088	2.0x10^{-9}	165,113	-0.530	0.200	0.92	0.37
	rs515140	6	160,648,267	T	G	0.202	3.6x10^{-22}	308,351	-0.518	0.097	0.34	0.13
	rs316020	6	160,669,081	G	A	0.919	8.5x10^{-17}	307,765	-0.737	0.169	0.28	0.0040
UNCX	rs62435145	7	1,286,567	T	G	0.480	2.0x10^{-39}	285,548	-0.594	0.105	0.065	0.22
AK125311	rs856563	7	46,723,510	C	T	0.750	5.1x10^{-10}	309,287	-0.455	0.094	0.41	0.24
TMEM60	rs848486	7	77,552,127	G	A	0.340	1.0x10^{-15}	312,468	-0.501	0.094	0.39	0.29
PRKAG2	rs10265221	7	151,414,329	C	T	0.271	2.0x10^{-26}	168,810	-0.847	0.124	0.32	0.80
SHH	rs6971211	7	155,664,686	T	C	0.417	1.4x10^{-12}	309,287	-0.347	0.090	0.61	0.093
	rs73176866	7	156,119,361	C	T	0.754	3.9x10^{-6}	312,468	-0.256	0.118	0.64	0.66
STC1	rs7007761	8	23,719,571	T	C	0.299	2.8x10^{-23}	312,468	-0.484	0.097	0.077	0.98
NRG1	rs4489283	8	32,399,662	T	C	0.296	1.5x10^{-8}	311,632	-0.325	0.094	0.074	0.43
TRIB1	rs2001945	8	126,477,978	C	G	0.546	1.6x10^{-9}	312,468	-0.264	0.091	0.88	0.78
DCAF12	rs61237993	9	34,130,435	G	A	0.666	4.0x10^{-8}	312,465	-0.345	0.122	0.027	0.55
PIP5K1B	rs10120859	9	71,156,295	T	C	0.816	4.8x10^{-10}	312,465	-0.287	0.130	0.39	0.89
	rs10746942	9	71,434,465	G	A	0.322	1.1x10^{-26}	312,465	-0.596	0.096	0.58	0.53

ASTN2	rs13283416	9	119,301,607	G	T	0.498	3.3x10^{-9}	312,465	-0.232	0.093	0.55	0.066
WDR37	rs80282103	10	899,071	T	A	0.080	2.0x10^{-18}	312,468	-0.615	0.157	0.64	0.038
MYPN	rs7475348	10	69,965,177	C	T	0.607	8.6x10^{-19}	312,468	-0.366	0.095	0.91	0.20
CYP26A1	rs4418728	10	94,839,724	T	G	0.539	1.4x10^{-8}	312,468	-0.345	0.092	0.94	0.66
SUFU	rs75174967	10	104,277,093	A	G	0.134	1.5x10^{-6}	170,174	-0.356	0.249	0.93	0.68
	rs6892	10	104,575,870	A	G	0.775	1.7x10^{-5}	312,468	-0.311	0.131	0.081	0.12
FAM53B	rs4962691	10	126,424,137	T	C	0.571	5.0x10^{-10}	312,468	-0.291	0.093	0.039	0.63
H19-IGF2-KCNQ1	rs7482894	11	2,121,264	T	C	0.568	2.1x10^{-10}	310,354	-0.271	0.100	0.62	0.31
	rs231889	11	2,777,192	C	T	0.107	2.1x10^{-10}	311,638	-0.416	0.137	0.74	0.30
MPPED2	rs7930738	11	30,605,859	C	G	0.816	4.7x10^{-7}	311,746	-0.431	0.132	0.21	0.43
	rs963837	11	30,749,090	T	C	0.600	9.3x10^{-37}	311,223	-0.532	0.097	0.57	**5.0x10^{-9}**
CELF1-PTPMT1	rs11039221	11	47,427,739	T	C	0.307	8.6x10^{-13}	312,467	-0.441	0.100	0.92	0.22
RNASEH2C	rs11604451	11	65,551,710	T	C	0.249	5.9x10^{-13}	312,467	-0.248	0.107	0.050	0.21
GAB2	rs2063724	11	78,133,077	T	C	0.659	1.3x10^{-8}	312,467	-0.252	0.101	0.79	0.24
SLC6A13	rs10774020	12	348,876	C	T	0.378	2.0x10^{-9}	201,951	-0.468	0.094	0.097	0.17
TSPAN9	rs632887	12	3,392,351	G	A	0.331	3.7x10^{-9}	201,951	-0.317	0.094	0.34	0.85
KRR1-PHLDA1	rs1275609	12	76,271,183	G	A	0.477	1.2x10^{-8}	201,004	-0.258	0.100	0.073	0.50
CUX2	rs79105258	12	111,718,231	A	C	0.230	5.7x10^{-15}	156,434	-1.263	0.314	0.34	0.24
DGKH	rs34445998	13	42,757,213	C	T	0.733	2.8x10^{-8}	312,467	-0.318	0.120	0.93	0.36
DACH1	rs584480	13	72,345,505	C	T	0.369	1.6x10^{-19}	312,467	-0.298	0.094	0.57	0.49
RASGRP1	rs9920185	15	39,273,575	C	A	0.649	1.0x10^{-8}	312,468	-0.332	0.094	0.017	0.017
GATM	rs2486288	15	45,712,339	C	T	0.684	1.4x10^{-33}	312,468	-0.628	0.102	0.037	0.14
WDR72	rs7169329	15	53,902,626	G	C	0.592	7.1x10^{-7}	312,468	-0.130	0.087	0.36	0.26
	rs35255404	15	53,919,226	G	A	0.705	8.3x10^{-42}	312,468	-0.603	0.097	0.31	0.00073
UBE2Q2	rs10851885	15	76,304,503	G	A	0.220	4.6x10^{-12}	165,629	-0.419	0.137	0.0060	0.13
	rs76614553	15	76,309,608	C	T	0.980	9.8x10^{-9}	161,479	-1.074	0.315	0.97	0.45
CEMIP	rs62035088	15	81,165,842	G	A	0.851	8.2x10^{-9}	307,059	-0.272	0.122	0.73	0.036
IGF1R	rs11858316	15	99,249,029	C	T	0.462	2.3x10^{-9}	312,468	-0.107	0.092	0.31	0.067
UMOD-PDILT	rs13329952	16	20,366,507	T	C	0.855	4.1x10^{-20}	312,468	-0.339	0.108	0.35	0.063
	rs4494548	16	20,371,588	A	G	0.074	2.8x10^{-9}	312,468	-0.362	0.172	1.0	0.070
	rs77924615	16	20,392,332	G	A	0.792	1.5x10^{-54}	312,468	-0.657	0.108	0.17	0.0078
	rs757002	16	20,496,273	A	G	0.836	7.0x10^{-6}	312,468	-0.208	0.141	0.32	0.63
SALL1	rs12935539	16	51,754,991	C	T	0.262	1.9x10^{-18}	312,468	-0.465	0.101	0.57	0.39
	rs7184096	16	51,761,696	T	G	0.139	2.4x10^{-7}	312,468	-0.277	0.124	0.59	0.030
SLC7A6	rs9888796	16	68,297,589	T	C	0.186	3.2x10^{-10}	312,468	-0.324	0.114	0.023	0.31
NFAT5	rs11641050	16	69,622,104	C	T	0.697	1.3x10^{-11}	312,468	-0.372	0.092	0.86	0.84
	rs8052428	16	69,913,996	T	C	0.369	3.4x10^{-6}	312,468	-0.346	0.089	0.21	0.88

DPEP1	rs2460449	16	89,700,747	A	G	0.431	4.2x10^{-9}	166,697	-0.380	0.112	0.035	0.82
SLC47A1	rs11871125	17	19,432,393	T	C	0.276	4.8x10^{-11}	304,944	-0.226	0.110	0.39	0.039
CDK12-MED1	rs4795384	17	37,716,771	G	C	0.763	1.6x10^{-24}	312,466	-0.395	0.101	0.81	0.15
BCAS3	rs8080123	17	59,242,914	G	T	0.792	2.2x10^{-26}	312,466	-0.630	0.110	0.37	0.31
	rs9895661	17	59,456,589	C	T	0.373	8.9x10^{-28}	312,466	-0.485	0.095	0.28	0.060
	rs887258	17	59,479,580	C	G	0.278	2.7x10^{-13}	312,466	-0.361	0.094	0.84	0.45
EPB41L3	rs1719934	18	5,585,158	G	A	0.329	2.0x10^{-11}	312,467	-0.344	0.094	0.81	0.084
PCAT18-AQP4	rs16942751	18	24,393,213	A	C	0.193	4.7x10^{-12}	312,467	-0.439	0.129	0.97	0.72
	rs162005	18	24,447,786	A	G	0.764	6.1x10^{-7}	305,817	-0.027	0.110	0.48	0.55
SMAD7	rs2337106	18	46,460,903	C	G	0.453	6.0x10^{-9}	307,058	-0.451	0.094	0.14	0.51
RNF152	rs896642	18	59,379,074	C	T	0.725	4.6x10^{-9}	312,467	-0.458	0.106	0.27	0.010
NFATC1	rs8096658	18	77,156,537	G	C	0.380	1.7x10^{-28}	265,020	-0.701	0.117	0.52	0.69
JUND-LSM4	rs8108623	19	18,408,519	A	C	0.695	4.4x10^{-8}	309,634	-0.390	0.108	0.10	0.41
SLC7A9	rs7252778	19	33,360,369	A	C	0.461	3.4x10^{-17}	312,468	-0.320	0.092	0.051	0.14
TP53INP2	rs2273684	20	33,529,766	G	T	0.336	1.4x10^{-11}	309,282	-0.221	0.098	0.064	0.078
BCAS1	rs17216707	20	52,732,362	T	C	0.839	4.6x10^{-16}	312,466	-0.527	0.115	0.82	0.094
ARFRP1	rs1758206	20	62,336,334	T	C	0.082	2.4x10^{-8}	163,534	-0.546	0.193	0.044	0.21
NRIP1	rs2823139	21	16,576,783	A	G	0.293	3.7x10^{-9}	311,637	-0.197	0.093	0.67	0.49
ATP50	rs2834317	21	35,356,706	A	G	0.108	9.5x10^{-10}	312,468	-0.475	0.126	0.43	0.65
MKL1	rs17001943	22	40,862,613	A	G	0.817	1.8x10^{-16}	312,468	-0.865	0.155	0.35	0.59
	rs80533	22	41,085,969	G	A	0.847	1.2x10^{-6}	310,506	-0.055	0.114	0.64	0.81

Chr: chromosome. EAF: effect allele frequency. SE: standard error.
[a]Effect allele is aligned to be eGFR decreasing allele.
[b]Beta/SE are obtained from fixed-effects meta-analysis, with inverse variance weighting of allelic effect sizes, of up to 81,829 individuals of diverse ancestry from the COGENT-Kidney Consortium, and represent absolute decrease in eGFR (ml/min per 1.73m^2) per effect allele.
[c]Heterogeneity p-values are obtained from trans-ethnic meta-regression (MR-MEGA) of up to 81,829 individuals of diverse ancestry from the COGENT-Kidney Consortium.
[c]Significant heterogeneity after Bonferroni correction for number of signals are highlighted in bold.

Supplementary Table 3. Ethnic-specific association summary statistics for distinct eGFR signals in up to 81,829 individuals from the COGENT-Kidney Consortium.

Index SNV	Alleles[a]		African American (up to 8,224 individuals)[b]				East Asian (up to 23,536 individuals)[b]				European (up to 23,553 individuals)[b]				Hispanic (up to 26,516 individuals)[b]			
	Effect	Other	EAF	Beta	SE	*p*-value	EAF	Beta	SE	*p*-value	EAF	Beta	SE	*p*-value	EAF	Beta	SE	*p*-value
rs10927723	G	C	0.218	0.329	0.363	0.36	0.327	-0.530	0.273	0.052	0.364	0.058	0.200	0.77	0.263	-0.227	0.141	0.11
rs6664388	T	C	0.263	-0.753	0.345	0.029	0.550	-0.679	0.275	0.014	0.311	-0.507	0.215	0.018	0.279	-0.243	0.138	0.079
rs1763632	T	C	0.037	-1.243	0.798	0.12	0.295	-0.286	0.286	0.32	0.102	-0.104	0.323	0.75	0.273	-0.098	0.147	0.51
rs4525087	C	A	0.265	-0.139	0.353	0.69	0.561	-0.740	0.259	0.0043	0.742	-0.386	0.180	0.031	0.632	-0.134	0.130	0.30
rs12722725	C	T	0.043	-0.448	0.999	0.65	N/A	N/A	N/A	N/A	0.127	-0.513	0.250	0.041	0.055	-0.488	0.301	0.10
rs267738	T	G	0.949	0.163	0.698	0.82	0.956	-0.200	0.631	0.75	0.784	-0.280	0.188	0.14	0.897	-0.196	0.218	0.37
rs2070803	A	G	0.575	-0.907	0.332	0.0062	0.182	-0.762	0.333	0.022	0.571	-0.222	0.157	0.16	0.422	-0.157	0.139	0.26
rs2842870	T	C	0.489	-0.619	0.329	0.060	0.623	-0.914	0.264	0.00054	0.636	-0.302	0.161	0.061	0.661	-0.203	0.140	0.15
rs3850625	G	A	0.974	-2.175	0.950	0.022	0.966	0.660	0.707	0.35	0.889	-0.855	0.265	0.0012	0.909	-0.572	0.231	0.013
rs10158537	G	C	0.238	-0.368	0.349	0.29	0.176	-0.497	0.339	0.14	0.302	-0.518	0.175	0.0031	0.218	-0.206	0.156	0.19
rs807603	T	C	0.263	-0.637	0.346	0.066	0.204	-0.215	0.322	0.50	0.641	-0.594	0.163	0.00027	0.569	-0.224	0.137	0.10
rs13417750	A	G	0.345	-0.521	0.313	0.097	0.020	-3.066	0.931	0.00099	0.347	-0.316	0.164	0.053	0.201	-0.459	0.164	0.0050
rs1260326	C	T	0.838	-1.123	0.422	0.0078	0.442	-0.637	0.262	0.015	0.601	-0.830	0.158	1.6x10^{-7}	0.677	-0.510	0.142	0.00034
rs10181201	G	A	0.501	0.417	0.302	0.17	0.057	-0.550	0.306	0.072	N/A	N/A	N/A	N/A	0.192	-0.169	0.167	0.31
rs7586066	A	G	0.861	0.037	0.185	0.84	0.866	-0.189	0.132	0.15	N/A	N/A	N/A	N/A	0.903	-0.095	0.086	0.27
rs17046036	A	G	0.134	-0.056	0.187	0.76	0.080	-0.381	0.166	0.021	N/A	N/A	N/A	N/A	0.091	-0.122	0.087	0.16
rs6546869	G	A	0.490	-0.887	0.306	0.0038	N/A	N/A	N/A	N/A	0.780	-1.036	0.197	1.5x10^{-7}	0.747	-0.202	0.153	0.19
rs11123169	C	T	0.369	-1.045	0.307	0.00065	0.269	-0.824	0.292	0.0048	0.342	-0.364	0.168	0.031	0.315	-0.274	0.140	0.050
rs13026220	G	A	0.180	-0.063	0.396	0.87	0.431	-0.369	0.264	0.16	0.305	-0.370	0.171	0.031	0.382	-0.198	0.133	0.14
rs77335736	C	T	0.987	-3.412	1.590	0.032	0.880	-0.700	0.495	0.16	0.980	-2.050	0.710	0.0039	0.721	-0.100	0.151	0.51
rs35472707	T	C	0.035	-0.900	0.911	0.32	N/A	N/A	N/A	N/A	0.049	-0.792	0.369	0.032	0.036	-0.001	0.432	1.0
rs4140872	T	C	0.892	-0.815	0.484	0.092	N/A	N/A	N/A	N/A	0.752	-0.406	0.177	0.021	0.807	-0.054	0.162	0.74
rs60641214	A	T	0.657	-0.148	0.321	0.64	N/A	N/A	N/A	N/A	0.804	-0.404	0.203	0.047	0.850	-0.413	0.187	0.027
rs187355703	G	C	0.012	-0.872	1.761	0.62	N/A	N/A	N/A	N/A	0.031	-1.215	0.461	0.0084	0.014	-0.681	0.938	0.47
rs35955110	C	T	0.195	-1.148	0.378	0.0024	0.572	-0.548	0.264	0.038	0.313	-0.324	0.173	0.060	0.432	-0.196	0.145	0.18
rs1047891	A	C	0.355	-0.561	0.338	0.096	0.153	-1.445	0.381	0.00015	0.322	-0.688	0.175	8.1x10^{-5}	0.309	-0.427	0.153	0.0052
rs7587010	T	G	0.442	-0.368	0.305	0.23	0.512	-0.663	0.257	0.0099	0.580	-0.541	0.156	0.00053	0.619	-0.103	0.131	0.43
rs36070911	G	A	0.355	-1.054	0.314	0.00079	0.468	-0.310	0.258	0.23	0.619	-0.381	0.160	0.018	0.534	-0.099	0.133	0.45
rs2250067	T	C	0.462	-0.373	0.308	0.23	0.710	-0.377	0.294	0.20	0.429	-0.092	0.161	0.57	0.275	-0.221	0.158	0.16
rs6440003	A	G	0.794	-1.089	0.374	0.0036	0.359	-0.776	0.270	0.0041	0.443	-0.357	0.155	0.022	0.453	-0.236	0.147	0.11
rs1511299	T	C	0.906	-1.030	0.520	0.048	0.715	-0.624	0.285	0.028	0.741	-0.629	0.175	0.00032	0.832	-0.374	0.197	0.057
rs147877018	A	G	0.031	-0.021	1.113	0.99	0.036	-0.320	0.689	0.64	0.079	-0.930	0.297	0.0018	0.043	-0.866	0.363	0.017
rs13081203	A	G	0.419	-0.066	0.318	0.84	0.383	-0.410	0.265	0.12	0.323	-0.364	0.173	0.035	0.318	-0.128	0.153	0.40
rs6809651	A	G	0.206	-0.571	0.386	0.14	0.016	-2.021	1.019	0.047	0.132	-0.026	0.231	0.91	0.092	-0.408	0.258	0.11
rs11927941	G	A	0.463	-0.656	0.302	0.030	0.296	-0.922	0.279	0.00095	0.316	0.073	0.168	0.66	0.286	-0.099	0.160	0.53
rs13108218	G	A	0.516	-0.659	0.341	0.053	0.447	-0.686	0.261	0.0086	0.600	-0.313	0.177	0.077	0.540	-0.309	0.137	0.024
rs111449836	T	C	0.149	-0.240	0.409	0.56	0.221	-1.110	0.269	3.7x10^{-5}	0.448	-0.562	0.129	1.3x10^{-5}	0.354	-0.342	0.123	0.0054
rs13106227	A	G	0.446	-0.080	0.281	0.78	0.503	-0.116	0.223	0.60	0.644	-0.220	0.134	0.10	0.616	-0.106	0.121	0.38
rs12509595	T	C	0.901	-1.120	0.551	0.042	0.691	-0.470	0.299	0.12	0.694	-0.661	0.177	0.00019	0.753	-0.134	0.151	0.38
rs223401	T	C	0.572	0.035	0.304	0.91	N/A	N/A	N/A	N/A	0.665	-0.353	0.167	0.035	0.507	0.035	0.133	0.79

rs10066990	A	G	0.127	-0.673	0.458	0.14	0.238	-1.054	0.303	0.00051	0.381	-0.196	0.172	0.25	0.278	-0.381	0.142	0.0074
rs12153248	T	C	0.264	-0.544	0.314	0.083	N/A	N/A	N/A	N/A	0.416	-0.397	0.129	0.0021	0.372	-0.125	0.115	0.28
rs4016485	G	A	0.935	0.291	0.605	0.63	0.843	-0.337	0.352	0.34	0.853	-0.340	0.219	0.12	0.894	-0.403	0.223	0.072
rs114118094	T	A	0.017	-1.356	1.198	0.26	N/A	N/A	N/A	N/A	0.075	-0.550	0.306	0.073	0.030	-0.316	0.438	0.47
rs7719168	A	C	0.957	0.984	0.760	0.20	N/A	N/A	N/A	N/A	0.888	-0.269	0.251	0.28	0.935	-0.197	0.270	0.47
rs113246091	A	G	0.089	0.331	0.543	0.54	0.010	-3.272	1.296	0.012	0.097	-0.370	0.268	0.17	0.061	-0.796	0.318	0.012
rs78660602	A	G	0.878	-0.718	0.454	0.11	0.920	-0.696	0.478	0.14	0.914	-0.575	0.280	0.040	0.941	-0.631	0.306	0.040
rs3812036	T	C	0.083	-0.477	0.547	0.38	0.251	-1.722	0.301	1.1x10^{-8}	0.256	-0.847	0.186	4.9x10^{-6}	0.199	-0.514	0.166	0.0019
rs9368730	G	A	N/A	N/A	N/A	N/A	0.845	-1.267	0.358	0.00040	0.985	-2.118	1.443	0.14	0.992	-2.947	1.461	0.044
rs6935129	A	G	0.075	-0.040	0.599	0.95	0.531	-0.076	0.265	0.77	0.045	-1.192	0.399	0.0028	0.305	-0.147	0.162	0.36
rs1214761	G	A	0.345	-0.247	0.316	0.43	0.656	0.091	0.271	0.74	0.666	-0.219	0.164	0.18	0.556	-0.038	0.134	0.78
rs881858	A	G	0.412	-0.323	0.318	0.31	0.868	-0.809	0.434	0.062	0.685	-0.611	0.175	0.00048	0.736	-0.674	0.165	4.3x10^{-5}
rs9375818	A	G	0.338	0.196	0.329	0.55	0.324	-0.814	0.276	0.0032	0.212	-0.401	0.193	0.038	0.380	-0.332	0.144	0.021
rs920811	G	T	0.568	-0.387	0.295	0.19	N/A	N/A	N/A	N/A	0.381	-0.243	0.169	0.15	0.492	-0.430	0.142	0.0024
rs73025532	T	C	0.064	-0.134	0.633	0.83	N/A	N/A	N/A	N/A	0.096	-0.570	0.297	0.055	0.066	-0.578	0.299	0.052
rs515140	T	G	0.457	-0.748	0.274	0.0063	N/A	N/A	N/A	N/A	0.231	-0.436	0.146	0.0029	0.176	-0.534	0.147	0.00029
rs316020	G	A	0.856	-0.717	0.415	0.084	N/A	N/A	N/A	N/A	0.895	-0.853	0.269	0.0015	0.925	-0.643	0.254	0.011
rs62435145	T	G	N/A	N/A	N/A	N/A	0.320	-1.611	0.282	1.1x10^{-8}	0.663	-0.773	0.197	8.8x10^{-5}	0.508	-0.257	0.139	0.065
rs856563	C	T	0.464	-0.888	0.301	0.0032	0.921	-0.574	0.479	0.23	0.630	-0.505	0.160	0.0016	0.510	-0.331	0.131	0.012
rs848486	G	A	0.529	-0.665	0.298	0.026	0.226	-0.442	0.308	0.15	0.412	-0.600	0.160	0.00018	0.359	-0.403	0.139	0.0037
rs10265221	C	T	0.161	-1.512	0.506	0.0028	0.066	-0.680	0.706	0.34	0.291	-0.850	0.183	3.6x10^{-6}	0.175	-0.766	0.185	3.5x10^{-5}
rs6971211	T	C	0.376	-0.473	0.308	0.12	0.440	-0.352	0.259	0.17	0.411	-0.532	0.157	0.00072	0.370	-0.191	0.133	0.15
rs73176866	C	T	0.889	-0.340	0.483	0.48	0.623	-0.559	0.266	0.036	0.876	-0.271	0.237	0.25	0.814	-0.120	0.167	0.47
rs7007761	T	C	0.337	-0.493	0.318	0.12	0.189	-0.906	0.328	0.0057	0.423	-0.623	0.156	6.8x10^{-5}	0.277	-0.276	0.147	0.060
rs4489283	T	C	0.433	-0.800	0.305	0.0086	0.170	-0.878	0.340	0.0099	0.393	-0.174	0.161	0.28	0.495	-0.251	0.135	0.063
rs2001945	C	G	0.778	-0.417	0.368	0.26	0.561	-0.865	0.258	0.00080	0.525	-0.315	0.156	0.043	0.481	-0.047	0.133	0.72
rs61237993	G	A	0.951	-0.241	0.807	0.77	0.453	-0.332	0.262	0.21	0.875	0.123	0.243	0.61	0.775	-0.586	0.171	0.00060
rs10120859	T	C	0.780	-0.396	0.362	0.27	0.784	-0.379	0.312	0.22	0.864	0.056	0.225	0.80	0.889	-0.514	0.214	0.016
rs10746942	G	A	0.409	-0.782	0.303	0.0099	0.297	-0.678	0.279	0.015	0.378	-0.532	0.158	0.00074	0.234	-0.584	0.150	0.00010
rs13283416	G	T	0.411	0.566	0.306	0.064	0.579	-0.491	0.259	0.058	0.421	-0.233	0.157	0.14	0.329	-0.323	0.141	0.022
rs80282103	T	A	0.190	-0.377	0.437	0.39	0.078	-1.108	0.507	0.029	0.091	-1.186	0.297	6.3x10^{-5}	0.101	-0.254	0.224	0.26
rs7475348	C	T	0.736	-0.435	0.369	0.24	0.686	-0.555	0.283	0.050	0.522	-0.449	0.161	0.0054	0.601	-0.251	0.138	0.069
rs4418728	T	G	0.325	-0.482	0.357	0.18	0.627	-0.603	0.265	0.023	0.456	-0.487	0.155	0.0017	0.415	-0.151	0.135	0.26
rs75174967	A	G	N/A	N/A	N/A	N/A	0.153	-0.444	0.356	0.21	N/A	N/A	N/A	N/A	0.037	-0.272	0.349	0.44
rs6892	A	G	N/A	N/A	N/A	N/A	0.735	-0.831	0.288	0.0040	N/A	N/A	N/A	N/A	0.737	-0.176	0.147	0.23
rs4962691	T	C	0.185	-1.087	0.405	0.0073	0.612	-0.189	0.263	0.47	0.568	-0.510	0.158	0.0012	0.541	-0.069	0.135	0.61
rs7482894	T	C	0.297	-0.448	0.349	0.20	0.532	-0.635	0.268	0.018	0.655	-0.310	0.171	0.069	0.684	-0.094	0.150	0.53
rs231889	C	T	0.109	-0.258	0.479	0.59	0.064	-1.096	0.524	0.036	0.161	-0.507	0.213	0.017	0.097	-0.253	0.207	0.22
rs7930738	C	G	0.836	-0.558	0.451	0.22	0.845	-1.044	0.396	0.0083	0.801	-0.277	0.217	0.20	0.819	-0.381	0.200	0.057
rs963837	T	C	0.845	-0.393	0.453	0.39	0.646	-1.009	0.268	0.00017	0.551	-0.582	0.157	0.00022	0.582	-0.364	0.145	0.012
rs11039221	T	C	0.204	-0.527	0.373	0.16	0.258	-0.529	0.293	0.071	0.386	-0.568	0.160	0.00037	0.265	-0.280	0.155	0.071
rs11604451	T	C	0.191	-0.488	0.399	0.22	0.168	-1.033	0.342	0.0025	0.348	-0.061	0.161	0.71	0.207	-0.218	0.172	0.20
rs2063724	T	C	0.680	-0.056	0.326	0.86	0.537	-0.447	0.262	0.088	0.819	-0.232	0.204	0.26	0.681	-0.241	0.142	0.091
rs10774020	C	T	0.506	-0.187	0.305	0.54	0.322	-0.813	0.274	0.0030	0.657	-0.413	0.163	0.011	0.361	-0.477	0.141	0.00070
rs632887	G	A	0.497	-0.750	0.333	0.024	0.293	-0.951	0.286	0.00087	0.415	-0.424	0.161	0.0085	0.398	-0.021	0.137	0.88
rs1275609	G	A	0.405	-0.547	0.333	0.10	0.392	-0.997	0.278	0.00033	0.678	-0.206	0.169	0.22	0.717	-0.022	0.151	0.88

rs79105258	A	C	N/A	N/A	N/A	N/A	0.264	-1.281	0.316	4.9x10^{-5}	N/A	N/A	N/A	N/A	0.003	0.533	3.162	0.87
rs34445998	C	T	0.950	-1.056	0.692	0.13	0.577	-0.660	0.261	0.011	0.891	0.096	0.252	0.70	0.769	-0.317	0.165	0.055
rs584480	C	T	0.376	-0.142	0.321	0.66	0.149	-0.688	0.363	0.058	0.603	-0.318	0.160	0.047	0.422	-0.259	0.133	0.052
rs9920185	C	A	0.826	-0.600	0.397	0.13	0.721	-0.321	0.287	0.26	0.581	-0.376	0.158	0.017	0.632	-0.269	0.137	0.049
rs2486288	C	T	0.820	-1.057	0.405	0.0091	0.940	-0.497	0.543	0.36	0.382	-0.923	0.162	1.1x10^{-8}	0.673	-0.346	0.145	0.017
rs7169329	G	C	0.651	-0.190	0.313	0.54	0.588	-0.539	0.223	0.016	0.618	-0.146	0.150	0.33	0.540	0.037	0.132	0.78
rs35255404	G	A	0.845	-1.085	0.407	0.0077	0.602	-1.302	0.222	4.5x10^{-9}	0.792	-0.904	0.180	5.2x10^{-7}	0.664	-0.067	0.143	0.64
rs10851885	G	A	0.119	-1.314	0.466	0.0048	N/A	N/A	N/A	N/A	0.250	-0.554	0.183	0.0025	0.162	0.010	0.230	0.96
rs76614553	C	T	0.928	-1.072	0.572	0.061	N/A	N/A	N/A	N/A	0.973	-1.073	0.525	0.041	0.978	-1.075	0.541	0.047
rs62035088	G	A	0.924	-2.258	0.705	0.0014	0.873	-0.254	0.398	0.52	0.828	-0.512	0.217	0.018	0.750	-0.035	0.162	0.83
rs11858316	C	T	0.662	-0.247	0.324	0.45	0.503	-0.035	0.262	0.89	0.404	-0.218	0.167	0.19	0.523	-0.034	0.132	0.80
rs13329952	T	C	0.684	-0.616	0.338	0.069	0.945	-0.665	0.523	0.20	0.809	-0.188	0.171	0.27	0.765	-0.379	0.160	0.018
rs4494548	A	G	0.139	-0.507	0.477	0.29	0.043	-0.979	0.606	0.11	0.102	-0.538	0.259	0.038	0.089	0.062	0.292	0.83
rs77924615	G	A	0.917	-0.360	0.648	0.58	0.784	-1.563	0.310	4.6x10^{-7}	0.800	-0.745	0.179	3.1x10^{-5}	0.774	-0.378	0.156	0.015
rs757002	A	G	0.751	0.438	0.435	0.31	0.740	-0.635	0.387	0.10	0.877	-0.426	0.330	0.20	0.838	-0.159	0.186	0.39
rs12935539	C	T	0.226	-0.378	0.355	0.29	0.319	-0.722	0.264	0.0063	0.216	-0.361	0.183	0.049	0.272	-0.468	0.147	0.0014
rs7184096	T	G	0.111	-0.509	0.474	0.28	0.105	-0.706	0.394	0.074	0.174	-0.250	0.201	0.21	0.132	-0.172	0.183	0.35
rs9888796	T	C	0.092	-1.035	0.541	0.056	0.090	-0.464	0.457	0.31	0.266	-0.492	0.177	0.0053	0.209	-0.096	0.164	0.56
rs11641050	C	T	0.687	-0.715	0.320	0.026	0.618	-0.412	0.252	0.10	0.808	-0.394	0.165	0.017	0.635	-0.288	0.133	0.030
rs8052428	T	C	0.338	-0.865	0.317	0.0063	0.391	-0.769	0.309	0.013	0.348	-0.323	0.137	0.019	0.352	-0.190	0.136	0.16
rs2460449	A	G	0.161	-1.276	0.590	0.030	0.023	-0.063	1.078	0.95	0.444	-0.393	0.175	0.025	0.303	-0.317	0.151	0.036
rs11871125	T	C	0.277	-0.925	0.386	0.017	0.174	-0.924	0.403	0.022	0.379	-0.026	0.177	0.88	0.236	-0.156	0.162	0.34
rs4795384	G	C	0.537	-0.612	0.304	0.044	0.809	-0.951	0.328	0.0037	0.747	-0.384	0.185	0.037	0.697	-0.250	0.142	0.080
rs8080123	G	T	0.571	-0.519	0.311	0.095	0.805	-1.324	0.340	9.7x10^{-5}	0.788	-0.595	0.205	0.0037	0.771	-0.529	0.158	0.00079
rs9895661	C	T	0.454	-0.433	0.290	0.14	0.527	-1.206	0.290	3.2x10^{-5}	0.193	-0.519	0.219	0.018	0.498	-0.357	0.122	0.0034
rs887258	C	G	0.230	-0.569	0.346	0.10	0.347	-0.420	0.267	0.12	0.224	-0.552	0.194	0.0044	0.353	-0.243	0.124	0.051
rs1719934	G	A	0.227	-0.915	0.366	0.013	0.222	-0.338	0.312	0.28	0.464	-0.474	0.158	0.0027	0.420	-0.175	0.134	0.19
rs16942751	A	C	0.072	-0.447	0.604	0.46	0.295	-0.505	0.284	0.076	0.097	-0.570	0.271	0.036	0.171	-0.357	0.178	0.044
rs162005	A	G	0.758	-0.191	0.368	0.60	0.716	-0.181	0.282	0.52	0.804	0.110	0.197	0.58	0.756	-0.039	0.164	0.81
rs2337106	C	G	0.130	0.121	0.519	0.82	0.471	-0.591	0.259	0.023	0.489	-0.514	0.155	0.00093	0.510	-0.400	0.139	0.0038
rs896642	C	T	0.376	-0.104	0.328	0.75	0.684	-0.529	0.289	0.067	0.779	-0.456	0.194	0.019	0.737	-0.518	0.156	0.00092
rs8096658	G	C	N/A	N/A	N/A	N/A	0.290	-0.856	0.307	0.0053	0.474	-0.633	0.196	0.0013	0.387	-0.705	0.166	2.1x10^{-5}
rs8108623	A	C	0.595	-0.629	0.337	0.062	0.754	-0.389	0.306	0.20	0.637	-0.208	0.178	0.24	0.671	-0.496	0.171	0.0037
rs7252778	A	C	0.414	-0.749	0.308	0.015	0.333	-0.212	0.273	0.44	0.597	-0.486	0.162	0.0027	0.573	-0.151	0.134	0.26
rs2273684	G	T	0.693	0.402	0.326	0.22	0.217	-0.540	0.310	0.082	0.457	-0.269	0.155	0.083	0.330	-0.234	0.152	0.12
rs17216707	T	C	0.930	-1.641	0.600	0.0063	0.928	-0.810	0.581	0.16	0.803	-0.545	0.205	0.0078	0.682	-0.432	0.147	0.0034
rs1758206	T	C	0.066	0.089	0.667	0.89	0.011	0.329	1.198	0.79	0.084	-0.542	0.286	0.058	0.069	-0.725	0.293	0.013
rs2823139	A	G	0.334	-0.703	0.314	0.025	0.247	-0.104	0.298	0.73	0.339	-0.463	0.165	0.0049	0.357	0.049	0.133	0.72
rs2834317	A	G	0.037	-0.179	0.812	0.83	0.058	0.124	0.570	0.83	0.154	-0.361	0.215	0.092	0.190	-0.606	0.166	0.00025
rs17001943	A	G	0.963	-2.176	0.818	0.0078	0.702	-0.912	0.278	0.0010	0.902	-0.821	0.261	0.0017	0.923	-0.711	0.283	0.012
rs80533	G	A	0.940	-0.307	0.653	0.64	0.974	0.147	0.796	0.85	0.770	-0.214	0.190	0.26	0.538	0.049	0.149	0.74

Chr: chromosome. EAF: effect allele frequency. SE: standard error.

[a]Effect allele is aligned to be eGFR decreasing allele.

[b]Beta/SE are obtained from fixed-effects meta-analysis (inverse variance weighting of allelic effects) and represent absolute decrease in eGFR (ml/min per 1.73m^2) per effect allele.

Supplementary Table 4. Summary of 99% credible sets of variants driving distinct signals of eGFR association in trans-ethnic meta-analysis of up to 312,468 individuals of diverse ancestry.

Locus	Index SNV	Chr	99% credible set			
			SNVs	Length (bp)	Start (bp, b37)	End (bp, b37)
CASP9	rs10927723	1	159	130,937	15,431,823	15,562,759
	rs6664388	1	50	178,025	15,815,579	15,993,603
	rs1763632	1	161	265,774	16,087,164	16,352,937
ZNF436	rs4525087	1	17	41,179	23,667,741	23,708,919
RHOC	rs12722725	1	7	17,033	113,247,898	113,264,930
ANXA9	rs267738	1	5	86,570	150,868,102	150,954,671
GBAP1	rs2070803	1	12	45,849	155,151,754	155,197,602
PMF1-BGLAP	rs2842870	1	12	7,274	156,197,380	156,204,653
CACNA1S	rs3850625	1	2	45,248	200,971,049	201,016,296
SDCCAG8	rs10158537	1	88	122,777	243,378,987	243,501,763
DDX1	rs807603	2	3	10,544	15,782,471	15,793,014
NT5C1B-RDH14	rs13417750	2	33	8,169	18,676,203	18,684,371
GCKR	rs1260326	2	3	11,664	27,730,940	27,742,603
C2orf73	rs10181201	2	17	227,568	54,571,607	54,799,174
	rs7586066	2	1	1	54,810,492	54,810,492
	rs17046036	2	2	28,454	54,827,001	54,855,454
NAT8	rs6546869	2	44	269,122	73,631,779	73,900,900
PSD4-PAX8	rs11123169	2	1	1	113,967,075	113,967,075
ORC4	rs13026220	2	142	401,276	148,555,309	148,956,584
FAP	rs77335736	2	105	83,260	163,054,724	163,137,983
LRP2	rs35472707	2	869	998,835	169,703,974	170,702,808
	rs4140872	2	9	14,402	170,190,725	170,205,126
	rs60641214	2	2,011	999,775	169,703,034	170,702,808
HOXD8	rs187355703	2	20	237,014	176,816,413	177,053,426
NFE2L2	rs35955110	2	29	29,481	178,118,990	178,148,470
CPS1	rs1047891	2	2	2,549	211,540,507	211,543,055
IGFBP5	rs7587010	2	15	18,469	217,665,368	217,683,836
XYLB	rs36070911	3	80	128,291	38,440,237	38,568,527
SLC15A2	rs2250067	3	124	105,386	121,616,610	121,721,995
TFDP2	rs6440003	3	21	61,076	141,087,623	141,148,698
	rs1511299	3	28	142,774	141,682,825	141,825,598
	rs147877018	3	5	72,173	141,796,239	141,868,411
ETV5-KNG1	rs13081203	3	49	67,155	185,304,018	185,371,172
	rs6809651	3	14	54,580	185,769,425	185,824,004
	rs11927941	3	23	4,080	186,432,839	186,436,918
RGS12	rs13108218	4	4	26,087	3,417,845	3,443,931
SHROOM3	rs111449836	4	6	20,971	77,394,018	77,414,988
	rs13106227	4	214	981,230	76,910,813	77,892,042
PRDM8-FGF5	rs12509595	4	3	3,270	81,181,072	81,184,341
NFKB1	rs223401	4	356	522,926	103,424,193	103,947,118
C1QTNF3-AMACR-RAI14	rs10066990	5	29	13,293	34,503,352	34,516,644
DAB2	rs12153248	5	14	54,189	39,367,548	39,421,736
	rs4016485	5	741	991,974	38,933,412	39,925,385
ARL15	rs114118094	5	1,046	998,806	52,792,767	53,791,572
	rs7719168	5	11	22,137	53,292,309	53,314,445
PIK3R1	rs113246091	5	13	10,940	67,739,274	67,750,213

	rs78660602	5	28	21,913	68,036,771	68,058,683
RGS14-SLC34A1	rs3812036	5	2	6,208	176,807,197	176,813,404
HLA-DRB1-HLA-DQA1	rs9368730	6	350	532,576	32,147,157	32,679,732
C6orf1-RPS10-NUDT3	rs6935129	6	84	189,144	34,215,221	34,404,364
VEGFA	rs1214761	6	106	797,855	43,307,861	44,105,715
	rs881858	6	6	1,802	43,804,808	43,806,609
AKAP7-ARG1	rs9375818	6	31	14,353	131,868,219	131,882,571
SLC22A2	rs920811	6	45	157,762	160,479,478	160,637,239
	rs73025532	6	2	6,255	160,537,308	160,543,562
	rs515140	6	18	43,236	160,635,258	160,678,493
	rs316020	6	5	7,724	160,668,041	160,675,764
UNCX	rs62435145	7	2	376	1,286,192	1,286,567
AK125311	rs856563	7	25	45,734	46,708,387	46,754,120
TMEM60	rs848486	7	65	245,057	77,328,100	77,573,156
PRKAG2	rs10265221	7	10	9,532	151,406,005	151,415,536
SHH	rs6971211	7	9	6,592	155,664,686	155,671,277
	rs73176866	7	177	931,506	155,220,093	156,151,598
STC1	rs7007761	8	11	71,793	23,714,992	23,786,784
NRG1	rs4489283	8	57	108,090	32,324,868	32,432,957
TRIB1	rs2001945	8	18	14,100	126,476,873	126,490,972
DCAF12	rs61237993	9	193	466,136	33,664,300	34,130,435
PIP5K1B	rs10120859	9	7	4,398	71,153,062	71,157,459
	rs10746942	9	4	2,534	71,432,174	71,434,707
ASTN2	rs13283416	9	46	341,002	119,144,336	119,485,337
WDR37	rs80282103	10	1	1	899,071	899,071
MYPN	rs7475348	10	35	32,046	69,933,132	69,965,177
CYP26A1	rs4418728	10	51	246,079	94,599,468	94,845,546
SUFU	rs75174967	10	307	989,422	104,079,818	105,069,239
	rs6892	10	883	970,844	104,104,869	105,075,712
FAM53B	rs4962691	10	28	77,690	126,396,511	126,474,200
H19-IGF2-KCNQ1	rs7482894	11	47	62,713	2,116,492	2,179,204
	rs231889	11	21	25,880	2,769,921	2,795,800
MPPED2	rs7930738	11	80	383,020	30,428,275	30,811,294
	rs963837	11	1	1	30,749,090	30,749,090
CELF1-PTPMT1	rs11039221	11	63	451,186	47,391,948	47,843,133
RNASEH2C	rs11604451	11	23	60,248	65,495,211	65,555,458
GAB2	rs2063724	11	256	220,606	77,919,768	78,140,373
SLC6A13	rs10774020	12	9	18,139	343,450	361,588
TSPAN9	rs632887	12	15	38,716	3,353,636	3,392,351
KRR1-PHLDA1	rs1275609	12	1	1	76,271,183	76,271,183
CUX2	rs79105258	12	1	1	111,718,231	111,718,231
DGKH	rs34445998	13	87	82,641	42,704,400	42,787,040
DACH1	rs584480	13	6	3,680	72,345,089	72,348,768
RASGRP1	rs9920185	15	81	45,949	39,261,480	39,307,428
GATM	rs2486288	15	45	99,251	45,625,479	45,724,729
WDR72	rs7169329	15	161	764,152	53,568,048	54,332,199
	rs35255404	15	14	43,601	53,907,948	53,951,548
UBE2Q2	rs10851885	15	21	168,310	76,136,194	76,304,503
	rs76614553	15	208	209,273	76,141,680	76,350,952
CEMIP	rs62035088	15	7	29,125	81,136,718	81,165,842
IGF1R	rs11858316	15	38	63,826	99,249,029	99,312,854

UMOD-PDILT	rs13329952	16	8	8,284	20,359,267	20,367,550
	rs4494548	16	30	35,175	20,371,588	20,406,762
	rs77924615	16	1	1	20,392,332	20,392,332
	rs757002	16	1,087	999,858	19,892,373	20,892,230
SALL1	rs12935539	16	4	5,241	51,754,991	51,760,231
	rs7184096	16	28	33,672	51,755,027	51,788,698
SLC7A6	rs9888796	16	28	132,383	68,294,800	68,427,182
NFAT5	rs11641050	16	86	206,755	69,575,992	69,782,746
	rs8052428	16	371	846,052	69,129,845	69,975,896
DPEP1	rs2460449	16	12	232,725	89,690,688	89,923,412
SLC47A1	rs11871125	17	10	14,836	19,428,610	19,443,445
CDK12-MED1	rs4795384	17	210	343,370	37,396,201	37,739,570
BCAS3	rs8080123	17	19	23,140	59,236,155	59,259,294
	rs9895661	17	1	1	59,456,589	59,456,589
	rs887258	17	5	17,771	59,472,123	59,489,893
EPB41L3	rs1719934	18	39	26,050	5,575,354	5,601,403
PCAT18-AQP4	rs16942751	18	2	6,679	24,386,535	24,393,213
	rs162005	18	49	582,784	24,132,835	24,715,618
SMAD7	rs2337106	18	7	13,290	46,460,903	46,474,192
RNF152	rs896642	18	30	50,289	59,328,786	59,379,074
NFATC1	rs8096658	18	2	435	77,156,103	77,156,537
JUND-LSM4	rs8108623	19	17	25,014	18,383,506	18,408,519
SLC7A9	rs7252778	19	7	7,738	33,356,891	33,364,628
TP53INP2	rs2273684	20	120	317,567	33,356,511	33,674,077
BCAS1	rs17216707	20	1	1	52,732,362	52,732,362
ARFRP1	rs1758206	20	58	119,827	62,275,492	62,395,318
NRIP1	rs2823139	21	5	5,928	16,576,783	16,582,710
ATP50	rs2834317	21	7	97,802	35,260,961	35,358,762
MKL1	rs17001943	22	55	91,111	40,816,841	40,907,951
	rs80533	22	143	849,107	40,528,220	41,377,326

Chr: chromosome.

Supplementary Table 5. High-confidence variants driving distinct eGFR association signals, identified through fine-mapping under an annotation informed prior model of causality, applied to association summary statistics from the trans-ethnic meta-analysis of up to 312,468 individuals of diverse ancestry.

Locus	Index SNV	High-confidence variant	Chr	Position (bp, b37)	p-value	Posterior probability	Enriched annotations			
							Coding exon	HDAC2 binding site	EZH2 binding site	Kidney-specific histone modification
ZNF436	rs4525087	rs2849028	1	23,688,933	1.0×10^{-12}	59.5				Yes
ANXA9	rs267738	rs267738	1	150,940,625	1.7×10^{-10}	55.3	Yes			
CACNA1S	rs3850625	rs3850625	1	201,016,296	2.5×10^{-9}	99.0	Yes			
GCKR	rs1260326	rs1260326	2	27,730,940	2.0×10^{-35}	86.1	Yes			
C2orf73	rs10181201	rs10181201	2	54,799,174	7.4×10^{-8}	60.9				Yes
C2orf73	rs7586066	rs7586066	2	54,810,492	9.7×10^{-13}	99.9				Yes
C2orf73	rs17046036	rs17046036	2	54,855,454	1.9×10^{-15}	98.4				Yes
PSD4-PAX8	rs11123169	rs11123169	2	113,967,075	8.1×10^{-15}	99.4				Yes
LRP2	rs35472707	rs35472707	2	169,995,581	1.1×10^{-6}	64.3				
LRP2	rs60641214	rs60641214	2	170,199,292	5.6×10^{-8}	64.9				Yes
HOXD8	rs187355703	rs187355703	2	176,993,583	1.4×10^{-9}	64.2			Yes	Yes
CPS1	rs1047891	rs1047891	2	211,540,507	1.5×10^{-29}	98.1	Yes			
RGS12	rs13108218	rs13108218	4	3,443,931	1.4×10^{-8}	92.8				Yes
PRDM8-FGF5	rs12509595	rs12509595	4	81,182,554	4.7×10^{-16}	57.1				
RGS14-SLC34A1	rs3812036	rs3812036	5	176,813,404	1.0×10^{-32}	65.0				Yes
SLC22A2	rs73025532	rs461473	6	160,543,562	6.4×10^{-9}	58.0				Yes
UNCX	rs62435145	rs62435145	7	1,286,567	2.0×10^{-39}	56.0			Yes	
AK125311	rs856563	rs856563	7	46,723,510	5.1×10^{-10}	52.9				
SHH	rs6971211	rs6971211	7	155,664,686	1.4×10^{-12}	60.4				
STC1	rs7007761	rs7007761	8	23,719,571	2.8×10^{-23}	73.7		Yes		
PIP5K1B	rs10120859	rs10120859	9	71,156,295	4.8×10^{-10}	69.6				Yes
PIP5K1B	rs10746942	rs2039424	9	71,432,174	1.3×10^{-26}	50.7		Yes		
WDR37	rs80282103	rs80282103	10	899,071	2.0×10^{-18}	100.0				
CYP26A1	rs4418728	rs4418728	10	94,839,724	1.4×10^{-8}	58.2				
MPPED2	rs7930738	rs7930738	11	30,605,859	4.7×10^{-7}	51.5				Yes
MPPED2	rs963837	rs963837	11	30,749,090	9.3×10^{-37}	99.8				
TSPAN9	rs632887	rs632887	12	3,392,351	3.7×10^{-9}	83.7				
KRR1-PHLDA1	rs1275609	rs1275609	12	76,271,183	1.2×10^{-8}	99.7				Yes
CUX2	rs79105258	rs79105258	12	111,718,231	5.7×10^{-15}	100.0				
CEMIP	rs62035088	rs62035088	15	81,165,842	8.2×10^{-9}	62.9				

UMOD-PDILT	rs77924615	rs77924615	16	20,392,332	1.5×10^{-54}	100.0				
SALL1	rs12935539	rs12935539	16	51,754,991	1.9×10^{-18}	76.1				
DPEP1	rs2460449	rs2460449	16	89,700,747	4.2×10^{-9}	97.8				Yes
BCAS3	rs9895661	rs9895661	17	59,456,589	8.9×10^{-28}	100.0		Yes		Yes
BCAS3	rs887258	rs887258	17	59,479,580	2.7×10^{-13}	62.2			Yes	Yes
PCAT18-AQP4	rs16942751	rs16942751	18	24,393,213	4.7×10^{-12}	92.8				
SMAD7	rs2337106	rs2337106	18	46,460,903	6.0×10^{-9}	53.4				Yes
NFATC1	rs8096658	rs8096658	18	77,156,537	1.7×10^{-28}	58.5		Yes	Yes	
BCAS1	rs17216707	rs17216707	20	52,732,362	4.6×10^{-16}	99.9				Yes
NRIP1	rs2823139	rs2823139	21	16,576,783	3.7×10^{-9}	50.5				Yes

Chr: chromosome.

Supplementary Table 6. Association summary statistics for high-confidence variants for alternative measures of kidney function: (i) eGFR calculated from cystatin C, obtained from up to 24,061 individuals of European ancestry from the CKDGen Consortium; (ii) blood urea nitrogen, obtained from up to 139,818 individuals of East Asian ancestry from the Biobank Japan Project; and (iii) urine albumin to creatinine ratio, obtained from up to 46,061 non-diabetic individuals of European ancestry from the CKDGen Consortium.

Locus	Index SNV	High-confidence variant	Alleles[a]		eGFR cystatin C			Blood urea nitrogen			Urine albumin creatinine ratio		
			Effect	Other	Beta	SE	*p*-value	Beta	SE	*p*-value	Beta	SE	*p*-value
ZNF436	rs4525087	rs2849028	G	A	-0.0017	0.0025	0.50	0.0045	0.0036	0.20	-0.0056	0.0071	0.43
ANXA9	rs267738	rs267738	T	G	-0.0035	0.0027	0.19	-0.0004	0.0085	0.96	-0.0034	0.0072	0.64
CACNA1S	rs3850625	rs3850625	G	A	-0.0018	0.0033	0.60	-0.0060	0.0097	0.54	-0.0240	0.0110	0.032
GCKR	rs1260326	rs1260326	C	T	-0.0058	0.0023	0.0085	-0.0117	0.0036	0.0010	-0.0250	0.0061	$5.2x10^{-5}$
C2orf73	rs10181201	rs10181201	G	A	-0.0016	0.0048	0.74	-0.0131	0.0075	0.081			
C2orf73	rs7586066	rs7586066	A	G	0.0054	0.0028	0.053	0.0073	0.0051	0.16	-0.0006	0.0087	0.94
C2orf73	rs17046036	rs17046036	A	G	-0.0052	0.0029	0.070	0.0001	0.0065	0.99	-0.0140	0.0094	0.13
PSD4-PAX8	rs11123169	rs11123169	C	T	-0.0038	0.0023	0.097	0.0456	0.0040	$3.2x10^{-30}$	0.0042	0.0067	0.53
LRP2	rs35472707	rs35472707	T	C	-0.0071	0.0050	0.16						
LRP2	rs60641214	rs60641214	A	T	-0.0042	0.0028	0.12	-0.0028	0.0039	0.48			
HOXD8	rs187355703	rs187355703	G	C	-0.0103	0.0065	0.11						
CPS1	rs1047891	rs1047891	A	C	0.0022	0.0024	0.37	-0.0256	0.0053	$1.3x10^{-6}$			
RGS12	rs13108218	rs13108218	A	G	0.0028	0.0026	0.27	0.0002	0.0036	0.95			
PRDM8-FGF5	rs12509595	rs12509595	T	C	0.0023	0.0025	0.35	0.0223	0.0039	$1.6x10^{-8}$			
RGS14-SLC34A1	rs3812036	rs3812036	T	C	-0.0069	0.0026	0.0083	0.0163	0.0041	$7.9x10^{-5}$	0.0140	0.0072	0.054
SLC22A2	rs73025532	rs461473	A	G	-0.0044	0.0037	0.23				-0.0330	0.0130	0.0081
UNCX	rs62435145	rs62435145	T	G	-0.0063	0.0037	0.085	0.0450	0.0039	$9.1x10^{-31}$			
AK125311	rs856563	rs856563	C	T	-0.0024	0.0023	0.28	0.0073	0.0065	0.26	-0.0062	0.0064	0.33
SHH	rs6971211	rs6971211	T	C	-0.0052	0.0024	0.023	0.0060	0.0036	0.092	-0.0070	0.0077	0.37
STC1	rs7007761	rs7007761	T	C	-0.0062	0.0022	0.0048	0.0243	0.0046	$1.5x10^{-7}$	-0.0078	0.0064	0.22
PIP5K1B	rs10120859	rs10120859	T	C	-0.0019	0.0032	0.54	0.0075	0.0042	0.077	0.0094	0.0091	0.30
PIP5K1B	rs10746942	rs2039424	G	A	-0.0063	0.0023	0.0049	0.0104	0.0038	0.0061			
WDR37	rs80282103	rs80282103	T	A	-0.0093	0.0041	0.025	0.0300	0.0070	$1.7x10^{-5}$			
CYP26A1	rs4418728	rs4418728	T	G	-0.0047	0.0024	0.043	0.0059	0.0037	0.11	-0.0120	0.0081	0.13
MPPED2	rs7930738	rs7930738	C	G	-0.0007	0.0029	0.80	0.0214	0.0052	$4.4x10^{-5}$			
MPPED2	rs963837	rs963837	T	C	-0.0075	0.0023	0.00082	0.0435	0.0041	$2.2x10^{-26}$	0.0001	0.0064	0.99
TSPAN9	rs632887	rs632887	G	A	-0.0009	0.0023	0.69	0.0049	0.0039	0.21	-0.0026	0.0062	0.68
KRR1-PHLDA1	rs1275609	rs1275609	G	A	-0.0028	0.0025	0.25	0.0226	0.0039	$5.6x10^{-9}$			
CUX2	rs79105258	rs79105258	A	C				0.0463	0.0044	$1.3x10^{-25}$			
CEMIP	rs62035088	rs62035088	G	A	0.0002	0.0033	0.95	0.0054	0.0054	0.32			

UMOD-PDILT	rs77924615	rs77924615	G	A	-0.0153	0.0031	$5.3x10^{-7}$	0.0465	0.0043	$2.2x10^{-27}$			
SALL1	rs12935539	rs12935539	C	T	-0.0039	0.0027	0.15	0.0080	0.0039	0.037			
DPEP1	rs2460449	rs2460449	A	G	-0.0046	0.0024	0.055						
BCAS3	rs9895661	rs9895661	C	T	-0.0022	0.0030	0.45	-0.0052	0.0041	0.20	0.0120	0.0097	0.23
BCAS3	rs887258	rs887258	C	G	-0.0002	0.0028	0.93	-0.0024	0.0037	0.51	0.0098	0.0074	0.18
PCAT18-AQP4	rs16942751	rs16942751	A	C	-0.0036	0.0037	0.33	0.0175	0.0040	$9.3x10^{-6}$	-0.0090	0.0120	0.47
SMAD7	rs2337106	rs2337106	C	G	0.0001	0.0025	0.96	0.0055	0.0036	0.12	-0.0044	0.0074	0.55
NFATC1	rs8096658	rs8096658	G	C	-0.0014	0.0036	0.70	0.0257	0.0044	$6.3x10^{-9}$			
BCAS1	rs17216707	rs17216707	T	C	-0.0089	0.0028	0.0014	0.0227	0.0074	0.0021	0.0110	0.0087	0.19
NRIP1	rs2823139	rs2823139	A	G	-0.0021	0.0023	0.36	0.0111	0.0041	0.0066	0.0004	0.0062	0.95

SE: standard error.

[a]Effect allele is aligned to be eGFR decreasing allele.

Supplementary Table 7. Co-localisation of high-confidence SNVs for eGFR and lead eQTL variants in the TRANSLATE Study and TCGA.

Locus	Chr	High-confidence SNV for eGFR	Position (bp, b37)	Gene	Lead eQTL variant	Position (bp, b37)	European r^2	Alleles[a]		TRANSLATE and TCGA meta-analysis		
								Effect	Other	beta	SE	*p*-value
PRDM8-FGF5	4	rs12509595	81,182,554	*FGF5*	rs13125101	81,174,592	0.96	G	A	-0.759	0.066	5.9×10^{-31}
UMOD-PDILT	16	rs77924615	20,392,332	*UMOD*	rs77924615	20,392,332	Same SNV	G	A	0.303	0.061	7.1×10^{-7}
				GP2	rs77924615	20,392,332	Same SNV	G	A	0.227	0.050	6.2×10^{-6}
BCAS3	17	rs887258	59,479,580	*TBX2*	rs1476781	59,476,415	0.98	T	C	0.171	0.042	4.6×10^{-5}

Chr: chromosome. SE: standard error.
[a]Effect allele is aligned to be eGFR decreasing allele.

Supplementary Table 8. Two sample Mendelian randomisation analyses to assess causal effect of eGFR on clinically-relevant kidney and cardiovascular outcomes.

(a) Exposure association summary statistics from the COGENT-Kidney Consortium (58,293 individuals of diverse ancestry).

Outcome	Sample size (cases/controls)	SNVs	Inverse variance weighted regression				Weighted median regression			MR-EGGER regression			
			beta (x10^3)	SE (x10^3)	*p*-value	*Q p*-value	beta (x10^3)	SE (x10^3)	*p*-value	beta (x10^3)	SE (x10^3)	*p*-value	*Q p*-value
Chronic kidney disease[a]	12,385/104,780	28	-85.013	9.506	**3.8x10^{-19}**	0.15	-71.405	13.470	**1.2x10^{-7}**	-52.573	22.063	0.025	0.22
Chronic kidney disease stage 5[a]	4,905/447,359	65	-0.562	0.073	**1.1x10^{-14}**	0.92	-0.438	0.111	**7.5x10^{-5}**	-0.254	0.189	0.18	0.95
Glomerular diseases[a]	2,289/449,975	72	-0.180	0.050	**0.00029**	0.89	-0.184	0.076	0.016	-0.086	0.120	0.48	0.89
Hypertensive renal disease	1,663/450,601	72	-0.103	0.042	0.015	0.72	-0.041	0.064	0.52	0.072	0.107	0.50	0.78
IgA nephropathy	3,211/8,735	72	22.304	13.122	0.089	0.99	26.153	18.460	0.16	-3.441	31.327	0.91	0.99
Calculus of kidney and ureter[b]	5,216/447,048	54	0.543	0.094	**8.1x10^{-9}**	0.92	0.444	0.136	**0.0011**	0.364	0.251	0.15	0.92
Diastolic blood pressure	317,756	47	-3.171	1.072	**0.0031**	0.98	-3.287	1.551	0.034	-3.451	2.957	0.25	0.97
Systolic blood pressure	317,654	50	-0.737	1.137	0.52	0.092	-0.187	1.640	0.91	2.068	3.158	0.52	0.092
Essential (primary) hypertension[c]	84,640/367,624	50	-0.728	0.316	0.021	0.34	-0.641	0.437	0.14	-1.008	0.836	0.23	0.30
Coronary heart disease	60,801/123,504	66	3.033	3.158	0.34	0.67	-0.947	4.737	0.84	-1.718	7.710	0.82	0.65
Myocardial infarction	43,676/128,199	68	5.957	3.416	0.081	0.82	-1.639	5.050	0.75	-6.951	8.435	0.41	0.87
Ischemic stroke	10,307/19,326	36	14.862	7.689	0.053	0.44	12.372	11.076	0.26	6.275	18.243	0.73	0.40

SE: standard error. [a]Individual SNV causal effects presented in Figure 2. [b]Individual SNV causal effects presented in Figure 3. [c]Individual SNV causal effects presented in Figure 4.

(b) Exposure association summary statistics from the CKDGen Consortium (110,517 individuals of European ancestry).

Outcome	Sample size (cases/controls)	SNVs	Inverse variance weighted regression				Weighted median regression			MR-EGGER regression			
			beta (x10)	SE (x10)	*p*-value	*Q p*-value	beta (x10)	SE (x10)	*p*-value	beta (x10)	SE (x10)	*p*-value	*Q p*-value
Chronic kidney disease[a]	12,385/104,780	27	-57.199	5.414	**4.3x10^{-26}**	0.78	-53.738	8.380	**1.4x10^{-10}**	-59.971	14.144	**0.00027**	0.74
Chronic kidney disease stage 5[a]	4,905/447,359	63	-0.386	0.487	**2.1x10^{-15}**	0.97	-0.400	0.071	**1.7x10^{-8}**	-0.269	0.119	0.027	0.97
Glomerular diseases[a]	2,289/449,975	69	-0.121	0.032	**0.00017**	0.92	-0.109	0.047	0.020	0.099	0.075	0.19	0.93
Hypertensive renal disease	1,663/450,601	69	-0.071	0.028	0.010	0.69	-0.018	0.042	0.67	0.037	0.068	0.59	0.75
IgA nephropathy	3,211/8,735	69	16.440	8.666	0.058	0.99	18.707	12.841	0.15	12.630	20.562	0.54	0.99
Calculus of kidney and ureter[b]	5,216/447,048	51	0.324	0.062	**1.5x10^{-7}**	0.90	0.025	0.010	0.012	0.200	0.153	0.20	0.90
Diastolic blood pressure[c]	317,756	44	-2.009	0.687	**0.0035**	0.95	-2.169	1.012	0.032	-1.592	1.676	0.35	0.94
Systolic blood pressure	317,654	47	-0.675	0.774	0.38	0.076	-0.295	1.076	0.78	0.488	1.926	0.80	0.069
Essential (primary) hypertension[c]	84,640/367,624	47	-0.508	0.213	0.017	0.31	-0.342	0.319	0.28	-0.355	0.522	0.50	0.28
Coronary heart disease	60,801/123,504	64	3.654	2.071	0.078	0.74	3.111	3.218	0.33	7.617	4.748	0.11	0.73
Myocardial infarction	43,676/128,199	65	5.035	2.237	0.024	0.85	3.750	3.429	0.27	3.649	5.178	0.48	0.84
Ischemic stroke	10,307/19,326	35	8.050	5.182	0.12	0.35	7.527	7.248	0.30	-5.577	13.527	0.68	0.36

SE: standard error. [a]Individual SNV causal effects presented in Figure 2. [b]Individual SNV causal effects presented in Figure 3. [c]Individual SNV causal effects presented in Figure 4.

(c) Exposure association summary statistics from the Biobank Japan Project (143,658 individuals of East Asian ancestry).

Outcome	Sample size (cases/controls)	SNVs	Inverse variance weighted regression				Weighted median regression			MR-EGGER regression			
			**beta (x10)**	**SE (x10)**	***p*-value**	***Q p*-value**	**beta (x10)**	**SE (x10)**	***p*-value**	**beta (x10)**	**SE (x10)**	***p*-value**	***Q p*-value**
Chronic kidney disease[a]	12,385/104,780	27	-12.389	1.272	**2.1x10^{-22}**	0.55	-10.800	2.023	**9.3x10^{-8}**	-10.300	3.667	0.0095	0.51
Chronic kidney disease stage 5[a]	4,905/447,359	59	-0.102	0.126	**6.0x10^{-16}**	0.96	-0.894	0.182	**8.6x10^{-7}**	-0.669	0.364	0.072	0.96
Glomerular diseases[a]	2,289/449,975	66	-0.030	0.079	**0.00016**	0.93	-0.026	0.012	0.030	-0.022	0.020	0.29	0.92
Hypertensive renal disease	1,663/450,601	64	-0.021	0.007	**0.0036**	0.75	-0.013	0.011	0.55	-0.013	0.021	0.55	0.73
IgA nephropathy	3,211/8,735	65	4.365	2.032	0.032	0.99	5.362	3.016	0.075	5.546	5.909	0.35	0.99
Calculus of kidney and ureter[b]	5,216/447,048	50	0.074	0.014	**8.2x10^{-8}**	0.93	0.058	0.020	0.0043	0.040	0.042	0.35	0.93
Diastolic blood pressure[c]	317,756	43	-0.484	0.174	0.0054	0.96	-0.495	0.238	0.037	-0.512	0.557	0.36	0.95
Systolic blood pressure	317,654	47	-0.281	0.180	0.12	0.13	-0.576	0.256	0.024	-0.600	0.555	0.29	0.11
Essential (primary) hypertension[c]	84,640/367,624	47	-0.129	0.051	0.012	0.45	-0.094	0.075	0.21	-0.189	0.173	0.28	0.42
Coronary heart disease	60,801/123,504	61	0.419	0.517	0.42	0.67	-0.117	0.803	0.88	-1.588	1.347	0.24	0.72
Myocardial infarction	43,676/128,199	62	0.744	0.560	0.18	0.92	0.258	0.841	0.76	-2.185	1.457	0.14	0.97
Ischemic stroke	10,307/19,326	35	1.704	1.232	0.17	0.35	-0.987	1.763	0.58	-2.893	3.371	0.40	0.41

SE: standard error. [a]Individual SNV causal effects presented in Figure 2. [b]Individual SNV causal effects presented in Figure 3. [c]Individual SNV causal effects presented in Figure 4.

Supplementary Table 9. Association summary statistics for lead eGFR SNVs (non-palindromic) for clinically relevant kidney outcomes on which kidney function has a causal effect in Mendelian randomisation analyses.

Locus	Lead SNV	Alleles		CKD		CKD stage 5		Glomerular diseases		Calculus of kidney/ureter	
		Effect[a]	Other	Beta (SE)	*p*-value	Beta (SE) x10^{3}	*p*-value	Beta (SE) x10^{3}	*p*-value	Beta (SE) x10^{3}	*p*-value
CASP9	rs45619934	T	G	NA	NA	0.073 (0.233)	0.75	0.018 (0.160)	0.91	-0.163 (0.238)	0.49
ZNF436	rs4525087	C	A	NA	NA	0.106 (0.252)	0.68	-0.154 (0.173)	0.37	-0.918 (0.258)	0.00037
RHOC	rs12722725	C	T	NA	NA	-0.063 (0.323)	0.85	-0.380 (0.222)	0.088	-0.149 (0.331)	0.65
ANXA9	rs267738	T	G	0.052 (0.019)	0.0055	0.888 (0.261)	0.00066	-0.306 (0.180)	0.088	-1.176 (0.267)	1.1x10^{-5}
GBAP1	rs2070803	A	G	NA	NA	0.008 (0.218)	0.97	-0.055 (0.150)	0.71	0.029 (0.224)	0.90
PMF1-BGLAP	rs2842870	T	C	NA	NA	0.095 (0.225)	0.67	-0.309 (0.155)	0.047	-0.040 (0.231)	0.86
CACNA1S	rs3850625	G	A	0.037 (0.024)	0.13	-0.021 (0.334)	0.95	-0.194 (0.230)	0.40	-0.035 (0.342)	0.92
DDX1	rs807603	T	C	NA	NA	0.207 (0.224)	0.36	-0.002 (0.155)	0.99	-0.841 (0.230)	0.00025
NT5C1B-RDH14	rs13417750	A	G	0.045 (0.016)	0.0043	0.910 (0.227)	6.3x10^{-5}	0.379 (0.157)	0.016	-0.282 (0.233)	0.23
GCKR	rs1260326	C	T	0.023 (0.015)	0.14	0.214 (0.221)	0.33	0.108 (0.152)	0.48	-0.791 (0.226)	0.00047
C2orf73	rs1527649	C	T	0.013 (0.018)	0.48	0.135 (0.270)	0.62	-0.049 (0.186)	0.79	-0.018 (0.277)	0.95
NAT8	rs6546869	G	A	NA	NA	-0.023 (0.261)	0.93	-0.243 (0.179)	0.18	0.175 (0.267)	0.51
PSD4-PAX8	rs11123169	C	T	0.037 (0.017)	0.028	-0.142 (0.234)	0.54	-0.037 (0.161)	0.82	-0.021 (0.240)	0.93
ORC4	rs13026220	G	A	NA	NA	0.586 (0.237)	0.013	0.300 (0.163)	0.065	-0.168 (0.242)	0.49
FAP	rs77335736	C	T	NA	NA	1.677 (1.088)	0.12	1.189 (0.749)	0.11	-1.147 (1.114)	0.30
LRP2	rs3770636	T	G	NA	NA	-0.092 (0.933)	0.92	-0.755 (0.643)	0.24	-1.363 (0.956)	0.15
NFE2L2	rs35955110	C	T	NA	NA	0.542 (0.234)	0.020	0.176 (0.161)	0.27	0.299 (0.239)	0.21
CPS1	rs1047891	A	C	NA	NA	0.375 (0.232)	0.11	-0.092 (0.160)	0.57	0.098 (0.238)	0.68
IGFBP5	rs7587010	T	G	NA	NA	-0.019 (0.220)	0.93	0.274 (0.151)	0.070	-0.246 (0.225)	0.27
XYLB	rs36070911	G	A	NA	NA	0.320 (0.221)	0.15	-0.115 (0.152)	0.45	-0.286 (0.227)	0.21
SLC15A2	rs2250067	T	C	NA	NA	0.633 (0.217)	0.0035	0.111 (0.149)	0.46	-0.421 (0.222)	0.057
TFDP2	rs1511299	T	C	NA	NA	0.225 (0.247)	0.36	0.127 (0.170)	0.45	0.411 (0.252)	0.10
ETV5-KNG1	rs13081203	A	G	NA	NA	0.231 (0.228)	0.31	0.217 (0.157)	0.17	0.160 (0.233)	0.49
RGS12	rs13108218	G	A	NA	NA	0.028 (0.224)	0.90	-0.344 (0.154)	0.026	0.291 (0.229)	0.20
SHROOM3	rs142647267	T	C	NA	NA	NA	NA	NA	NA	NA	NA
PRDM8-FGF5	rs12509595	T	C	NA	NA	0.817 (0.238)	0.00059	0.113 (0.164)	0.49	-0.061 (0.243)	0.80
NFKB1	rs223401	T	C	0.036 (0.017)	0.035	0.550 (0.230)	0.017	-0.020 (0.158)	0.90	0.067 (0.235)	0.77
C1QTNF3-AMACR-RAI14	rs10066990	A	G	0.008 (0.016)	0.60	0.193 (0.224)	0.39	0.059 (0.154)	0.70	-0.656 (0.229)	0.0042
DAB2	rs13179493	C	T	0.079 (0.019)	3.1x10^{-5}	-0.096 (0.241)	0.69	-0.151 (0.166)	0.36	-0.483 (0.246)	0.050
ARL15	rs7719168	A	C	NA	NA	0.860 (0.343)	0.012	0.055 (0.236)	0.82	0.276 (0.351)	0.43
PIK3R1	rs113246091	A	G	NA	NA	-0.071 (0.368)	0.85	0.050 (0.253)	0.84	0.752 (0.377)	0.046
RGS14-SLC34A1	rs3812036	T	C	0.096 (0.018)	7.7x10^{-8}	0.639 (0.254)	0.012	0.591 (0.175)	0.00072	1.458 (0.260)	2.0x10^{-8}

HLA-DRB1-HLA-DQA1	rs117463603	G	A	NA	NA	NA	NA	NA	NA	NA	NA
C6orf1-RPS10-NUDT3	rs6935129	A	G	NA	NA	-0.025 (0.572)	0.97	0.296 (0.394)	0.45	-0.619 (0.585)	0.29
VEGFA	rs881858	A	G	0.070 (0.018)	$8.7x10^{-5}$	0.444 (0.234)	0.057	0.467 (0.161)	0.0038	-0.719 (0.239)	0.0027
AKAP7-ARG1	rs9375818	A	G	NA	NA	0.188 (0.262)	0.47	0.350 (0.180)	0.053	-0.383 (0.268)	0.15
SLC22A2	rs316020	G	A	0.042 (0.025)	0.091	0.140 (0.356)	0.70	0.324 (0.245)	0.19	0.551 (0.364)	0.13
UNCX	rs62435145	T	G	NA	NA	0.708 (0.241)	0.0034	0.130 (0.166)	0.44	-0.291 (0.247)	0.24
AK125311	rs856563	C	T	-0.010 (0.016)	0.54	0.324 (0.223)	0.15	0.125 (0.154)	0.42	-0.699 (0.229)	0.0022
TMEM60	rs848486	G	A	NA	NA	0.044 (0.219)	0.84	0.120 (0.151)	0.43	0.184 (0.224)	0.41
PRKAG2	rs10265221	C	T	NA	NA	0.123 (0.239)	0.61	0.204 (0.164)	0.22	-1.031 (0.245)	$2.5x10^{-5}$
SHH	rs6971211	T	C	0.018 (0.015)	0.24	0.261 (0.235)	0.27	0.026 (0.162)	0.87	-0.870 (0.241)	0.00030
STC1	rs7007761	T	C	0.058 (0.016)	0.00020	0.471 (0.220)	0.032	0.431 (0.151)	0.0044	-0.046 (0.225)	0.84
NRG1	rs4489283	T	C	NA	NA	0.075 (0.223)	0.74	-0.051 (0.154)	0.74	-0.347 (0.228)	0.13
DCAF12	rs61237993	G	A	NA	NA	0.290 (0.325)	0.37	-0.374 (0.223)	0.094	-0.932 (0.332)	0.0050
PIP5K1B	rs2039424	G	A	NA	NA	0.350 (0.223)	0.12	0.107 (0.154)	0.49	0.145 (0.228)	0.52
ASTN2	rs13283416	G	T	0.030 (0.015)	0.047	0.163 (0.219)	0.46	-0.069 (0.151)	0.65	-0.320 (0.224)	0.15
MYPN	rs7475348	C	T	NA	NA	0.734 (0.217)	0.00071	0.259 (0.149)	0.083	-0.252 (0.222)	0.26
CYP26A1	rs4418728	T	G	0.024 (0.015)	0.13	0.379 (0.217)	0.081	0.224 (0.150)	0.13	-0.063 (0.222)	0.78
SUFU	rs6892	A	G	-0.014 (0.020)	0.48	-0.044 (0.285)	0.88	0.052 (0.196)	0.79	-0.087 (0.292)	0.77
FAM53B	rs4962691	T	C	0.011 (0.015)	0.45	0.200 (0.220)	0.36	-0.185 (0.151)	0.22	-0.313 (0.225)	0.16
H19-IGF2-KCNQ1	rs7482894	T	C	0.015 (0.018)	0.39	0.049 (0.228)	0.83	0.180 (0.157)	0.25	-0.819 (0.233)	0.00044
MPPED2	rs963837	T	C	0.082 (0.015)	$9.0x10^{-8}$	0.592 (0.217)	0.0064	0.224 (0.149)	0.13	-0.279 (0.222)	0.21
CELF1-PTPMT1	rs11039221	T	C	NA	NA	0.355 (0.222)	0.11	0.055 (0.153)	0.72	-0.128 (0.227)	0.57
RNASEH2C	rs11604451	T	C	NA	NA	1.063 (0.227)	$3.0x10^{-6}$	0.029 (0.157)	0.85	-0.733 (0.233)	0.0016
GAB2	rs2063724	T	C	0.007 (0.020)	0.73	0.970 (0.288)	0.00077	0.278 (0.198)	0.16	-0.190 (0.295)	0.52
SLC6A13	rs10774020	C	T	NA	NA	0.356 (0.230)	0.12	-0.119 (0.158)	0.45	-0.185 (0.235)	0.43
TSPAN9	rs632887	G	A	0.035 (0.016)	0.027	-0.300 (0.222)	0.18	0.285 (0.153)	0.062	-0.505 (0.227)	0.026
KRR1-PHLDA1	rs1275609	G	A	NA	NA	0.104 (0.234)	0.66	0.074 (0.161)	0.65	-0.315 (0.240)	0.19
CUX2	rs79105258	A	C	NA	NA	-1.717 (18.452)	0.93	-6.713 (12.705)	0.60	27.264 (18.885)	0.15
DGKH	rs34445998	C	T	NA	NA	1.061 (0.354)	0.0027	0.224 (0.243)	0.36	-1.258 (0.362)	0.00051
DACH1	rs584480	C	T	0.035 (0.015)	0.021	-0.389 (0.221)	0.079	0.071 (0.152)	0.64	-0.028 (0.226)	0.90
RASGRP1	rs9920185	C	A	NA	NA	0.243 (0.219)	0.27	0.175 (0.150)	0.24	-0.372 (0.223)	0.096
GATM	rs2486288	C	T	NA	NA	0.570 (0.224)	0.011	0.109 (0.154)	0.48	-0.300 (0.229)	0.19
UBE2Q2	rs11636251	T	C	0.046 (0.015)	0.0024	0.033 (0.217)	0.88	0.016 (0.149)	0.91	-0.163 (0.222)	0.46
CEMIP	rs62035088	G	A	NA	NA	0.150 (0.289)	0.61	-0.135 (0.199)	0.50	-0.026 (0.296)	0.93
IGF1R	rs11858316	C	T	-0.004 (0.016)	0.79	-0.122 (0.222)	0.58	0.011 (0.153)	0.94	-0.442 (0.227)	0.051
UMOD-PDILT	rs77924615	G	A	NA	NA	1.573 (0.274)	$9.6x10^{-9}$	0.267 (0.189)	0.16	-2.315 (0.281)	$1.6x10^{-16}$
SALL1	rs12935539	C	T	NA	NA	0.187 (0.260)	0.47	-0.181 (0.179)	0.31	-0.195 (0.266)	0.46

SLC7A6	rs9888796	T	C	NA	NA	0.529 (0.248)	0.033	-0.063 (0.171)	0.71	0.115 (0.254)	0.65
NFAT5	rs11641050	C	T	NA	NA	-0.032 (0.277)	0.91	-0.019 (0.191)	0.92	-0.130 (0.283)	0.65
DPEP1	rs2460449	A	G	NA	NA	0.070 (0.219)	0.75	-0.101 (0.151)	0.50	-0.812 (0.224)	0.00030
SLC47A1	rs11871125	T	C	NA	NA	0.295 (0.223)	0.19	0.027 (0.153)	0.86	-0.096 (0.228)	0.67
BCAS3	rs9895661	C	T	0.067 (0.021)	0.0011	0.221 (0.288)	0.44	0.095 (0.198)	0.63	-1.019 (0.295)	0.00055
EPB41L3	rs1719934	G	A	0.016 (0.016)	0.32	0.209 (0.217)	0.34	-0.035 (0.150)	0.82	-0.525 (0.222)	0.018
PCAT18-AQP4	rs16942751	A	C	0.070 (0.028)	0.011	0.397 (0.385)	0.30	0.017 (0.265)	0.95	-0.252 (0.394)	0.52
RNF152	rs896642	C	T	0.079 (0.020)	0.00011	0.685 (0.263)	0.0092	0.210 (0.181)	0.25	0.663 (0.269)	0.014
JUND-LSM4	rs8108623	A	C	NA	NA	-0.254 (0.237)	0.28	0.093 (0.163)	0.57	0.111 (0.242)	0.65
SLC7A9	rs7252778	A	C	NA	NA	-0.084 (0.221)	0.70	0.065 (0.152)	0.67	-0.338 (0.226)	0.13
TP53INP2	rs2273684	G	T	0.046 (0.015)	0.0021	0.370 (0.219)	0.092	0.265 (0.151)	0.079	0.088 (0.224)	0.70
BCAS1	rs17216707	T	C	0.041 (0.021)	0.047	0.780 (0.281)	0.0055	0.370 (0.193)	0.055	1.759 (0.287)	9.0×10^{-10}
ARFRP1	rs1758206	T	C	NA	NA	0.459 (0.394)	0.24	0.521 (0.271)	0.055	-0.808 (0.403)	0.045
NRIP1	rs2823139	A	G	0.062 (0.016)	8.7×10^{-5}	0.391 (0.230)	0.088	-0.218 (0.158)	0.17	-0.679 (0.235)	0.0038
ATP50	rs2834317	A	G	0.014 (0.021)	0.49	0.499 (0.302)	0.098	0.210 (0.208)	0.31	0.363 (0.309)	0.24
MKL1	rs17001977	G	A	0.015 (0.026)	0.58	0.380 (0.376)	0.31	0.184 (0.259)	0.48	-0.115 (0.385)	0.76

NA: association summary statistics not reported for outcome. SE: standard error.

[a]Effect allele is aligned to be eGFR decreasing allele.

Effects in grey were excluded from MR analyses as outliers in the causal effect of eGFR on the outcome.

Supplementary Table 10. Association summary statistics for lead eGFR SNVs (non-palindromic) for clinically relevant cardiovascular outcomes on which kidney function has a causal effect in Mendelian randomisation analyses.

Locus	Lead SNV	Alleles		Diastolic blood pressure		Essential (primary) hypertension	
		Effect[a]	Other	Beta (SE) x10^3	*p*-value	Beta (SE) x10^3	*p*-value
CASP9	rs45619934	T	G	3.735 (2.652)	0.16	-0.532 (0.789)	0.50
ZNF436	rs4525087	C	A	-1.596 (2.870)	0.58	-1.816 (0.853)	0.033
RHOC	rs12722725	C	T	-1.178 (3.683)	0.75	-2.375 (1.094)	0.030
ANXA9	rs267738	T	G	1.559 (2.969)	0.60	-1.561 (0.884)	0.077
GBAP1	rs2070803	A	G	6.278 (2.486)	0.012	-1.838 (0.740)	0.013
PMF1-BGLAP	rs2842870	T	C	6.962 (2.563)	0.0066	1.491 (0.763)	0.051
CACNA1S	rs3850625	G	A	-11.185 (3.800)	0.0032	-2.213 (1.132)	0.051
DDX1	rs807603	T	C	-2.302 (2.553)	0.37	-0.321 (0.761)	0.67
NT5C1B-RDH14	rs13417750	A	G	0.475 (2.592)	0.85	1.159 (0.771)	0.13
GCKR	rs1260326	C	T	0.922 (2.517)	0.71	-0.102 (0.749)	0.89
C2orf73	rs1527649	C	T	0.940 (3.084)	0.76	0.590 (0.917)	0.52
NAT8	rs6546869	G	A	-14.148 (2.959)	$1.7x10^{-6}$	-1.296 (0.883)	0.14
PSD4-PAX8	rs11123169	C	T	2.744 (2.661)	0.30	0.119 (0.793)	0.88
ORC4	rs13026220	G	A	14.362 (2.693)	$9.7x10^{-8}$	2.539 (0.802)	0.0015
FAP	rs77335736	C	T	-25.314 (12.474)	0.042	-3.762 (3.688)	0.31
LRP2	rs3770636	T	G	-6.637 (10.898)	0.54	3.728 (3.165)	0.24
NFE2L2	rs35955110	C	T	5.956 (2.661)	0.025	1.743 (0.792)	0.028
CPS1	rs1047891	A	C	-11.189 (2.647)	$2.4x10^{-5}$	-2.185 (0.788)	0.0056
IGFBP5	rs7587010	T	G	-7.980 (2.498)	0.0014	-0.016 (0.745)	0.98
XYLB	rs36070911	G	A	2.250 (2.520)	0.37	-0.367 (0.749)	0.62
SLC15A2	rs2250067	T	C	10.884 (2.466)	$1.0x10^{-5}$	1.719 (0.732)	0.019
TFDP2	rs1511299	T	C	10.554 (2.805)	0.00017	1.289 (0.834)	0.12
ETV5-KNG1	rs13081203	A	G	10.934 (2.591)	$2.5x10^{-5}$	1.184 (0.770)	0.12
RGS12	rs13108218	G	A	-4.779 (2.554)	0.061	-2.765 (0.757)	0.00026
SHROOM3	rs142647267	T	C	NA	NA	NA	NA
PRDM8-FGF5	rs12509595	T	C	-33.342 (2.704)	$6.5x10^{-35}$	-12.201 (0.804)	$5.1x10^{-52}$
NFKB1	rs223401	T	C	14.286 (2.614)	$4.6x10^{-8}$	3.183 (0.776)	$4.2x10^{-5}$
C1QTNF3-AMACR-RAI14	rs10066990	A	G	3.610 (2.546)	0.16	0.036 (0.757)	0.96
DAB2	rs13179493	C	T	-2.809 (2.736)	0.30	-0.925 (0.814)	0.26

ARL15	rs7719168	A	C	0.053 (3.890)	0.99	-1.706 (1.161)	0.14
PIK3R1	rs113246091	A	G	1.108 (4.169)	0.79	-3.289 (1.245)	0.0082
RGS14-SLC34A1	rs3812036	T	C	2.472 (2.886)	0.39	2.242 (0.859)	0.0091
HLA-DRB1-HLA-DQA1	rs117463603	G	A	NA	NA	NA	NA
C6orf1-RPS10-NUDT3	rs6935129	A	G	-5.475 (6.555)	0.40	-4.352 (1.936)	0.025
VEGFA	rs881858	A	G	11.496 (2.662)	$1.6x10^{-5}$	3.778 (0.792)	$1.8x10^{-6}$
AKAP7-ARG1	rs9375818	A	G	1.465 (2.974)	0.62	0.666 (0.886)	0.45
SLC22A2	rs316020	G	A	2.123 (4.052)	0.60	0.813 (1.204)	0.50
UNCX	rs62435145	T	G	9.204 (2.747)	0.00081	3.157 (0.816)	0.00011
AK125311	rs856563	C	T	-6.326 (2.542)	0.013	-1.799 (0.755)	0.017
TMEM60	rs848486	G	A	2.776 (2.494)	0.27	-2.215 (0.741)	0.0028
PRKAG2	rs10265221	C	T	13.710 (2.720)	$4.6x10^{-7}$	4.436 (0.808)	4.0x10-8
SHH	rs6971211	T	C	-1.798 (2.679)	0.50	-2.213 (0.795)	0.0054
STC1	rs7007761	T	C	4.853 (2.500)	0.052	1.310 (0.742)	0.077
NRG1	rs4489283	T	C	0.339 (2.536)	0.89	-0.818 (0.753)	0.28
DCAF12	rs61237993	G	A	0.862 (3.694)	0.82	1.568 (1.095)	0.15
PIP5K1B	rs2039424	G	A	-3.031 (2.538)	0.23	0.535 (0.753)	0.48
ASTN2	rs13283416	G	T	-7.355 (2.494)	0.0032	0.309 (0.740)	0.68
MYPN	rs7475348	C	T	-0.686 (2.469)	0.78	0.557 (0.733)	0.45
CYP26A1	rs4418728	T	G	-2.167 (2.473)	0.38	-1.087 (0.734)	0.14
SUFU	rs6892	A	G	0.344 (3.235)	0.92	-0.194 (0.963)	0.84
FAM53B	rs4962691	T	C	-4.961 (2.503)	0.047	-0.151 (0.743)	0.84
H19-IGF2-KCNQ1	rs7482894	T	C	-1.985 (2.592)	0.44	-0.450 (0.770)	0.56
MPPED2	rs963837	T	C	10.485 (2.469)	$2.2x10^{-5}$	4.111 (0.734)	$2.1x10^{-8}$
CELF1-PTPMT1	rs11039221	T	C	12.756 (2.527)	$4.5x10^{-7}$	5.170 (0.751)	$5.7x10^{-12}$
RNASEH2C	rs11604451	T	C	9.914 (2.587)	0.00013	4.733 (0.769)	$7.5x10^{-10}$
GAB2	rs2063724	T	C	13.169 (3.292)	$6.3x10^{-5}$	3.110 (0.975)	0.0014
SLC6A13	rs10774020	C	T	0.280 (2.615)	0.91	-0.807 (0.776)	0.30
TSPAN9	rs632887	G	A	-1.014 (2.532)	0.69	-0.686 (0.751)	0.36
KRR1-PHLDA1	rs1275609	G	A	1.708 (2.672)	0.52	0.236 (0.792)	0.77
CUX2	rs79105258	A	C	NA	NA	-58.526 (62.366)	0.35
DGKH	rs34445998	C	T	4.066 (4.029)	0.31	-1.307 (1.193)	0.27
DACH1	rs584480	C	T	-3.425 (2.523)	0.17	-1.298 (0.747)	0.082
RASGRP1	rs9920185	C	A	1.203 (2.486)	0.63	0.043 (0.737)	0.95

GATM	rs2486288	C	T	2.957 (2.551)	0.25	0.418 (0.755)	0.58
UBE2Q2	rs11636251	T	C	1.908 (2.466)	0.44	1.406 (0.731)	0.054
CEMIP	rs62035088	G	A	2.892 (3.284)	0.38	0.010 (0.976)	0.99
IGF1R	rs11858316	C	T	0.904 (2.525)	0.72	1.247 (0.748)	0.096
UMOD-PDILT	rs77924615	G	A	22.895 (3.117)	$2.1x10^{-13}$	8.019 (0.925)	$4.5x10^{-18}$
SALL1	rs12935539	C	T	-9.295 (2.958)	0.0017	-4.598 (0.879)	$1.7x10^{-7}$
SLC7A6	rs9888796	T	C	8.828 (2.822)	0.0018	1.638 (0.836)	0.050
NFAT5	rs11641050	C	T	-1.228 (3.157)	0.70	-1.311 (0.934)	0.16
DPEP1	rs2460449	A	G	-9.443 (2.498)	0.00016	-2.537 (0.740)	0.00060
SLC47A1	rs11871125	T	C	3.036 (2.534)	0.23	0.897 (0.752)	0.23
BCAS3	rs9895661	C	T	-4.208 (3.288)	0.20	-5.351 (0.972)	$3.7x10^{-8}$
EPB41L3	rs1719934	G	A	2.960 (2.475)	0.23	1.040 (0.733)	0.16
PCAT18-AQP4	rs16942751	A	C	-12.227 (4.393)	0.0054	0.233 (1.299)	0.86
RNF152	rs896642	C	T	2.779 (2.983)	0.35	2.132 (0.887)	0.016
JUND-LSM4	rs8108623	A	C	-0.078 (2.695)	0.98	-0.152 (0.799)	0.85
SLC7A9	rs7252778	A	C	3.139 (2.519)	0.21	-0.715 (0.746)	0.34
TP53INP2	rs2273684	G	T	0.760 (2.495)	0.76	-0.539 (0.740)	0.47
BCAS1	rs17216707	T	C	2.098 (3.190)	0.51	1.021 (0.947)	0.28
ARFRP1	rs1758206	T	C	7.549 (4.488)	0.093	1.877 (1.329)	0.16
NRIP1	rs2823139	A	G	7.251 (2.615)	0.0056	3.377 (0.774)	$1.3x10^{-5}$
ATP50	rs2834317	A	G	5.334 (3.438)	0.12	0.047 (1.016)	0.96
MKL1	rs17001977	G	A	19.739 (4.255)	$3.5x10^{-6}$	0.697 (1.266)	0.58

NA: association summary statistics not reported for outcome. SE: standard error.

[a]Effect allele is aligned to be eGFR decreasing allele.

Effects in grey were excluded from MR analyses as outliers in the causal effect of eGFR on the outcome.

Supplementary Table 11. Two sample Mendelian randomisation analyses to assess causal effect of eGFR on diastolic blood pressure, based on association summary statistics for lead SNVs obtained from ICBP (150,134 individuals).

Component of trans-ethnic meta-analysis	Sample size	SNVs	Inverse variance weighted regression				Weighted median regression			MR-EGGER regression			
			beta	SE	*p*-value	*Q p*-value	beta	SE	*p*-value	beta	SE	*p*-value	*Q p*-value
COGENT-Kidney Consortium	58,293	45	0.015	0.026	0.56	0.00054	-0.036	0.071	0.28	-0.078	0.071	0.28	0.00092
CKDGen Consortium	110,517	44	0.999	1.604	0.53	0.0035	-1.057	1.927	0.58	-0.461	3.938	0.91	0.0028
Biobank Japan Project	145,658	43	0.343	0.383	0.37	0.018	-0.447	0.475	0.35	0.516	1.250	0.68	0.014

SE: standard error.

Supplementary Table 12. Sample characteristics of GWAS contributing to the COGENT-Kidney Consortium.

Study (acronym)	Ethnicity (Country of origin)	Sex	Sample size	Age (years) mean (SD)	Serum Creatinine (mg/dL) mean (SD)	eGFR mean (SD)
Australian Twin-Family Studies (AUSTWIN)	European (Australia)	Males	4,662	48.7 (13.1)	1.13 (0.20)	76.6 (15.8)
		Females	7,096	46.9 (13.4)	0.90 (0.16)	75.1 (16.5)
BioBank Japan Project (BBJ)	East Asian (Japan)	Males	12,802	64.4 (9.8)	0.89 (0.29)	100.2 (28.5)
		Females	10,734	60.7 (13.1)	0.64 (0.20)	109.1 (31.0)
BioME Biobank (BioME-HAAA)	Hispanic/Latino (USA)	Males	1,052	56.1 (15.2)	1.23 (1.13)	81.1 (27.3)
		Females	1,697	55.7 (15.9)	0.91 (0.60)	79.6 (25.0)
BioME Biobank (BioME-HANA)	Hispanic/Latino (USA)	Males	160	54.2 (15.3)	1.20 (1.20)	83.5 (25.2)
		Females	194	52.0 (17.1)	0.89 (0.58)	87.3 (39.1)
Genetics of Latinos Diabetic Retinopathy (GOLDR)	Hispanic/Latino (USA)	Males	223	52.2 (10.8)	1.05 (0.88)	103.1 (36.2)
		Females	374	53.3 (10.7)	0.86 (0.99)	100.9 (33.4)
Hispanic Community Health Study and Study of Latinos (HCHS/SOL)	Hispanic/Latino (USA)	Males	5,179	45.3 (14.2)	0.98 (0.43)	95.5 (22.3)
		Females	7,420	46.7 (13.6)	0.73 (0.23)	96.6 (23.4)
Mexican American Hypertension and Insulin Resistance (HTNIR)	Hispanic/Latino (USA)	Males	293	40.2 (15.1)	0.88 (0.20)	109.5 (28)
		Females	428	40.3 (14.4)	0.67 (0.16)	112.5 (30.9)
Mexico City (MC11)	Hispanic/Latino (Mexico)	Males	195	54.6 (8.3)	N/A	98.3 (26.3)
		Females	391	53.9 (7.9)	N/A	103.1 (32.7)
Mexico City (MC12)	Hispanic/Latino (Mexico)	Males	97	42.7 (7.7)	N/A	66.0 (15.2)
		Females	264	45.4 (7.6)	N/A	61.0 (12.2)
Mexico City (MC2A)	Hispanic/Latino (Mexico)	Males	360	57.6 (11.3)	N/A	91.6 (28.1)
		Females	535	56.5 (9.1)	N/A	91.2 (23.2)
Mexico City (MC2U)	Hispanic/Latino (Mexico)	Males	529	47.8 (8.1)	N/A	104.5 (22.7)
		Females	359	49.7 (8.4)	N/A	94.5 (21.6)
Multi-Ethnic Study of Atherosclerosis (MESA)	Hispanic/Latino (USA)	Males	960	60.3 (10.2)	1.01 (0.36)	85.0 (18.4)
		Females	1123	61.3 (9.9)	0.80 (0.24)	81.4 (17.8)
Northern Manhattan Family Study (NOMAS)	Hispanic/Latino (USA)	Males	366	68.2 (7.9)	1.10 (0.80)	75.8 (17.8)
		Females	596	68.7 (8.7)	0.80 (0.20)	75.7 (17.1)
Prospective Investigation of the Vasculature in Uppsala Seniors (PIVUS)	European (Sweden)	Males	471	70.1 (0.1)	0.99 (0.22)	83.8 (19.9)
		Females	473	70.2 (0.2)	0.82 (0.18)	77.9 (20.2)
Uppsala Longitudinal Study of Adult Men (ULSAM)	European (Sweden)	Males	1,080	71.0 (0.6)	1.06 (0.15)	75.2 (11.3)
		Females	0	N/A	N/A	N/A

Women's Health Initiative Genome-wide Association Research Network into Effects of Treatment (WHI-GARNET)	European (USA)	Males	0	N/A	N/A	N/A
		Females	4,116	65.6 (6.9)	0.74 (0.15)	88.1 (19.3)
Women's Health Initiative Memory Study (WHIMS+)	European (USA)	Males	0	N/A	N/A	N/A
		Females	5,655	68.1 (5.9)	0.75 (0.15)	85.6 (17.8)
Women's Health Initiative SNP Health Association Resource: African Americans (WHI-SHARe-A)	African American (USA)	Males	0	N/A	N/A	N/A
		Females	8,224	61.6 (7.0)	0.82 (0.22)	80.1 (19.4)
Women's Health Initiative SNP Health Association Resource: Hispanic Americans (WHI-SHARe-H)	Hispanic/Latino (USA)	Males	0	N/A	N/A	N/A
		Females	3,549	60.3 (6.7)	0.71 (0.19)	94.7 (21.9)

SD: standard deviation.

Supplementary Table 13. Summary of study-specific genotyping, quality control, imputation and analysis of GWAS contributing to the COGENT-Kidney Consortium.

Study acronym	Genotyping array	Sample QC		Scaffold QC		Pre-phasing and imputation				Association analysis		
		Call rate	Exclusions	Call rate	HWE *p*	MAF	Software	Quality filter	Passed SNVs	Software	Covariates	λ_{GC}
AUSTWIN	Illumina 317K, 370K, 610K, OmniExpress, Omni2.5M, CoreExome	95%	Heterozygosity, gender check, relatedness	95%	10^{-6}	1%	MaCH minimac	$r^2 \geq 0.3$	7,189,355	MERLIN	age, sex, sub-study, 10 PCs	1.121
BBJ	Iluumina 610K	98%	Ethnicity, relatedness	99%	10^{-7}	1%	MaCH minimac	$r^2 \geq 0.5$	5,923,453	mach2qtl	none	1.056
BioME-HAAA	Illumina OmniExpress	99%	Ethnicity, gender check, relatedness, duplicates	95%	10^{-5}	1%	SHAPEIT v2 IMPUTEv2	info≥0.4	14,395,680	SNPTESTv2	age, sex, 6 PCs	0.963
BioME-HANA	Illumina OmniExpress	99%	Ethnicity, gender check, relatedness, duplicates	95%	10^{-5}	1%	SHAPEIT v2 IMPUTEv2	info≥0.4	5,248,638	SNPTESTv2	age, sex, 6 PCs	0.944
GOLDR	Illumina OmniExpress	95%	Gender check, relatedness, duplicates	95%	10^{-6}	1%	minimac3	info≥0.4	6,890,696	EPACTS	age, sex, 3 PCs	1.059
HCHS/SOL	Illumina Omni2.5M, custom	98%	Gender check, duplicates	98%	10^{-5}	None	SHAPEITv2 IMPUTEv2	info≥0.4	17,462,761	LMM-OPS[a]	age, sex, centre, sampling weights, 5 PCs	0.983
HTNIR	Illumina OmniExpress	95%	Gender check, relatedness	95%	10^{-6}	1%	IMPUTEv2	info≥0.5	6,393,429	GWAF	age, sex, admixture	0.944
MC11	Affymetrix 5.0	95%	Gender check, relatedness, duplicates	95%	10^{-6}	1%	IMPUTEv2	info≥0.3	5,431,276	SNPTESTv2	age, sex, 1 PC	1.010
MC12	Affymetrix 5.0	95%	Gender check, relatedness, duplicates	95%	10^{-6}	1%	IMPUTEv2	info≥0.3	5,776,583	SNPTESTv2	age, sex, 1 PC	0.999
MC2A	Affymetrix Axiom LAT	95%	Gender check, relatedness, duplicates	95%	10^{-6}	1%	IMPUTEv2	info≥0.3	6,861,608	SNPTESTv2	age, sex, 1 PC	0.987
MC2U	Affymetrix Axiom LAT	95%	Gender check, relatedness, duplicates	95%	10^{-6}	1%	IMPUTEv2	info≥0.3	7,042,576	SNPTESTv2	age, sex, 1 PC	0.995
MESA	Affimetrix 6.0	95%	Gender check, duplicates	95%	10^{-5}		minimac3	$r^2 \geq 0.3$	12,140,096	ANYGWAS[b]	age, sex, 4 PCs	0.968
NOMAS	Affymetrix 6.0	95%	Ethnicity, gender check, relatedness, duplicates	95%	10^{-6}	1%	IMPUTEv2	info≥0.4	10,353,282	PLINK	age, 3 PCs	0.997
PIVUS	Illumina OmniExpress, Metabochip	95%	Heterozygosity, gender check, relatedness	95% (99% if MAF<5%)	10^{-6}	1%	SHAPEITv2 IMPUTEv2	info≥0.4	7,470,949	SNPTESTv2	age, sex, 2 PCs	0.989
ULSAM	Illumina Omni2.5M, Metabochip	95%	Heterozygosity, gender check, relatedness	95% (99% if MAF<5%)	10^{-6}	1%	SHAPEITv2 IMPUTEv2	info≥0.4	7,619,548	SNPTESTv2	age, 2 PCs	1.018
WHI-GARNET	Illumina Omni1M	None	Ethnicity, gender check, relatedness, duplicates	98%	10^{-4}	None	Beagle minimac	$r^2 \geq 0.3$	7,326,751	ProbAbel/R	age, centre, 10 PCs	1.018
WHIMS+	Illumina OmniExpress-Exome	None	Ethnicity, gender check, relatedness, duplicates	97%	10^{-4}	1%	Beagle minimac	$r^2 \geq 0.3$	7,314,486	ProbAbel/R	age, centre, 10 PCs	1.024
WHI-SHARe-A	Affymetrix 6.0	95%	Ethnicity, gender check, relatedness, duplicates	95%	10^{-6}	1%	MaCH	$r^2 \geq 0.3$	13,096,173	ProbAbel	age, centre, 10 PCs	1.034
WHI-SHARe-H	Affymetrix 6.0	95%	Ethnicity, gender check, relatedness, duplicates	95%	10^{-6}	1%	MaCH	$r^2 \geq 0.3$	8,407,769	ProbAbel	age, centre, 10 PCs	1.027

QC: quality control. HWE: Hardy-Weinberg equilibrium. MAF: minor allele frequency. PC: principal component.

[a]In-house software, not yet publicly available; accounts for relatedness in linear mixed model.

[b]In-house software, not yet publicly available; accounts for relatedness in linear mixed model using R nlme package.

Supplementary Note 1. Additional acknowledgements

Australian Twin-Family Studies (AUSTWIN). We acknowledge funding from the Australian National Health and Medical Research Council (241944, 389875, 389891,389892, 389938, 442915, 442981, 496739 and 552485), US National Institutes of Health (AA07535, AA10248 and AA014041) and the Australian Research Council (DP0770096).

BioME Biobank (BioME-HAAA and BioME-HANA). The Mount Sinai BioME Biobank is supported by The Andrea and Charles Bronfman Philanthropies.

Genetics of Latinos Diabetic Retinopathy (GOLDR). GOLDR was supported in part by the Genetics of Latinos Diabetic Retinopathy (GOLDR) Study grant EY14684. The provision of genotyping data was supported in part by the National Center for Advancing Translational Sciences, CTSI grant, UL1TR001881, and the National Institute of Diabetes and Digestive and Kidney Disease Diabetes Research Center (DRC) grant DK063491 to the Southern California Diabetes Research Center.

Hispanic Community Health Study and Study of Latinos (HCHS/SOL). HCHS/SOL was carried out as a collaborative study supported by contracts from the National Heart, Lung, and Blood Institute (NHLBI) to the University of North Carolina (N01-HC-65233), University of Miami (N01-HC-65234), Albert Einstein College of Medicine (N01-HC-65235), Northwestern University (N01-HC-65236), and San Diego State University (N01-HC-65237). The following contribute to the HCHS/SOL through a transfer of funds to the NHLBI: National Center on Minority Health and Health Disparities, the National Institute of Deafness and Other Communications Disorders, the National Institute of Dental and Craniofacial Research (NIDCR), the National Institute of Diabetes and Digestive and Kidney Diseases, the National Institute of Neurological Disorders and Stroke, and the Office of Dietary Supplements. The Genetic Analysis Center at the University of Washington was supported by NHLBI and NIDCR contracts (HHSN268201300005C AM03 and MOD03).

Mexican American Hypertension and Insulin Resistance (HTNIR). This work was supported by the National Institutes of Health National Heart, Lung, and Blood Institute (grant HL55005); the National Institutes of Health National Center for Research Resources, General Clinical Research Center (grants RR00425 to RR00430, M01-RR00865, M01-RR00043, M01-RR00425, and CA42710). The authors thank the families for their participation in this study.

Mexico City Studies (MC11, MC12, MC2A and MC2U). In Mexico, this work was supported by the Fondo Sectorial de Investigación en Salud y Seguridad Social (SSA/IMSS/ISSSTE-CONACYT, project 150352), Temas Prioritarios de Salud Instituto Mexicano del Seguro Social (2014-FIS/IMSS/PROT/PRIO/14/34), and the Fundación IMSS. We thank Jorge Gutierrez Cuevas, Jaime Gómez Zamudio and Araceli Méndez Padrón for technical support. In Canada, computations were performed on the GPC supercomputer at the SciNet HPC Consortium. SciNet is funded by: the Canada Foundation for Innovation under the auspices of Compute Canada; the Government of Ontario; Ontario Research Fund - Research Excellence; and the University of Toronto. Esteban J Parra was supported by the Canadian Institutes of Health Research and the Banting and Best Diabetes Center.

Multi-Ethnic Study of Atherosclerosis (MESA). MESA and the MESA SHARe project are conducted and supported by the National Heart, Lung, and Blood Institute (NHLBI) in collaboration with MESA investigators. Support for MESA is provided by contracts HHSN268201500003I, N01-HC-95159, N01-HC-95160, N01-HC-95161, N01-HC-95162, N01-HC-95163, N01-HC-95164, N01-HC-95165, N01-HC-95166, N01-HC-95167, N01-HC-95168, N01-HC-95169, UL1-TR-000040, UL1-TR-001079, UL1-TR-001420, UL1-TR-001881, and DK-063491. MESA Family is conducted and supported by the NHLBI in collaboration with MESA investigators. Support is provided by grants and contracts R01-HL-071051, R01-HL-071205, R01-HL-071250, R01-HL-071251, R01-HL-071258, R01-HL-071259, by the National Center for Research Resources (UL1-RR-033176), and the National Center for Advancing Translational Sciences (UL1-TR-001881). Funding for SHARe genotyping was provided by NHLBI contract N02-HL-64278.

Northern Manhattan Family Study (NOMAS). This work was supported by the NIH (R01-NS-065114 and R01-NS-29993).

Prospective Investigation of the Vasculature in Uppsala Seniors (PIVUS) and Uppsala Longitudinal Study of Adult Men (ULSAM). These projects were supported by Knut and Alice Wallenberg Foundation (Wallenberg Academy Fellow), European Research Council (ERC Starting Grant), Swedish Diabetes Foundation (2013-024), Swedish Research Council (2012-1397, 2012-1727, and 2012-2215), Marianne and Marcus Wallenberg Foundation, County Council of Dalarna, Dalarna University, and Swedish Heart-Lung Foundation (2012-0197). The computations were performed on resources provided by SNIC through Uppsala Multidisciplinary Center for Advanced Computational Science (UPPMAX) under Project b2011036. Genotyping was funded by the Wellcome Trust under award WT064890. Analysis of genetic data was funded by the Wellcome Trust under awards WT098017 and WT090532. We thank the SNP&SEQ Technology Platform in Uppsala (www.genotyping.se) for excellent genotyping.

Women's Health Initiative (WHI-GARNET, WHIMS+, WHI-SHARe-A, WHI-SHARe-H). The WHI program is funded by the National Heart, Lung, and Blood Institute through contracts HHSN268201600018C, HHSN268201600001C, HHSN268201600002C, HHSN268201600003C, and HHSN268201600004C. We thank the WHI investigators and staff for their dedication, and the study participants for making the program possible. A full listing of WHI investigators can be found at: http://www.whi.org/researchers/Documents%20%20Write%20a%20Paper/WHI%20Investigator%20Long%20List.pdf

TRANSLATE Study. The TRANScriptome of renaL humAn TissuE (TRANSLATE) study is supported by British Heart Foundation (17/35/33001) and Kidney Research UK (RP_017_20180302). We thank all the recruiting centers and the participants for their important contributions. Access to The Cancer Genome Atlas (TCGA) has been approved by TCGA DAC (approval #50804-2).

IgA Nephropathy GWAS. We acknowledge the multiple clinical centers that contributed patients and/or data to the GWAS for IgA nephropathy used in this study: the work was supported by the NIH (R01-DK-105124, R01-DK-082753 and RC2-DK-116690).

International Consortium of Blood Pressure (ICBP). We thank Louise Wain and the ICBP for provision of association summary statistics for blood pressure for Mendelian randomisation analyses.
